# Impact of fortified versus unfortified lipid-based supplements on morbidity and nutritional status: A randomised double-blind placebo-controlled trial in ill Gambian children

**DOI:** 10.1371/journal.pmed.1002377

**Published:** 2017-08-15

**Authors:** Stefan A. Unger, Saikou Drammeh, Jahid Hasan, Kabiru Ceesay, Edrisa Sinjanka, Sainey Beyai, Bakary Sonko, Bai Lamin Dondeh, Anthony J. Fulford, Sophie E. Moore, Andrew M. Prentice

**Affiliations:** 1 MRC Unit The Gambia, Banjul, The Gambia; 2 MRC International Nutrition Group, London School of Hygiene & Tropical Medicine, London, United Kingdom; 3 University of Edinburgh, Department of Child Life and Health, Edinburgh, United Kingdom; 4 Division of Women’s Health, King’s College London, London, United Kingdom; Uppsala University, SWEDEN

## Abstract

**Background:**

Multiple micronutrients (MMN) are commonly prescribed in pediatric primary healthcare in sub-Saharan Africa to improve nutritional status and appetite without evidence for their effectiveness or international clinical guidelines. Community-wide MMN supplementation has shown limited and heterogeneous impact on growth and morbidity. Short-term ready-to-use therapeutic foods in acutely sick children in a hospital setting also had limited efficacy regarding subsequent growth. The effectiveness of MMN in improving morbidity or growth in sick children presenting for primary care has not been assessed.

**Methods and findings:**

We undertook a double-blind randomised controlled trial of small-quantity lipid-based nutrient supplements (SQ-LNS) fortified with 23 micronutrients in children aged 6 months (mo) to 5 years (y) presenting with an illness at a rural primary healthcare centre in The Gambia. Primary outcomes were repeat clinic presentations and growth over 24 wk. Participants were randomly assigned to receive 1 of 3 interventions: (1) supplementation with micronutrient-fortified SQ-LNS for 12 wk (MMN-12), (2) supplementation with micronutrient-fortified SQ-LNS for 6 wk followed by unfortified SQ-LNS for 6 wk (MMN-6), or (3) supplementation with unfortified SQ-LNS for 12 wk (MMN-0) to be consumed in daily portions. Treatment masking used 16 letters per 6-wk block in the randomisation process. Blinded intention-to-treat analysis based on a prespecified statistical analysis plan included all participants eligible and correctly enrolled.

Between December 2009 and June 2011, 1,101 children (age 6–60 mo, mean 25.5 mo) were enrolled, and 1,085 were assessed (MMN-0 = 361, MMN-6 = 362, MMN-12 = 362). MMN supplementation was associated with a small increase in height-for-age z-scores 24 wk after recruitment (effect size for MMN groups combined: 0.084 SD/24 wk, 95% CI: 0.005, 0.168; *p* = 0.037; equivalent to 2–5 mm depending on age). No significant difference in frequency of morbidity measured by the number of visits to the clinic within 24 wk follow-up was detected with 0.09 presentations per wk for all groups (MMN-0 versus MMN-6: adjusted incidence rate ratio [IRR] 1.03, 95% CI: 0.92, 1.16; MMN-0 versus MMN-12: 1.05, 95% CI: 0.93, 1.18). In post hoc analysis, clinic visits significantly increased by 43% over the first 3 wk of fortified versus unfortified SQ-LNS (adjusted IRR 1.43; 95% CI: 1.07, 1.92; *p* = 0.016), with respiratory presentations increasing by 52% with fortified SQ-LNS (adjusted IRR 1.52; 95% CI: 1.01, 2.30; *p* = 0.046). The number of severe adverse events during supplementation were similar between groups (MMN-0 = 20 [1 death]; MMN-6 = 21 [1 death]; MMN-12 = 20 [0 death]). No participant withdrew due to adverse effects. Study limitations included the lack of supervision of daily supplementation.

**Conclusion:**

Prescribing micronutrient-fortified SQ-LNS to ill children presenting for primary care in rural Gambia had a very small effect on linear growth and did not reduce morbidity compared to unfortified SQ-LNS. An early increase in repeat visits indicates a need for the establishment of evidence-based guidelines and caution with systematic prescribing of MMN. Future research should be directed at understanding the mechanisms behind the lack of effect of MMN supplementation on morbidity measures and limited effect on growth.

**Trial registration:**

ISRCTN 73571031.

## Abstract 2

In a randomised double-blind placebo-controlled trial, Stefan Unger and colleagues examine the impact of fortified versus unfortified lipid-based supplements on morbidity and nutritional status in ill Gambian children.

## Author summary

**Why was this study done?:**

- Multiple micronutrients (MMN) are prescribed widely in low- and middle-income settings.
- There are currently no established evidence-based international clinical guidelines for the use of MMN in ill children.
- Community-wide trials of MMN within a lipid-base supplement have shown improvement in linear growth in some settings, although the effect on morbidity is underresearched.
- Our study was designed to investigate the effect of lipid-based MMN in ill children in relation to the frequency of subsequent primary health clinic visits and growth.

**What did the research do and find?:**

- We performed a prospective, double-blind randomised controlled trial of small-quantity lipid-based MMN in ill children presenting to a rural primary healthcare clinic in West Africa.
- We enrolled 1,100 children aged 6 months (mo) to 5 years (y).
- Children were supplemented either with the lipid-base alone or a micronutrient-enriched lipid-based supplement for 6 wk or 12 wk.
- The trial showed no effect of small-quantity lipid-based MMN supplementation compared to unfortified supplement on subsequent all-cause morbidity as assessed by clinic visits over 6 mo, but post hoc analysis revealed a significant 43% increase in clinic presentations over the first 3 wk of supplementation.
- Despite a significant increase in children’s reported appetite, there was only a small benefit on linear growth.

**What do these findings mean?:**

- Randomised trials have so far failed to demonstrate consistent beneficial effects of MMN supplementation (lipid-based or not) on growth and morbidity, and there is some evidence of potentially harmful effects, likely related to iron in the supplements.
- Blanket prescribing of MMN in primary health clinics in similar settings is not supported by current evidence.

## Introduction

It has been estimated that vitamin and mineral deficiencies affect more than 2,000,000,000 people worldwide. Together with other manifestations of malnutrition (fetal growth restriction, stunting, and low breastfeeding rates), such deficiencies contribute to over 3 million child deaths worldwide; approximately 45% of the total. [1]

In recognition of the fact that children in deprived populations tend to suffer from a combination of nutrient deficiencies, recent intervention programs have favoured the use of multiple micronutrients (MMN). MMN are also widely, and often indiscriminately, prescribed in primary healthcare clinics, despite the absence of a supporting evidence base. [2]

MMN are available as liquids, dispersible tablets, micro-encapsulated powders, or embedded in lipid-based nutrient supplements (LNS). Lipid-based MMN supplements may lower the risk of overdosing, address macro- as well as micronutrient deficiencies, and can enhance the absorption of fat-soluble vitamins.[3] Despite these potential benefits, the recently-reported trials of community-based LNS distribution in Burkino-Faso,[4] Malawi,[5,6] Bangladesh [7] and Ghana [3,8] show a limited and heterogeneous impact on growth and development. Short-term ready-to-use therapeutic foods (RUTF) supplementation targeted at nonmalnourished but acutely sick children also had limited efficacy and only in one of the settings studied. [9,10] (S3 Text).

Because of the tight interplay between nutrition and infection [11], we reasoned that by targeting ill children who would have a greater need of nutrient replenishment, clinic-based distribution of small-quantity lipid-based nutrient supplements (SQ-LNS) might be more effective, efficient, and easily implementable. We tested this hypothesis in a randomised clinical trial in children presenting to a rural primary healthcare clinic in sub-Saharan Africa. Primary outcomes were growth and morbidity, measured as repeat presentations to the clinic.

## Methods

### Ethics statement

Ethics approval was granted by the joint Gambian Government/Medical Research Council (MRC) Unit The Gambia Ethics Committee (SCC1135v2, L2009.58, L2009v2) and the London School of Hygiene and Tropical Medicine Ethics Committee (5629) (S2 Text). Community approval to run this trial in Kiang West was given after a series of meetings between the principle investigator and community leaders in each village, including the Alkalos (village heads) and Imams (Islamic clerics).

### Study design and participants

We undertook a double-blind randomised placebo-controlled trial of a SQ-LNS in children presenting to a primary healthcare clinic in Kiang West an isolated, rural district of The Gambia. The trial was conducted to good clinical practice (GCP) standards.

Kiang West has a population of around 15,000. Rural subsistence farming is the main livelihood, which is heavily influenced by the unimodal annual rainy season. The under-5 mortality rate is lower than the national average (45 versus 54 per 1,000 live births). [12] The population is served by 1 main primary healthcare centre in Keneba, run by the MRC, and 2 smaller government-run health stations. The Keneba primary healthcare clinic, located centrally within Kiang West, provides free general healthcare to all Kiang West citizens. Around 1,500 residents excluding visitors from outside Kiang West are seen at the clinic each month.

The Keneba Electronic Medical Records System (KEMReS) was developed as an enabling technology to capture clinical data. [12] KEMReS is linked to the Kiang West Demographic Surveillance System (KWDSS), a database containing basic demographic data with unique identification numbers for all residents.

All Kiang West children aged 6 to 60 months (mo) presenting with an acute illness to the Keneba primary healthcare centre were eligible for inclusion in the study. Children enrolled in another research study, or those with known haemoglobinopathies or severe malnutrition were excluded. Children with severe malnutrition as defined by the World Health Organization (WHO) [13] were treated at the MRC Keneba nutritional rehabilitation centre and then became eligible for enrolment at a subsequent self-referred presentation after successful management. Children and infants called for routine child welfare clinics, clinical follow-up appointments, and immunisations were excluded unless they presented with an acute illness. Children were eligible for enrolment at a subsequent self-referred presentation.

A doctor assessed all presenting children and completed an eligibility form. A trial team member then provided information about the study to the caregiver of the child, and oral and written consent were taken before enrolment.

All children enrolled in the trial were followed-up daily for 1 week (wk) and then fortnightly until the end of the 12-wk supplementation period to gather caregiver-reported morbidity and compliance data. Anthropometry measurements were taken at recruitment and 6 wk, 12 wk 24 wk, and 24 mo after enrolment. Re-presentation with any illness to one of the 3 clinics in Kiang West was monitored over 6 mo after enrolment.

### Randomisation and masking

Participants were randomised into 3 groups: (1) supplementation with micronutrient-fortified SQ-LNS for 12 wk (MMN-12); (2) supplementation with micronutrient-fortified SQ-LNS for 6 wk followed by unfortified SQ-LNS for 6 wk (MMN-6); or (3) supplementation with unfortified SQ-LNS for 12 wk (MMN-0). Balance with respect to date of recruitment was achieved using block randomisation (blocks of 12). Participants were allocated their randomisation code according to sequence of presentation.

The third, eighth, thirteenth, and subsequently every fifth child within each supplement group were assigned to a subgroup for micronutrient status measurements at the end of the 12-wk supplementation period (measurement of haemoglobin and plasma zinc, retinol, 25(OH) vitamin D (25(OH)D) and selenium).

Supplement jars were identical apart from their label, using 16 letters per 6-wk supplementation period to minimise bias. The code was only known to the MMN producer (Valid International, Kenya) and an independent person. Investigators, study team members, statistician, and participants were masked to the composition of the supplementation until after the results of the analysis according to the preagreed statistical analysis plan were available.

### Procedures

The intervention was a SQ-LNS developed in collaboration with Valid International, Nairobi, Kenya. The SQ-LNS contained 23 micronutrients (S1 Table). Two different fortified spreads were used, identifiable by the colour of the cap of the supplement: 1 for the age groups 6–12 mo (SQ-LNS-MMN-1) and 1 for the age group 1–5 y (SQ-LNS-MMN-2), adjusting for the differences in Recommended Daily Allowance (RDA)/Recommended Nutrient Intake (RNI) at different ages. SQ-LNS-MMN-0 was the lipid-based paste without added MMN.

To mimic a possible programme setting, a 14-day supply of supplement was given to the caregiver of the enrolled children every 2 wk. The supplement was supplied in jars containing 60 g of LNS. Supplementation of the child was not observed by a study team member. Trial members were trained in delivering clear instructions on how to supplement the children. Caregivers were asked to administer SQ-LNS on a daily basis by mixing 4 levelled teaspoons of supplement (equivalent to 20 g of SQ-LNS) with the child’s food. Self-reported noncompliance was defined as any participant consuming less than 70% intended. Advice was given when to present to the primary healthcare facility for an illness and to continue with the supplementation unless advised otherwise by a member of the medical team.

After the initial daily visits for morbidity data collection during the first wk of supplementation, a team of trained trial fieldworkers (FWs) visited each child fortnightly during the 12-wk supplementation period, recording morbidity using data collection procedures previously trialled in the study population. FWs also counted used supplementation jars and replenished the supplement held by the caregiver to provide the next 14-day dose. At each visit to the child’s family, the caregiver was interviewed to obtain information on child’s appetite, presence or absence of diarrhoea, vomiting, cough, rapid breathing, and perceived fever in the preceding 72 h. Diarrhoea was defined as 3 or more loose stools per day.

Anthropometric measures were undertaken by trained trial staff and clinic team members using standardised methods. Two sets of anthropometric data were taken and entered into the database. If they were the same or within the accepted error range, the first data set was used. If the data differed, measurement had to be repeated. Quality control checks were set within the electronic patient record system and performed according to standardised operating procedure (S5 Text). Trial staff received repeated mini-assessments and refresher training throughout the trial. Length or height was measured using a Harpenden Infantometer length board (Holtain Ltd) or stadiometer (Leicester Height Measure; model MKII), respectively. Weight was measured using digital scales (Seca, model 336; sensitivity 0.01 kg for infants or Tanita HD-305 scale; sensitivity 0.1 kg, for older children). Mid-upper arm circumference (MUAC) was measured using a paper measuring tape to a precision of 0.1 cm. Measures were expressed as z-scores based on the WHO 2006 Growth Standard [14], and effects of MMN supplementation were calculated based on internal growth reference data collected in Kiang West Keneba clinic between 12 December 2009 and 2 June 2012. Anthropometry equipment was checked weekly.

Children self-referred to the clinic were assessed each time by 1 of 3 medical officers using a standardised proforma within KEMReS. [12] Based on previous data and safety concerns with regards to iron supplementation, [15] supplementation was withheld in children presenting with severe malaria, severe sepsis, or severe anaemia until successful management of the condition; severity was classified as requiring admission/referral to a hospital and the need for intravenous (IV) quinine/artesunate, IV antibiotics, and blood transfusions, respectively. All children presenting to Keneba were treated in accordance with the Gambian/WHO clinical guidelines. [13] Additional morbidity data on presentations at government-run health centres in Kiang West were also collected.

Venous blood was collected at the end of the supplementation period at 12 wk in the subset selected for micronutrient status analysis. Haemoglobin concentration was measured using an automated haematology analyser (Medonic M-series 3-part haematology analyser [Boule Medical]). Plasma was separated and stored at −70°C. Samples were then transported to MRC Human Nutrition Research (HNR), Cambridge, United Kingdom on dry ice and analysed for plasma zinc and selenium using inductively coupled plasma-mass spectrometry. Plasma retinol levels were analysed by high-performance liquid chromatography and plasma 25(OH)D by a chemiluminescent assay. Plasma C-reactive protein (CRP) was measured using a commercial colorimetric immunoassay, and alpha(1)-acid glycoprotein (AGP) was measured by an immunoturbidimetric specific reaction (Sentinel Diagnostics). Zinc, selenium, retinol, and 25(OH)D levels were adjusted for CRP and AGP in the analysis.

### Outcomes

Our primary outcomes were morbidity measured as presentations to a clinic in Kiang West within 6 mo and growth at 6 mo after enrolment.

Secondary outcomes included the following: (1) caregiver-reported morbidity collected daily for the first wk and then fortnightly during the 12-wk supplementation period; (2) repeat presentation to clinic (separated into those diagnosed with diarrhoeal, acute respiratory, malarial, or skin infection); (3) micronutrient status for zinc, retinol, selenium, and 25(OH)D and haemoglobin levels at the end of supplementation; (4) caregiver-reported appetite during supplementation; and (5) growth at 2 y after enrolment.

Any self-referred clinic visit was recorded as a potential adverse event, and a summary of events was sent at agreed intervals to the trial and data safety monitors. Any clinic presentations with severe illness or any deaths were registered as severe adverse events, and the data safety monitor was informed within 24 h, followed by a detailed description and assessment of each case. Any visits to clinics outside Kiang West were recorded as part of the 2 wk FW visit.

### Statistical analysis

There were no published data on the effect of nutritional intervention on repeat clinic visits in this age group on which to base a power calculation. Prior to the introduction of KEMReS, limited data was available on clinic presentations at MRC Keneba in patients over 3 y old and from those residents outside the ‘core villages’ in Kiang West. [12] We originally estimated that there were over 1,000 clinic visits for the age group 6–12 mo old and 2,000 clinic visits for the age group 1–5 y old per y, with approximately 25% of clinic visits being repeat visits—i.e., return visits made by those who have already visited at least once. Estimations were made from data available for under-3-y-old children from the 3 core villages. We estimated that 466 children per group would provide 80% power to detect a 15% reduction in repeat clinic visits with intervention, and 252 would detect a 20% difference. We aimed to recruit 500 children per group.

All participants, who received at least 1 dose of SQ-LNS were included in the analysis using STATA (version 12) based on a predefined analysis plan agreed with the trial monitor (S4 Text). Children who were wrongly recruited and who did not receive any supplement were not included in the analysis. For repeat clinic presentations, we used a negative binomial model relating the number of clinic visits to treatment group and known predictors of clinic visits. We included variables to reduce noise rather than remove confounding. Variables included were treatment group, distance to the nearest health clinic, distance to the Keneba clinic, age, weight-for-height z-scores (WHZ) at recruitment, season at recruitment, sex, and access to transport to the clinic. The analysis of effects on growth (z-scores) referenced internal growth data collected by KEMReS. Growth was then analysed with a mixed model, capturing individual differences as random effects for both intercept and slope. Season, WHZ at recruitment, sex, and age were included in the regression analysis as known predictors of growth. Stunting was defined as height-for-age z-scores (HAZ) of less than −2 standard deviation (SD) based on the WHO growth reference data. [14] The statistical analysis was extended (deviation from the original statistical analysis plan) to assess repeat clinic presentations within the initial 3 wk of supplementation.

The trial was registered as ISRCTN 73571031 before the start of recruitment (http://www.controlled-trials.com/).

An independent data safety monitor and an independent trial monitor assessed the study protocol and suitability of the trial site, assessed interim data, and assessed all serious adverse events (SAE). They also approved the statistical analysis plan (S4 Text) prior to the blinded analysis. The trial was terminated at the end of the prespecified 30-mo recruitment period. This study is reported as per CONsolidated Standards of Reporting Trials (CONSORT) guidelines (S1 CONSORT Checklist).

## Results

**Fig 1:**
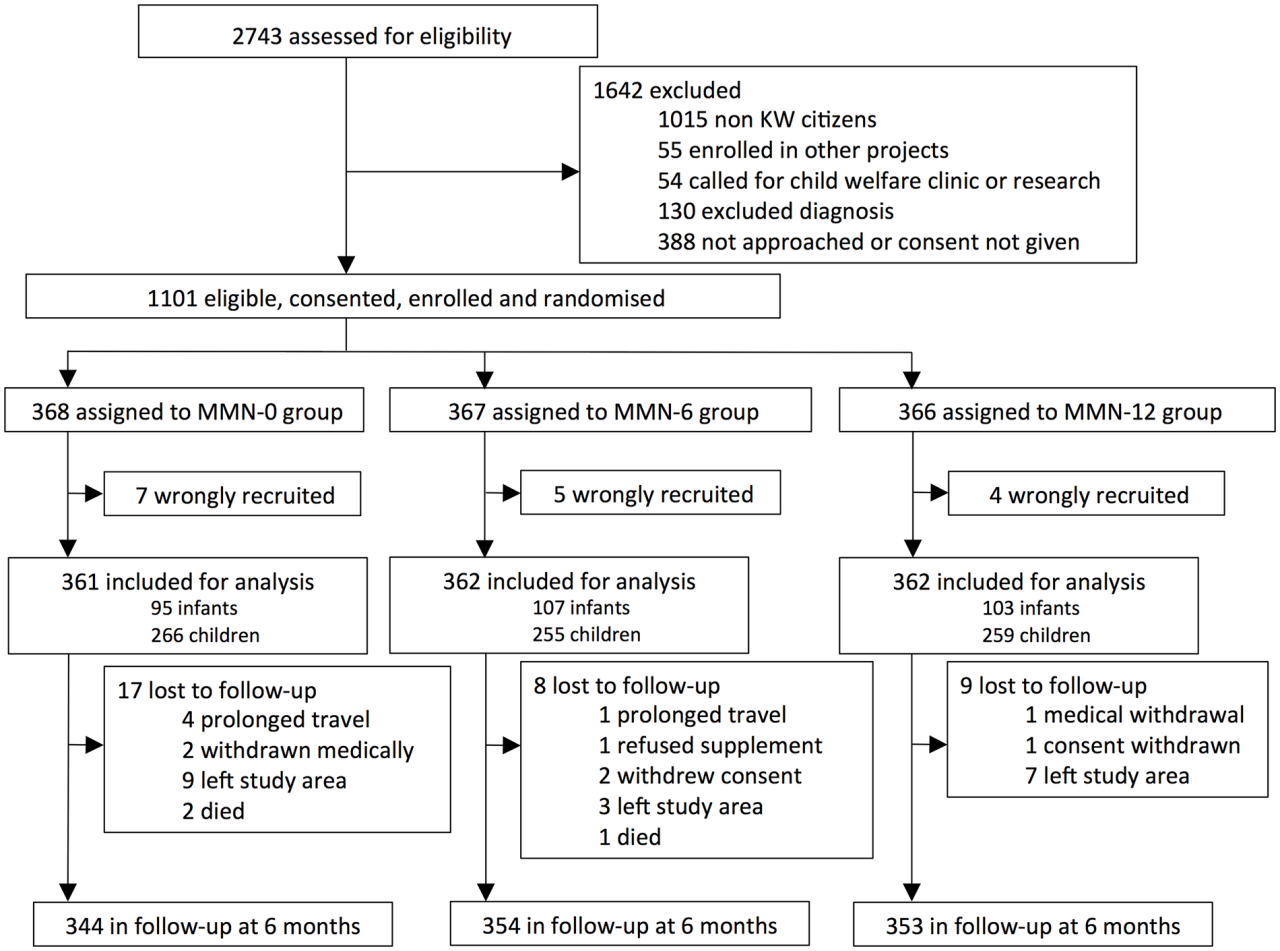
CONSORT flow chart of the trial. KW, Kiang West; MMN, multiple micronutrients.

Between 12 December 2009 and 3 June 2011, 2,743 children presenting to the primary care clinic were assessed for eligibility, of whom 1,642 children were either not eligible, the research clinician was unable to perform trial assessment, or the caregiver did not give consent. The remaining 1,101 children entered the trial (Fig 1). Sixteen children were recruited but did not fulfil the inclusion criteria and hence were withdrawn and removed from the analysis. After 12 wk, 1,051 (96.8%) children remained under follow-up and 1,020 (94.0%) after 24 wk.

The 3 groups were similar at baseline for a range of indicators including distance to the health facility and crowding (Table 1). Disease patterns at initial presentation were similar across treatment groups (Table 1).

**Table 1:** Baseline characteristics according to randomised groups.

	MMN-0 (*N* = 361)	MMN-6 (*N* = 362)	MMN-12 (*N* = 362)
**Socio-demographic factors**			
Mean age in y	2.1 (1.3)	2.1 (1.3)	2.1 (1.3)
Male	54% (193/361)	55% (198/362)	50% (180/362)
Mandinka ethnicity	84% (303/361)	86% (312/362)	85% (307/362)
Mean distance to Keneba clinic (km)	10.1 (8.3)	9.4 (7.9)	9.9 (8.2)
Mean distance to closest health centre (km)	7.4 (5.6)	7.1 (5.5)	7.3 (5.6)
Home village off a main road	14% (50/361)	12% (45/362)	11% (38/362)
Dead sibling in the family	24% (86/361)	25% (90/362)	22% (79/362)
Mean number of paternal siblings	7.5 (5.8) (*n* = 334)	7.0 (5.9) (*n* = 339)	7.3 (6.0) (*n* = 336)
Mean number of maternal siblings	4.1 (2.6) (*n* = 360)	4.0 (2.8) (*n* = 359)	4.1 (2.5) (*n* = 355)
Mean maternal birth order	4.5 (2.6) (*n* = 360)	4.4 (2.8) (*n* = 359)	4.4 (2.4) (*n* = 355)
Mean number of co-wives	1.7 (0.9) (*n* = 328)	1.7 (0.9) (*n* = 331)	1.7 (0.9) (*n* = 326)
Mean mother’s age	31.0 (6.8) (*n* = 357)	31.2 (7.4) (*n* = 354)	31.5 (7.2) (*n* = 354)
Mothers without education in English school	84% (302/360)	86% (310/362)	83% (300/361)
Mother dead	0.8% (3/361)	0.3% (1/362)	0.6% (2/362)
Child not living with mother	21% (75/361)	16% (57/362)	18% (65/362)
Parents divorced	11% (39/361)	10% (36/362)	13% (46/362)
**Presenting disease diagnoses at recruitment**			
Total number of diagnoses given	461	449	476
Infectious disease diagnosis*	91% (419/461)	92% (414/449)	91% (431/476)
Respiratory infection diagnosis	42% (193/461)	49% (220/449)	46% (218/476)
Diarrhoeal infection diagnosis	24% (112/461)	21% (94/449)	22% (103/476)
Malaria	0.2% (1/461)	0.5% (2/449)	0.4% (2/476)
Skin infection	17% (78/461)	13% (56/449)	13% (62/476)
Other infection	8% (35/461)	9% (42/449)	10% (46/476)
Injury	1% (5/461)	2% (7/449)	0·4% (2/476)
Nutrition related*	0.2% (1/461)	1% (5/449)	1% (1/476)
Other diagnoses	8% (36/461)	5% (23/449)	8% (39/476)

Data are *N*, % (*n*/*N*) or mean (SD). The number of participants with data available is given in brackets if less than the participant number per intervention group.

*Definition in statistical analysis in appendix. MMN, multiple micronutrients; MMN-0, supplementation with unfortified SQ-LNS for 12 wk; MMN-6, supplementation with micronutrient-fortified SQ-LNS for 6 wk followed by unfortified SQ-LNS for 6 wk; MMN-12, supplementation with micronutrient-fortified SQ-LNS for 12 wk.

Overall mean compliance when including those lost to follow-up or withdrawn was 85% (87% excluding those lost to follow-up) and was similar across intervention arms (Table 2). We did not observe any reduction in consumption over time and sharing of supplement as reported by the caregiver was minimal (<1%).

**Table 2:** Compliance overall and by intervention group.

		Overall	MMN-0 group	MMN-6 group	MMN-12 group	*p* value*
Number of participants at recruitment		1,101	368	367	366	
Number of participants after exclusions of those wrongly recruited (entered into analysis)		1,085	361	362	362	
**Mean percentage of compliance including those lost to withdrawal/lost to follow-up** (SD, median)		85% (16%)	85% (16%)	86% (14%)	85% (17%)	0.299
**Number of withdrawals/lost to follow-up during supplementation**		34	17	8	9	0.107
Details of withdrawals/lost to follow-ups	Travel	5	4	1	0	
	Medical	3	2	0	1	
	Refusal	1	0	1	0	
	Consent withdrawn	3	0	2	1	
	Migration	19	9	3	7	
	Died	3	2	1	0	
Number of participants after excluding those lost to follow-up		1,051	344	354	353	
**Mean percentage of compliance excluding those lost to withdrawal/lost to follow-up** (SD, median)		87% (12%)	87% (11%)	87% (12%)	87% (13%)	0.968
Number of noncompliant participants		102/1,051 (10%)	31/344 (9%)	31/354 (9%)	40/353 (11%)	0.445
Number of follow-ups with no information due to travel of the participant		110/6,103 (2%)	30/1,992 (2%)	39/2,059 (2%)	41/2,052 (2%)	0.465
Number of incomplete forms		16/5,993 (0.3%)	4/1,962 (0.2%)	6/2,020 (0.3%)	6/2,011 (0.3%)	0.804
Number of follow-ups indicating vomiting with supplementation		13/5,977 (0.2%)	6/1,958 (0.3%)	4/2,014 (0.2%)	3/2,005 (0.2%)	0.556
Number of follow-ups indicating refusal of supplement		96/5,977 (1.6%)	28/1,958 (1.4%)	33/2,014 (1.6%)	35/2,005 (1.7%)	0.725
Number of follow-ups indicating sharing of supplement		7/5,977 (0.1%)	2/1,958 (0.1%)	1/2,014 (0.1%)	4/2,005 (0.2%)	0.411

* *p* values shown here are derived from ANOVA or Pearson’s chi-squared analysis comparing the 3 intervention groups. MMN, multiple micronutrients; MMN-0, supplementation with unfortified SQ-LNS for 12 wk; MMN-6, supplementation with micronutrient-fortified SQ-LNS for 6 wk followed by unfortified SQ-LNS for 6 wk; MMN-12, supplementation with micronutrient-fortified SQ-LNS for 12 wk.

### Repeat clinic visits

Overall, there were 2,514 return clinic visits within 24 wk of recruitment. Of those, 2,224 visits (89%) were included in subsequent analysis according to the statistical analysis plan (S4 Text). Median re-presentation was 2 (IQR 0–4, range 0–15). A quarter of the children never presented again to a clinic within Kiang West during the 24-wk follow-up period. There was no significant effect of supplementation on repeat visits overall (adjusted incidence rate ratio [IRR] 1.04 [95% CI: 0.94, 1.15]) and no difference between the 2 fortified SQ-LNS groups (Table 3). Similarly, there was no significant effect on re-presentation when separated into respiratory, diarrhoeal, and malarial disease, and skin infections or when clinic visits with severe illness were analysed separately.

**Table 3:** Incidence of all clinic presentations and those with a respiratory and diarrhoeal diagnosis by intervention group and period after recruitment.

	MMN-0 group (*N* = 361)		MMN groups combined (*N* = 724)					MMN-6 group (*N* = 362)					MMN-12 group (*N* = 362)				
	Incidence per child wk	IRR	Incidence per child wk	IRR (unadjusted)	*p* value	IRR* (adjusted)	*p* value	Incidence per child wk	IRR (unadjusted)	*p* value	IRR* (adjusted)	*p* value	Incidence per child wk	IRR (unadjusted)	*p* value	IRR* (adjusted)	*p* value
**24 wk follow-up**																	
All clinic presentations	0.09 (740)	1.0	0.09 (1,571)	1.07 (0.95–1.21)	0.29	1.04 (0.94–1.15)	0.45	0.09 (789)	1.07 (0.94–1.24)	0.29	1.03 (0.92–1.16)	0.60	0.09 (782)	1.06 (0.92–1.22)	0.44	1.05 (0.93–1.18)	0.43
Respiratory	0.04 (380)	1.0	0.05 (851)	1.13 (0.97–1.31)	0.13	1.10 (0.96–1.26)	0.18	0.05 (438)	1.17 (0.98–1.39)	0.086	1.12 (0.95–1.31)	0.17	0.05 (413)	1.09 (0.91–1.30)	0.36	1.08 (0.92–1·27)	0.34
Diarrhoeal	0.02 (201)	1.0	0.02 (412)	1.03 (0.85–1.25)	0.75	1.02 (0.85–1.22)	0.81	0.02 (198)	1.00 (0.80–1.24)	0.97	0.96 (0.78–1.19)	0.73	0.03 (214)	1.07 (0.50–0.65)	0.86	1.08 (0.88–1.33)	0.45
**3 wk follow-up**																	
All clinic presentations	0.06 (60)	1.0	0.08 (177)	1.45 (1.08–2.00)	0.015	1.43 (1.07–1.92)	0.016										
Respiratory	0.03 (31)	1.0	0.05 (98)	1.55 (1.02–2.36)	0.039	1.52 (1.01–2.30)	0.046										
Diarrhoeal	0.02 (16)	1.0	0.006 (49)	1.50 (0.86–2.64)	0.157	1.54 (0.87–2.72)	0.14										
**6 wk follow-up**																	
All clinic presentations	0.09 (190)	1.0	0.11 (459)	1.20 (0.99–1.44)	0.056	1.17 (0.99–1.39)	0.063										
Respiratory	0.05 (100)	1.0	0.06 (250)	1.24 (0.97–1.58)	0.088	1.21 (0.96–1.53)	0.10										
Diarrhoeal	0.03 (56)	1.0	0.03 (135)	1.19 (0.87–1.63)	0.27	1.20 (0.88–1.64)	0.25										
**12 wk follow-up**																	
All clinic presentations	0.09 (367)	1.0	0.10 (852)	1.16 (1.00–1.33)	0.049	1.14 (0.99–1.29)	0.057	0.10 (433)	1.19 (1.01–1.40)	0.044	1.15 (0.99–1.33)	0.071	0.10 (419)	1.13) 0.96–1.33)	0.16	1.12 (0.97–1.31)	0.128
Respiratory	0.05 (193)	1.0	0.05 (460)	1.19 (0.99–1.43)	0.067	1.16 (0.97–1.38)	0.10	0.06 (242)	1.26 (1.02–1.55)	0.029	1.22 (0.99–1.48)	0.050	0.05 (218)	1.12 (0.90–1.38)	0.31	1.10 (0.90–1.34)	0.37
Diarrhoeal	0.03 (104)	1.0	0.03 (238)	1.14 (0.89–1.46)	0.296	1.16 (0.91–1.47)	0.224	0.03 (117)	1.13 (0.85–1.50)	0.39	1.13 (0.86–1.48)	0.40	0.03 (121)	1.15 (0.87–1.53)	0.33	1.19 (0.91–1.57)	0.20

Data are incidence per child wk (total number of episodes) or IRR (95% CI).

*IRRs, CIs, and *p* values are adjusted for age, sex, season, location (access to transport and distance), and weight-for-height z-scores at recruitment. IRR, incidence rate ratio; MMN, multiple micronutrient; MMN-0, supplementation with unfortified SQ-LNS for 12 wk; MMN-6, supplementation with micronutrient-fortified SQ-LNS for 6 wk followed by unfortified SQ-LNS for 6 wk; MMN-12, supplementation with micronutrient-fortified SQ-LNS for 12 wk; wk, week.

During post hoc analysis, it became apparent that the effect of MMN on re-presentations became significant when only looking at the initial period after recruitment (initial 3 wk) (Table 3). MMN supplementation significantly increased, rather than decreased, clinic return presentations (adjusted IRR 1.43; 95% CI: 1.07, 1.92; *p* = 0.016; risk difference = 8/100). Respiratory infections were increased by 52%, although the effect was only borderline significant (adjusted IRR 1.52; 95% CI: 1.01, 2.30; *p* = 0.046, risk difference = 5/100). There was no significant increase in severe presentation seen within the first 3 wk (adjusted IRR 1.80; 95% CI: 0.58, 1.60; *p* = 0.307). Severe adverse events overall were also not different between intervention groups (S2 Table).

### Growth

Stunting was prevalent in 25.4% (276/1,085) of our study population at baseline. Similarly to usual trends among infants and young children in this population, HAZ declined across all intervention arms over the 6-mo follow-up (Table 4). However, the decrease was less marked in the MMN-12 group, with a benefit of 0.084 standard deviations (95% CI: 0.005, 0.168) at 24 wk (Table 4). There was no evidence at 24 wk, however, that MMN-12 out-performed MMN-6 (95% CI: −0.118, 0.084). No statistically significant effects on z-scores for weight, MUAC, or skin-fold thickness were detected. Anthropometric measures were statistically significantly affected by season, and all analyses were adjusted for season as specified in the statistical analysis plan. (S4 Text).

**Table 4:** Anthropometric measurements at recruitment and follow-up dates by intervention group.

	MMN-0 group		MMN groups combined		MMN-6 group		MMN-12 group		*p* value (unadjusted)^$^	*p* value* (adjusted)
	Mean (SD)	Difference to recruitment (SD)	Mean (SD)	Difference to recruitment (SD)	Mean (SD)	Difference to recruitment (SD)	Mean (SD)	Difference to recruitment (SD)		
**Recruitment**	***n* = 361**				***n* = 362**		***n* = 362**			
HAZ	−1.38 (1.05)		−1.34 (1·11)		−1.37 (1.11)		−1.32 (1.10)			
WHZ	−0.78 (1.14)		−0.63 (1.12)		−0.75 (1.03)		−0.52 (1.03)			
WAZ	−1.35 (1.04)		−1.24 (1.08)		−1.33 (1.11)		−1.16 (1.04)			
MAZ	−0.94 (0.96)		−0.79 (1.00)		−0.85 (0.99)		−0.73 (0.99)			
Stunting (%)^§^	23.0% (83/361)		26.7% (193/724)		27.6% (100/362)		25.7% (93/362)			
**6 wk**	***n* = 344**				***n* = 351**		***n* = 347**			
HAZ	−1.42 (1.07)	−0.04 (0.61)	−1.41 (1.07)	−0.06 (0.52)	−1.43 (1.12)	−0.06 (0.59)	−1.39 (1.09)	−0.07 (0.45)		
WHZ	−0.60 (1.00)	0.17 (0·82)	−0.44 (1.00)	0.20 (0.76)	−0.52 (1.05)	0.24 (0.80)	−0.36 (0.94)	0.17 (0.72)		
WAZ	−1.18 (1.03)	0.11 (0.59)	−1.15 (1.02)	0.14 (0.53)	−1.18 (0.95)	0.15 (0.54)	−1.04 (0.95)	0.14 (0.52)		
MAZ	−0.78 (0.90)	0.17 (0.67)	−0.62 (0.94)	0.18 (0.67)	−0.70 (0.95)	0.15 (0.67)	−0.53 (0.92)	0.21 (0.67)		
Stunting (%)^§^	27.6% (95/344)		28.7% (202/698)		29.1% (102/351)		28.8% (100/347)			
**12 wk**	***n* = 341**				***n* = 351**		***n* = 346**			
HAZ	−1.46 (1.02)	−0.07 (0.62)	−1.40 (1.11)	−0.05 (0.57)	−1.45 (1.15)	−0.07 (0.63)	−1.35 (1.08)	−0.03 (0.49)		
WHZ	−0.65 (1.01)	0.14 (0.89)	−0.52 (1.00)	0.17 (0.79)	−0.52 (1.00)	0.23 (0.81)	−0.39 (0.96)	0.11 (0.76)		
WAZ	−1.29 (1.04)	0.07 (0.60)	−1.14 (1.00)	0.11 (0.56)	−1.21 (1.03)	0.13 (0.53)	−1.06 (0.96)	0.09 (0.58)		
MAZ	−0.86 (0.96)	0.11 (0.78)	−0.66 (0.95)	0.13 (0.72)	−0.75 (0.99)	0.11 (0.74)	−0.57 (0.91)	0.14 (0.71)		
Stunting (%)^§^	26.1% (89/341)		29.0% (202/697)		31.1% (109/351)		26.9% (93/346)			
**24 wk**	***n* = 336**				***n* = 343**		***n* = 341**			
HAZ	−1.57 (1.08)	−0.19 (0.85)	−1.46 (1.09)	−0.10 (0.74)	−1.50 (1.16)	−0.12 (0.85)	−1.41 (1.01)	−0.09 (0.61)	0.480	0.037**
WHZ	−0.65 (1.12)	0.15 (1.14)	−0.57 (1.02)	0.10 (0.89)	−0.59 (1.02)	0.17 (0.91)	−0.48 (1.01)	0.02 (0.87)	0.144	0.429
WAZ	−1.34 (1.00)	−0.01 (0.73)	−1.21 (1.01)	0.05 (0.68)	−1.27 (1.04)	0.07 (0.69)	−1.15 (0.97)	0.03 (0.68)	0.088	0.229
MAZ	−0.81 (0.99)	0.11 (0.87)	−0.71 (0.97)	0.12 (0.81)	−0.73 (0.94)	0.11 (0.74)	−0.60 (0.96)	0.15 (0.81)	0.057	0.282
Stunting (%)^§^	29.5% (99/336)		28.1% (192/684)		30.0% (103/343)		26.1% (89/341)			
**2 years**	***n* = 339**				***n* = 351**		***n* = 351**			
HAZ	−1.12 (1.42)	0.27 (1.41)	−1.12 (1.25)	0.23 (1.10)	−1.18 (1.29)	0.20 (1.03)	−1.05 (1.30)	0.26 (1.17)	0.456	0.872
WHZ	−0.68 (0.93)	0.15 (1.11)	−0.34 (0.95)	0.08 (1.03)	−0.65 (0.98)	0.19 (1.11)	−0.63 (0.93)	−0.05 (0.93)	0.130	0.344
WAZ	−1.31 (0.89)	0.03 (0.82)	−1.23 (0.93)	0.03 (0.78)	−1.30 (0.93)	0.05 (0.76)	−1.17 (0.93)	0.005 (0.80)	0.031	0.533
MAZ	−0.68 (0.84)	0.25 (1.09)	−0.59 (0.90)	0.15 (1.03)	−0.58 (0.99)	0.20 (0.12)	−0.61 (0.85)	0.11 (0.94)	0.052	0.432
Stunting (%)^§^	16.8% (57/339)		17.8% (125/702)		18.5% (65/351)		17.1% (60/651)			

Data are anthropometric z-scores (SD). All z-scores were calculated based on the WHO 2006 anthropometric dataset.

^§^ Proportion stunted expressed as % (*n*) * *p* values shown here are derived from mixed regression models looking at the effect of intervention overall (MMN combined). Mixed regression models are adjusted for age, sex, season, and WHZ at recruitment.

**Effect size is 0.0005 (95% CI 0.00003, 0.0010) z-score per day, equivalent to 0.084 (95% CI: 0.00003, 0.0010) z-score change over 24 wk.

^$^ *p* values shown here are derived from a simple one-way ANOVA of differences between measurement at 24 or 2 years (y) and recruitment. HAZ, height-for-age z score; MAZ, mid-upper arm circumference (MUAC)-for-age z score; MMN, multiple micronutrient; MMN-0, supplementation with unfortified SQ-LNS for 12 wk; MMN-6, supplementation with micronutrient-fortified SQ-LNS for 6 wk followed by unfortified SQ-LNS for 6 wk; MMN-12, supplementation with micronutrient-fortified SQ-LNS for 12 wk; WAZ, weight-for-age z score; WHZ, weight-for-height z-score.

### Micronutrient status

Table 5 shows the means of haemoglobin and plasma retinol, 25(OH)D, selenium, and zinc levels and effect sizes after 12 wk of supplementation. Mean haemoglobin concentrations were 0.53 g/dl lower in the unfortified SQ-LNS group compared to both fortified groups combined with the regression analysis showing an adjusted effect size of MMN supplementation of 0.44 g/dl (95% CI: 0.03, 0.85; *p* = 0.037). There was no difference between the MMN-6 and MMN12 groups (−0.02; 95% CI: −0.02, 0.93; *p* = 0.923). The proportion of anaemic children (Hb < 11.0 g/dL) was lower in the MMN groups (MMN-0 46%, MMN-6 36%, MMN-12 33%).

**Table 5:** Haematological outcomes after 12 wk of supplementation.

	MMN-0 group	MMN-6 group	MMN-12 group	MMN combined Effect size	*p* value* (unadjusted)	*p* value** (adjusted)
**Haemoglobin (g/dl)**						
*N*	59	69	60			
Mean	11.06 (10.75–11.37)	11.58 (11.25–11.90)	11.48 (11.17–11.81)	0.44 g/dl/12 wk (95% CI 0.03, 0.85)	0.018	0.034
% anaemic (Hb < 11 g/dl)	46% (33%–59%)	36% (25%–48%)	33% (21%–45%)			
% anaemic (Hb < 10 g/dl)	14% (7%–25%)	7% (3%–16%)	8% (4%–19%)			
**Retinol (μmol/l)**						
*N*	61	72	61			
Mean	0.91 (0.84–0.98)	0.92 (0.85–0.98)	0.92 (0.86–0.98)	0.05 μmol/l/12 wk (95% CI −0.03, 0.12)	0.91	0.231
% deficiency (Retinol < 0.7 μmol/l)	18% (8%–28%)	26% (16%–37%)	20% (10%–30%)			
**25(OH) Vitamin D (nmol/l)**						
*N*	61	73	61			
Mean	66.25 (61.25–71.25)	71.92 (67.34–76.51)	79.90 (73.56–86.24)	12%/12 wk (95% CI 3, 22)	0.003	0.011^§^
% mild deficiency (<75 nmol/l)	77% (67%–88%)	66% (55%–77%)	49% (37%–62%)			
% moderate deficiency (<50 nmol/l)	16% (6%–23%)	14% (6%–22%)	6% (1%–13%)			
**Zinc (μg/l)**						
*N*	62	73	60			
Mean	727.59 (694.46–760.72)	724.56 (692.53–756.59)	768.86 (713.06–824.67)	3%/12 wk (95% CI −3, 10)	0.50	0.31
% deficiency (<647 μg/l)	27% (16%–39%)	30% (20%–41%)	33% (21%–45%)			
**Selenium (μg/l)**						
*N*	62	73	60			
Mean	100.16 (94.08–106.23)	97.52 (92.51–102.53)	97.73 (93.02–102.46)	−2%/12 wk (95% CI −9, 5)	0.44	0.63
Percentage deficiency (≤79 μg/l)	13% (4%–21%)	15% (7%–23%)	13% (5%–22%)			
**CRP (mg/l)**						
*N*	54	68	56			
Mean	4.2 (2.8–5.6)	5.6 (3.4–7.9)	7.8 (1.8–13.8)			
**AGP (g/l)**						
*N*	54	68	56			
Mean	0.99 (0.89–1.08)	0.93 (0.85–1.01)	0.94 (0.86–1.02)			

Data are means or proportions (95% CI).

*unadjusted *p* values for regression analysis of effect of MMN overall (both treatment groups combined).

**p values for regression analysis of effect of MMN overall (both treatment groups combined) adjusted for age, sex, season, weight-for-height z-scores at recruitment, CRP, and AGP.

^§^The difference between the 6- and 12-wk MMN group was also statistically significantly different, *p* = 0.027. AGP, alpha(1)-acid glycoprotein; CRP, C-reactive protein; MMN, multiple micronutrient; MMN-0, supplementation with unfortified SQ-LNS for 12 wk; MMN-6, supplementation with micronutrient-fortified SQ-LNS for 6 wk followed by unfortified SQ-LNS for 6 wk; MMN-12, supplementation with micronutrient-fortified SQ-LNS for 12 wk; wk, week.

Adjusted regression analysis showed a 12% (95% CI: 3%, 22%; *p* = 0.011) change in plasma 25(OH)D levels with MMN supplementation relative to unfortified SQ-LNS. The effect was dose-dependent with a significant difference between MMN-6 and MMN-12 (*p* = 0.027). Overall, 30% (59/195) of the children tested were zinc deficient (plasma zinc <647μg/l), 22% (42/194) had mild vitamin A deficiency (plasma retinol <0.70 μmol/l), and 14% (27/195) had plasma selenium levels below the recommended level for maximal activity of glutathione peroxidase and selenoprotein P (plasma selenium concentration >1.2 μmol). MMN supplementation did detectably not improve the status of plasma zinc, selenium, and retinol among study participants (Table 5).

### Actively-surveyed morbidity

**Fig 2:**
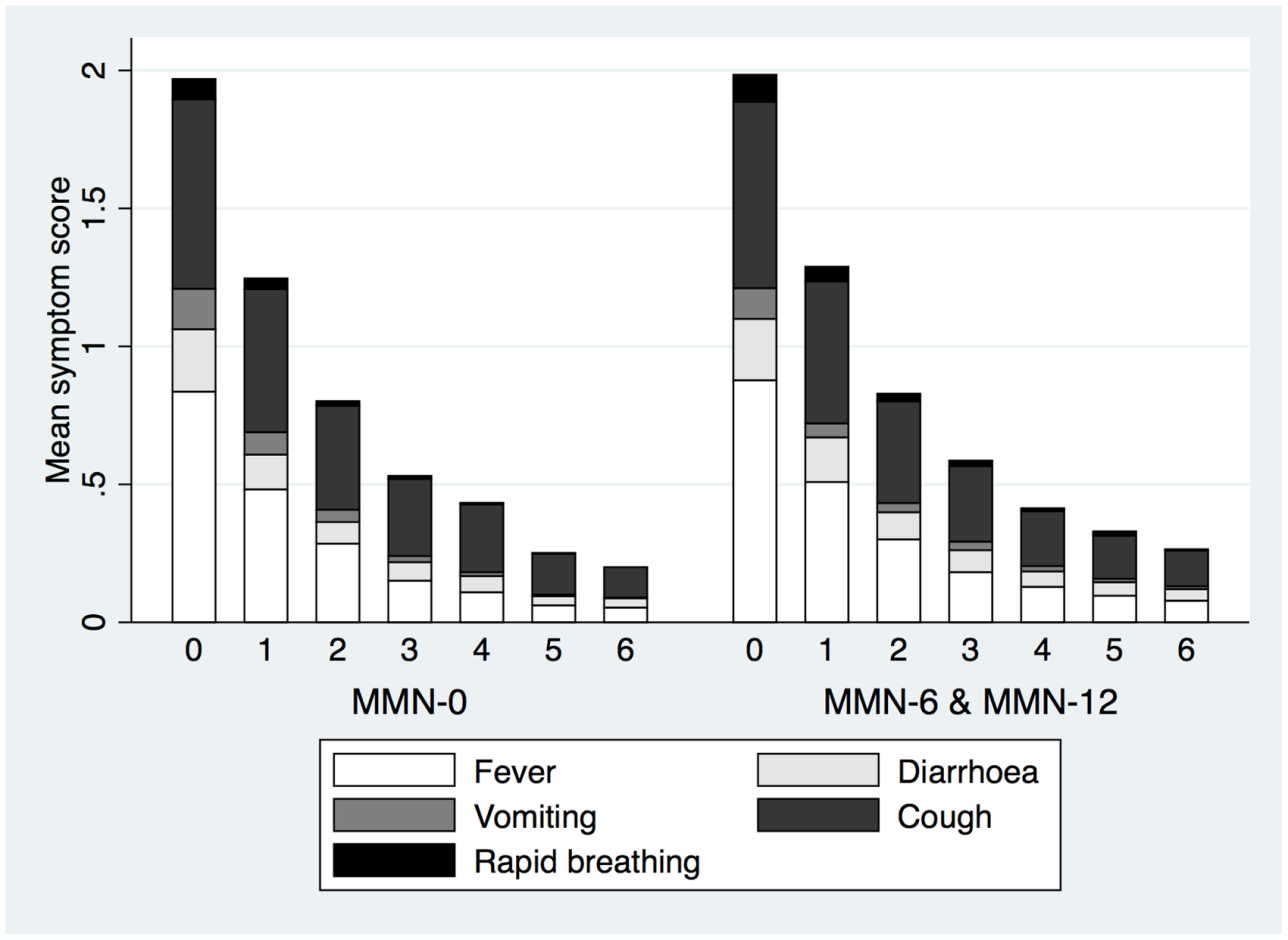
Means of symptom scores and proportion of types of symptoms reported at recruitment and over the following 6 days.

Reported morbidity (daily over the first 7 days postrecruitment and fortnightly thereafter until the end of supplementation) showed that symptom scores and symptom resolution over the first wk of supplementation was not different across intervention groups (Table 6, Fig 2). Similarly, symptom scores overall and separated for respiratory and diarrhoeal symptoms were not statistically different during the supplementation period (Table 6).

**Table 6:** Effect on reported morbidity.

	MMN-0 group	MMN groups combined		*p* value	Regression coefficient (95% CI)**		MMN-6 group		*p* value	Regression coefficient (95% CI)**	*p* value	MMN-12 group		*p* value	Regression coefficient (95% CI)**	*p* value
	Mean symptom score (SD)	Mean symptom score (SD)	Regression coefficient (95% CI)**				Mean symptom score (SD)	Regression coefficient (95% CI)**				Mean symptom score (SD)	Regression coefficient (95% CI)**			
12 wk follow-up*																
Observations (*n*)	*n* = 2065						*n* = 2079					*n* = 2087				
Overall symptom score	0.37 (0.80)						0.39 (0.81)	0.11 (−0.08, 0.30)	0.27	0.11 (−0.08, 0.29)	0.27	0.36 (0.77)	−0.01 (−0.20, 0.19)	0.94	0.02 (−0.18, 0.21)	0.86
Respiratory	0.21 (0.57)						0.23 (0.59)	0.10 (−0.13, 0.32)	0.42	0.09 (−0.14, 0.31)	0.45	0.21 (0.56)	0.01 (−0.21, 0.24)	0.92	0.03 (−0.20, 0.26)	0.82
Diarrhoeal	0.12 (0.43)						0.11 (0.42)	−0.04 (−0.33, 0.25)	0.80	−0.05 (−0.34, 0.23)	0.73	0.10 (0.42)	−0.14 (−0.44, 0.17)	0.38	−0.12 (−0.44, 0.20)	0.47
**1 wk follow-up**																
Observations (*n*)	*n* = 2046	*n* = 4075														
Overall symptom score	0.78 (1.02)	0.81 (1.03)	0.07 (−0.07, 0.22)	0.32	0.10 (−0.05, 0.24)	0.18										
Respiratory	0.76 (0.86)	0.76 (0.87)	−0.03 (−0.21, 0.16)	0.78	−0.01 (v0.20, 0.17)	0.89										
Diarrhoeal	0.54 (0.82)	0.59 (0.85)	0.05 (−0.32, 0.41)	0.80	0.04 (−0.32, 0.40)	0.84										
6 wk follow-up*																
Observations (*n*)	*n* = 1031	*n* = 2057														
Overall symptom score	0.34 (0.75)	0.39 (0.80)	0.01 (−0.04, 0.37)	0.12	0.18 (−0.03, 0.39)	0.10										
Respiratory	0.19 (0.55)	0.23 (0.59)	0.18 (−0.08, 0.43)	0.18	0.18 (−0.08, 0.44)	0.17										
Diarrhoeal	0.10 (0.40)	0.11 (0.42)	0.04 (−0.28, 0.37)	0.80	0.07 (−0.26, 0.40)	0.70										

Data are symptom scores (SD) or regression coefficient (95% CI).

*The 6- and 12-wk morbidity follow-up data does not include week (wk) 1 daily morbidity observations.

**Regression coefficients, CIs, and *p* values are adjusted for age, sex, season, and weight-for-height z-scores at recruitment. MMN, multiple micronutrient; MMN-0, supplementation with unfortified SQ-LNS for 12 wk; MMN-6, supplementation with micronutrient-fortified SQ-LNS for 6 wk followed by unfortified SQ-LNS for 6 wk; MMN-12, supplementation with micronutrient-fortified SQ-LNS for 12 wk.

### Appetite

Appetite was recorded daily for the first week after recruitment and fortnightly thereafter until the end of active intervention at 12 wk. Supplemented participants were 8% more likely to show an improved appetite within the first week (hazard ratio: 1.08; 95% CI: 1.01, 1.15; *p* = 0.018) (S2 Table). MMN supplementation also significantly improved appetite scores overall during the 12-wk supplementation period (0.29 score/12 wk; 95% CI: 0.07, 0.53; *p* = 0.010). The effect was even more pronounced during the first 6 wk after enrolment (0.40 score/6 wk; 95% CI: 0.12, 0.67; *p* = 0.005).

## Discussion

To our knowledge, this is the first randomised controlled trial to investigate the effects of micronutrient-fortified versus unfortified SQ-LNS in children presenting to a primary healthcare facility. We studied a well-described population with a high rate of stunting, little migration, and low loss to follow-up. Reported compliance was probably the best that can be achieved when supplementation is not directly observed [16] and exceeded what would occur in programmatic settings.

There was no reduction in repeat clinic attendances and very minimal benefit to growth despite an enhancement of appetite with added MMN. The suggestion, upon secondary analysis, that morbidity was elevated by micronutrients is potentially concerning.

Point of care provision of SQ-LNS-MMN compared to unfortified SQ-LNS did not reduce return clinic presentations within a 6-mo period despite the high compliance. Neither was there an effect on the duration of the clinical symptoms associated with the initial presentation. However, appetite improved appreciably with MMN. In the primary analysis, there was a nonsignificant trend to increased morbidity over the entire 6-mo follow-up. The incidence of severe illness during follow-up did not differ between the groups, although this was based on few events. However, post hoc analysis revealed a 43% increase in total return visits to clinic (95% CI 7%–92%) within the first 3 wk of intervention in the 2 SQ-LNS-MMN groups. There are several grounds to suspect that the 12 mg per day iron in the MMN could be responsible for this effect. [17] Iron lies at the centre of a host–pathogen battle for nutritional resource [18,19], and there are numerous instances in which iron is implicated as causing iatrogenic disease, including malaria, diarrhoea, and pneumonia. [20–22] Iron supplementation modifies the gut flora by enhancing potentially pathogenic organisms in the place of beneficial *lactobacilli* and *bifidobacteria*, and this increases inflammation in the gut. [20] These findings pose several challenges as to how best to combat iron deficiency in environmentally-challenged populations and imply that infection control must be prioritised.

There is an extensive, though heterogeneous, evidence base on the effects of single micronutrient interventions (mainly vitamins A and D, zinc, and iron) on morbidity outcomes, but the effect of MMN supplementation on child mortality and morbidity is underresearched. [22] Morbidity is often a secondary outcome measure [9,10], and data from previous MMN supplementation or fortification trials have shown variable outcomes in terms of their effect on morbidity, some suggesting improvements, [4] although many showing no detectable benefit on illness [9,22,23] despite showing improvement in iron status and anaemia. [21] Some previous studies have also suggested possible increases in certain morbidity outcomes, even with short-term supplementation. [10,21,24]

Receiving SQ-LNS with MMN had a very small benefit on growth. It reduced the typical decline in linear growth seen in this population by 0.084 z-score compared to unfortified SQ-LNS—equivalent to 2–5 mm depending on the age of the children with debatable clinical relevance. Twelve-week supplementation did not show significant advantage over a 6-wk supplementation period. The duration of supplementation was shorter than other SQ-LNS studies [4,6] although similar to some MMN supplementation trials. [25] Shorter courses of supplementation are associated with better compliance [26] and are more easily introduced in practice.

When we initiated our study, there was considerable optimism that lipid-based micronutrient supplements would have a significant impact on community-based nutritional intervention, and several trials coordinated by the International Lipid-Based Nutrient Supplements (iLiNS) consortium were in progress (www.ilins.org). [27] Some of these trials have now reported, with variable results. In Malawi, provision of SQ-LNS to mothers during pregnancy and for 6 mo postpartum and then to infants after 6 mo achieved no differential impact on growth by 18 mo. [6] In all groups, length-for-age deteriorated by approximately 0.4 z-score between 6 mo and 18 mo, and 35% of children were stunted at 18 mo. In a cluster-randomised trial in Burkino Faso, children receiving SQ-LNS with various doses of zinc (intervention cohorts) were 7 mm longer at 18 mo (equivalent to 0.25 SD), but the intervention groups also received weekly morbidity surveillance and treatment, which was not offered to the nonintervention cohorts. [4] The prevalence of stunting increased between 9 mo and 18 mo in both groups but significantly less so in the intervention cohorts (reaching 29% in intervention cohorts and 39% in the nonintervention cohorts, *p* < 0.001). A prior study in Ghana compared an earlier version of SQ-LNS (Nutributter) to crushable multiple-micronutrient tablets and encapsulated multiple-micronutrient powders (Sprinkles ©). [3] The baseline prevalence of stunting was low at 12%. Between 6 mo and 12 mo, the Nutributter significantly attenuated the decline in length-for-age by 0.18 z-score compared to the other 2 groups. A more recent supplementation trial in Ghana with SQ-LNS during pregnancy and for infants from 6 mo to 18 mo old led to a significantly greater length-for-age z-scores (LAZ) at 18 mo old compared with standard iron and folic acid (IFA) during pregnancy and placebo postpartum or MMN during pregnancy and lactation (LAZ: LNS −0.69; IFA −0.87, *p* = 0.006; MMN: −0.91, *p* = 0.009). [8] Similarly, a recent cluster-randomised effectiveness trial in Bangladesh providing LNS to women during pregnancy and 6 mo postpartum and to the infants from 6 mo to 24 mo old resulted in an increased LAZ (+0.13, *p* = 0.022) compared with providing IFA to mothers and MMN to children. [7] None of the SQ-LNS trials compared fortified versus nonfortified SQ-LNS.

Short-term supplementation with RUTF for 2 wk to nonmalnourished and moderately malnourished children in Nigeria during recovery from malaria, diarrhoea, or LRTI did not reduce the incidence of malnutrition over a 24-wk period compared to no supplement [10], whereas a 33.3% reduction was seen in nonmalnourished children in Uganda. [9] Discrepancy between the 2 studies may be related to the much lower incidence of malnutrition and infectious disease burden in Uganda. Micronutrient status was not assessed as part of either of the trials.

In common with most other MMN trials in low-income settings, we achieved a small but significant improvement in haemoglobin levels (+0.44 g/dl), but after 12 wk intervention, 33% of the children remained anemic (46% in the unfortified group). Some of the remaining anaemia might have been related to nonnutritional factors. It has also been suggested that for populations with African ancestry, lower thresholds for the diagnosis of anaemia may be more appropriate (Table 5 showing percentage of anaemic children for haemoglobin cutoff <11.0 g/dL and <10.0 g/dL). [28]

There was a measurable, dose-dependent improvement in vitamin D status (+12%) but not in vitamin A, zinc, or selenium status. Reasons for the lack in difference for vitamin A, zinc, and selenium may be related to a suboptimal amount contained in the supplement, poor absorption, homeostatic control mechanisms, and/or adequate levels in the study population (only 18%, 27%, and 13% were deficient in the unfortified group for vitamin A, zinc, or selenium, respectively) (Table 6).

We acknowledge several limitations when considering our results. Supplementation was not directly observed, and although recorded compliance was similar between groups and sharing was not reported, this cannot be guaranteed. The main outcome measure of morbidity, i.e., clinic re-presentation, is significantly influenced by distance from the home village to the health centre and families’ access to transport. [29] However, regression analyses were adjusted for distance and access to a main road. Morbidity recorded during supplementation was based on caregiver’s recall only. However, this was a randomised trial, and women were blinded to the intervention; subjectivity in morbidity reports should have influenced all groups equally but may have diluted our ability to detect an effect (negative or positive). Acceptance of the supplement in the population was good based on acceptability studies prior to the trial. The LNS paste may have replaced some of the daily intake in the younger age group hence diluting the effect of LNS combined with MMN. However, impact of SQ-LNS on feeding practices seems to be limited. [30, 31] The different micronutrient concentrations within the supplements for infants and children may have had a different impact on the outcomes measured; although, due to low numbers within each group, we do not have the power to confirm these suggestions (Fig 1). The number of participants recruited was lower than intended based on a priori power calculation (*n* = 1,101 versus *n* = 1,500). With the number of study participants enrolled, we would only detect a difference of 18% (compared to the initial 15% target) in the main outcome of clinic representations with 80% and 5% significance levels. Given that the secondary outcomes of reported morbidity and severe adverse events did not differ between intervention groups, the authors cannot rule out that the small difference in clinic re-presentations (when adjusted and using combined groups) may be a chance finding.

Our trial had many strengths. It was appropriately randomised and blinded, and loss to follow-up and missing data were minimal compared to other trials. In particular, the targeting of children presenting to a rural clinic had the potential to self-select a subpopulation at greatest need and to enhance recovery from the presenting condition. If it had proved effective, such a strategy would be more cost-effective and much easier to implement in terms of supply and distribution than population-wide mass administration.

We conclude that prescribing micronutrients via SQ-LNS to acutely ill nonmalnourished and mild-to-moderately malnourished children presenting at a primary care clinic in rural Gambia had only a minimal effect on growth compared to unfortified SQ-LNS. Micronutrient supplementation also did not reduce morbidity, and evidence for an early increase in repeat visits indicates that caution is warranted when prescribing MMN in a primary care setting, particularly if they include iron. It is highly likely that a combination of recurrent infection, persistent gastrointestinal enteropathy, and chronic inflammation are major factors in limiting the response to nutritional intervention.

## Supporting information

S1 CONSORT Checklist CONSORT checklist. (DOC)

S1 Text Trial protocol. (DOC)

S2 Text Ethical approval documents. (DOCX)

S3 Text Summary of literature review. (DOCX)

S4 Text Statistical analysis plan. (DOCX)

S5 Text Standard operating procedures for anthropometric measurements. (DOCX)

S1 Table Composition of small-quantity lipid-based multiple micronutrient supplement. (DOCX)

S2 Table Severe adverse events (SAE) by treatment group. (DOCX)

## Abbreviations

FW: fieldworker
HAZ: height-for-age z-scores
IFA: iron and folic acid
iLiNS: International Lipid-Based Nutrient Supplements Project
IRR: incidence rate ratio
KEMReS: Keneba Electronic Medical Records System
KW: Kiang West
KWDSS: Kiang West Demographic Surveillance System
LAZ: length-for-age z-scores
LNS: lipid-based nutrient supplements
MMN: multiple micronutrients
MMN-0: supplementation with unfortified SQ-LNS for 12 wk
MMN-6: supplementation with micronutrient-fortified SQ-LNS for 6 wk followed by unfortified SQ-LNS for 6 wk
MMN-12: supplementation with micronutrient-fortified SQ-LNS for 12 wk
mo: Months
MRC: Medical Research Council
MUAC: mid-upper arm circumference
RUTF: ready-to-use therapeutic foods
SQ-LNS: small-quantity lipid-based nutrient supplement
WHZ: weight-for-height z-scores
wk: week
y: year
