# Supplementary material for: Impact of fortified versus unfortified lipid-based supplements on morbidity and nutritional status: A randomised double-blind placebo-controlled trial in ill Gambian children
Source: PLoS Med. 2017 Aug 15;14(8):e1002377. doi: 10.1371/journal.pmed.1002377 (PMC5557358; doi:10.1371/journal.pmed.1002377)
Supplement: S1 Text — (DOC) [file pmed.1002377.s002.doc]

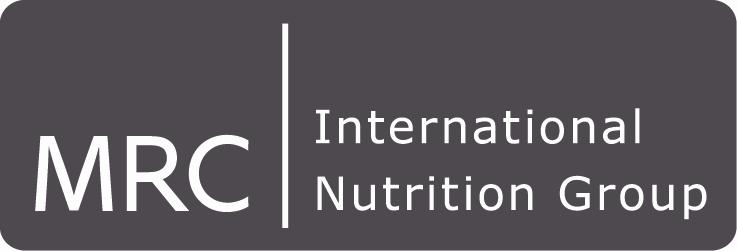


Lipid-based multiple micronutrient clinical trial in Gambian children. Protocol for a Randomized Controlled Trial

**[ISRCTN 73571031]**

**[MRC Laboratories, The Gambia: SCC 1135v2, Amendment L2009.58 & L2009.81]**

SCC1135v2

Scientific Coordinating Committee (SCC), MRC Laboratories, The Gambia; approved 7^th^ March 2009.

Gambian Government / MRC Ethics Committee; approved 27^th^ March 2009.

L2009.58

SCC, MRC Laboratories, The Gambia; approved 7^th^ September 2009.

Gambian Government/MRC Ethics Committee; approved 02^nd^ October 2009.

L2009.81

SCC, MRC Laboratories, The Gambia; approved 7^th^ December 2009.

Gambian Government/MRC Ethics Committee; approved 5^th^ January 2010.

Protocol prepared by

**Dr Stefan Unger**

Principle Investigator

# MRC Keneba Fieldstation

###### MRC Laboratories

*Fajara*

*PO Box 273*

*THE GAMBIA*

*sunger@mrc.gm*

*Telephone: 02209875929 or 02207104970*

**TRIAL SPONSOR**

MRC and the UK Department for International Development (DFID) under the MRC/DFID Concordat agreement (MC-A760-5QX00)

**COLLABORATIVE INVESTIGATORS**

**MRC Laboratories The Gambia, Keneba Fieldstation**

Dr. Sophie Moore

**MRC Laboratories The Gambia, Fajara**

Dr Stephen Howie

**National Nutritional Agency (NaNA), The Gambia**

Mr Modou Phall

**MRC International Nutrition Group, London School of Hygiene and Tropical Medicine**

Dr. Tony Fulford

Prof. Andrew Prentice

**TRIAL SUPERVISION**

**Independent Trial Monitor**

Prof. Kim Mulholland, Infectious Disease Epidemiology Unit, London School of Hygiene and Tropical Medicine.

**Data Safety Monitor**

Dr. Grant MacKenzie – MRC Laboratories, The Gambia

Abbreviations

AE Adverse Event

AI Adequate Intake

AN Audit Nurse

BP Blood Pressure

CRF Case Report Form

DRI Dietary Reference Intake

DSM Data and Safety Monitor

EPI Expanded Programme for Immunization

FW Fieldworker

FWSV Fieldworker Supervisor

HR Heart Rate

IMCI Integrated Management of Child Illness

IMP Investigational Medical Product

KWDS Kiang West Demographic Surveillance

LNS Lipid based nutritional supplement

LNS MMN Lipid based multiple micronutrients

LSHTM London School of Hygiene and Tropical Medicine

MMCT Multiple Micronutrient Clinical Trial

MMN Multiple Micronutrients

MRC Medical Research Council

MUAC Mid Upper Arm Circumference

O_2_ Oxygen

PI Principal Investigator

RCT Randomized controlled trial

RDA Recommended Daily Allowance

RHT Regional Health Team

RNI Recommended Nutrient Intake

RUTF Ready-to-Use-Therapeutic Foods

RR Respiratory Rate

SAE Serious Adverse Event

SDB Study Database

SP Study Personnel

SSA Sub-Saharan Africa

SN Study Nurse

SV Supervisor

UL Upper Safe Level

WHO World Health Organization

WKN West Kiang Number

**TABLE OF CONTENTS**

**Section 1: Summary**

**1.1 Background**

**1.2 Hypothesis**

**1.3 Methods and Design**

**1.4 Sponsors**

**1.5 Start Date**

**1.6 Finish Date**

**1.7 Expected Submission Date**

**Section 2: Background**

**Section 3: Aims and Objectives**

**3.1 Study Aim**

**3.2 Primary Objectives**

**3.3 Secondary Objectives**

**Section 4: Methods and Design**

**4.1 Design**

**4.2 Ethical Consideration**

**4.3 Intervention – Investigational Medical Product (IMP)**

**4.4 Setting**

**4.4.1 General**

**4.4.2 Community Involvement**

**4.5 Participants**

**4.5.1 General**

**4.5.2 Eligibility criteria**

**4.5.2.1 Inclusion criteria**

**4.5.2.2 Exclusion criteria**

**4.5.3 Enrolment**

**4.5.4 Special Circumstances**

**4.5.5 Immunizations**

**4.6 Subject Consent**

**4.7 Confidentiality**

**4.8 Randomization and Blinding**

**4.9 Subject Supplementation**

**4.9.1 General**

**4.9.2 Criteria for interruption of supplementation**

**4.10 Subject Follow-up**

**4.10.1 Follow-up during supplementation**

**4.10.2 Follow-up after supplementation**

**4.10.2.1 Recording frequency of self-referred clinic return attendances**

**4.10.2.1.1 Keneba**

**4.10.2.1.2 Karantaba/Kwinella Health Care Centers**

**4.10.2.1.3 Self reported clinic attendances**

**4.10.2.2 Anthropometry**

**4.10.2.3 Week 14 follow-up**

**4.11 Endpoints and Outcome Measures**

**4.11.1 Primary Outcome Measures**

**4.11.2 Secondary Outcome Measure**

**4.11.3 Details**

**4.12 Statistical Analysis**

**4.13 Documentation**

**4.14 Quality Assurance**

**4.14.1 IMP**

**4.14.2 Training of Trial Staff**

**4.14.3 Anthropometry Data Collection**

**4.15 Safety Considerations**

**4.15.1 General**

**4.15.2 Inclusion of a placebo control group**

**4.15.3 Drawing of blood**

**4.15.4 Breastfeeding**

**4.15.5 Adverse Event Reporting**

**4.16 Subject Withdrawal Procedures**

**4.17 Protocol Deviation/Violation Procedures**

**4.18 Perceived Benefits of the Study**

**4.19 Trial Management**

**4.20 Independent Supervision of the Trial**

**4.20.1 The Independent Trial Monitor**

**4.20.1.1 Patient safety**

**4.20.1.2 Progress of the trial**

**4.20.1.3 Adherence to protocol**

**4.20.1.4 Consideration of new information**

**4.20.1.5 Meeting schedule**

**4.20.2 The Data Safety Monitor**

**4.20.2.1 Data monitoring**

**4.20.2.2 Meeting schedule**

**4.20.2.3 Independent Trial Monitor and Data Safety Monitor visits**

**4.20.2.4 Data Safety Monitor/Violation plan**

**4.21 Financial considerations**

**Section 5: Figures and Tables**

**Section 6: References**

**Section 7: Appendices**

**Figures**

| **Figure 1:** | Influence of malnutrition on hospital case fatality rates for various diseases in The Gambia |
| --- | --- |
| **Figure 2:** | Study outline |
| **Figure 3:** | Outline of sampling frame work |

**Tables**

| **Table 1:** | Mean of all the visits per child (age 6 months to 2 years) in core study villages |
| --- | --- |
| **Table 2:** | Sample size calculations for various effect sizes and with different clinic visit rates achieving a power of 80% with significance level of 5% |
| **Table 3:** | Micronutrient composition of the supplements for infants (MMCT-LNS-1) and for children (MMCT-LNS-2). The composition of each supplement is based on 2xRDA/RNI according to age |
| **Table 4:** | Dietary Reference Intakes (DRI) (Recommended Daily Allowance (RDA) & Adequate intake (AI), Reference Nutrient Intakes (RNI) WHO and Reference Nutrient Intakes (RNI) UK and Upper safe limits (UL) of each micronutrient given according to age |
| **Table 5:** | Data Entry Flow Chart during MMC Trial at Keneba Field station |

**SECTION 1: SUMMARY**

**1.1 Background**

Malnutrition continues to be a major contributor to child morbidity and mortality worldwide. Acute and chronic deficiencies of multiple macro- and micro-nutrients are prevalent in many settings and affect a variety of health and disease outcomes. The interaction of micronutrients, immune function and infectious disease is complex and effects of micronutrients may be synergistic as well as opposing, supporting supplementation with multiple rather than single micronutrients. Multiple micronutrients (MMN) are already widely prescribed in clinics in the developing world with neither evidence nor international guidelines. Lipid-based MMN (LNS MMN) have been used successfully in the *treatment* of malnutrition and a wider research agenda is currently looking at the use of lipid-based nutritional supplements (LNS) for the *prevention* of malnutrition. LNS outperforms other forms of MMN supplementation in terms of growth. Additional advantages favouring translational applications include local production, long shelf life and safety.

Preventative trials of LNS involving *community-wide* supplementation are soon to be initiated elsewhere in Africa as part of the international LNS (iLNS) project. If successful, such community-wide administration would then require an efficient health-infrastructure and distribution system. Such systems are virtually non-existent in rural areas of SSA but rural health centres could provide a distribution base. We will therefore undertake a RCT of clinic-based LNS distribution with a specific target population, i.e. vulnerable children that self-select by presentation at the clinic. To our knowledge this is the first RCT of primary health care clinic based short-term LNS MMN supplementation.

**1.2 Hypothesis**

Physician prescribed LNS with a comprehensive set of micronutrients will improve the health status of children self-presenting to a primary health care clinic.

**1.3 Methods and Design**

This is a randomized double-blind placebo-controlled trial of 25g/d LNS (containing macronutrients and 23 micronutrients) under GCP standards for all children 6m-5y from West Kiang self-presenting to the clinic at MRC Keneba over 1 year (n=495 infants; n=1005 children). Subjects will be randomized into 3 groups: 1) 12wk LNS supplementation; 2) 6wk LNS supplementation followed by 6wk placebo; or 3) 12wk placebo. Morbidity and appetite will be assessed during supplementation by fieldworker (FW) visits to the homes of the participants. Compliance will be assessed by unannounced observations, supplementation usage and questionnaires.

The primary outcomes are health status as indicated by the frequency of self-referred clinic return attendances within 6m (with the possible extension of observations to 12m) from baseline specifying clinical diagnoses at each presentation to profile disease patterns and growth (weight, height/length, mid-upper arm circumference (MUAC)) at four points over 6m (0, 6, 12 and 24 weeks).

Changes in micronutrient status and standard haematology will be assessed in a randomly selected subgroup of 300 participants (n=100 LNS-6, n=100 LNS-12, n=100 LNS-0) at the end of the supplementation period (week 12). (Of note: The protocol was amended and an initial measurement of micronutrient status and standard haematology at day 10 was not measured.)

**1.4 Sponsors**

MRC and the UK Department for International Development (DFID) under the MRC/DFID Concordat agreement (MC-A760-5QX00)

**1.5 Start Date**

December 2009

**1.6 Expected Finish Date**

October 2011

**1.7 Expected Submission Date**

February 2012

**SECTION 2: BACKGROUND**

WHO regards hunger and malnutrition as the gravest threat to public health (Sheeran 2008). (1) Assessment of progress towards the Millennium Development Goals in 2007 showed that child survival rates only show slow improvement and are worst in sub-Saharan Africa. (2) It was estimated that if trends continue, the target of halving the proportion of underweight children will be missed by 30 million children. An estimate of 30% of child deaths and 29% of all disability-adjusted life-years (DALYs) worldwide are attributable to malnutrition in its various forms. (3, 4) Maternal and child undernutrition results in substantial increases in mortality and overall disease burden, exemplified by data from The Gambia. (5) There is a strong association between weight-for-height and hospital case fatality rates from most leading causes of death (See Figure 1). It is probable that malnutrition measured as weight-for-height correlates with multiple macro- and micronutrient deficiencies. Such deficiencies have been shown in the past to result in impairment to a number of components of the immune system. (6) Supplementation with specific micronutrients has proven efficacious as therapy and prevention for certain infectious morbidities (7), including vitamin A and measles infection (8) and zinc and diarrhoeal disease. (9-11) The inter-relationship between malnutrition and infection is complex though. (12)

Figure 1: *Influence of malnutrition on hospital case fatality rates for various diseases* *in The Gambia*. (5) *(Legend shows weight for height z-scores)*

There have been many strategies to combat malnutrition. Unfortunately, most of these have had only limited success. (7) Previous strategies included food supplements and mass fortification of staple foods. (13) Costs and the limited impact on child growth limits the long-term usage of food supplements in low-income countries. Mass fortification of staple foods with some key micronutrients (e.g. vitamin A, iodine, iron) has been attempted in several countries, but with limited success. (14, 15) Unfortunately, the most vulnerable age group (6 – 24 months) will have only a small degree of benefit as they consume too little of the fortified staple foods. Also, staple food may vary greatly between urban and rural areas, making targeted mass fortification difficult (personal discussion with NaNA).

Previously, most programmes concerned with elimination of micronutrient malnutrition have focused on single nutrients, particularly vitamin A, iodine and iron with some success. (4) More recently, multiple micronutrient supplementation trials, instead of single micronutrient supplementation, have been undertaken in the light of the limited resources that are available to many programmes and countries worldwide. (16, 17) MMN products such as Sprinkles®, tablets and syrups have been shown to reduce iron deficiency and anaemia for example. (13) Unfortunately, these supplementations do not provide a solution to adequate intake of energy, protein and fat in vulnerable populations and they have very limited impact on child growth. (7, 13)

In the *treatment* of severe malnutrition, Ready-to-Use-Therapeutic Foods (RUTF) such as Plumpy’nut have been very successfully used. Plumpy’nut was first developed by Nutriset at the end of last century as an alternative to milk-based liquid formulas for the treatment of severe malnutrition in children. Plumpy’nut is a type of lipid-based nutrient supplement (LNS) in which MMNs are embedded in fat, which protects vitamins against oxidation and increases the shelf life of the supplement. LNS does not contain water and hence does not support microbial growth. LNS can be safely stored and used even under poor hygienic conditions. Furthermore, LNS has a pleasant taste and hides the unpleasant taste of certain micronutrients better.

RUTF permit community-based rehabilitation of malnourished children and studies indicated that RUTF is better accepted by malnourished children than the equivalent milk-based formulas and leads to similar or greater weight gain. (18-20) RUTF is now used widely during relief operations and by humanitarian organizations to supplement other vulnerable groups such as moderately malnourished children, pregnant and lactating women and people living with HIV/AIDS. (21)

In the *prevention* of malnutrition LNS may be the most promising approach for preventing both micro- and macro-nutrient malnutrition among vulnerable target groups. Recent studies in Malawi and Ghana successfully used LNS for home fortification of complementary foods in the 6 – 12 months age group. (22-24) LNS in the Ghana trial was formulated with 23 supplementary nutrients including n-3 fatty acids, termed ‘Nutributter’ also produced by Nutriset. Nutributter has been shown to out perform other modes of MMN supplementation, particularly in terms of growth. This is consistent with the very positive outcomes seen when using RUTF for the treatment of severely malnourished children.

Several advantages make LNS a potential candidate for a breakthrough in nutritional strategies to prevent malnutrition:

1. It is a food-based approach that can use local products (e.g. peanuts), can be produced locally (Kenya, Malawi, Niger etc) and is hence commercially viable, helping to reduce poverty locally via production and distribution networks. LNS can be marketed via retail channels or through maternal and child health centres.
2. LNS provide essential fatty acids, which have been linked with improved growth and brain development in children. (25-28)
3. The fat content of LNS increases the energy density of complementary foods and may enhance absorption of fat-soluble vitamins such as vitamin A.
4. The dose can be easily divided over the day, thereby minimizing any potential hazard of single large doses of iron (e.g. in malarial areas (29, 30))
5. LNS have been shown to increase linear growth of children (22) and prevent severe stunting, an effect that has not yet been demonstrated with micronutrient powders.

The search for an appropriate target group to combat malnutrition in a cost-effective way and with wide impact is ongoing. It is known that micronutrient deficiencies in mother and infant have a gross effect on immune system development and growth of the infant. (4) Hence, main target groups are pregnant and lactating women and infants and young children with community-wide coverage. It has been shown that in the under 2 year old age group growth faltering is most evident and a proposed study recently presented to the MRC Gambia SCC committee will address this target group and investigate effects of pre-natal and infancy supplementation with MMN on infant immune development (SCC 1126, ISRCTN49285450). Beneficial effects of complementary feeding interventions were observed in diarrhoea and both upper and lower respiratory infections in a recent systematic review. (13)

MMN supplements are already frequently prescribed in clinics in the developing world. (31) Reasons for their prescription vary from obvious clinical signs of micronutrient deficiencies (e.g. vitamin A deficiency and Bitot’s spots), decreased appetite, ‘strengthening’ the child’s immune system, and prescription on demand by the parents, a phenomenon not unknown to the developed world. (32) There are currently no specific international clinical guidelines for the use of MMNs in these settings and their usefulness has never been evaluated. Supplementary multivitamins and minerals are recommended by the WHO for children with persistent diarrhoea for two weeks with little evidence. (33) In the developed world, multiple micronutrients are frequently advertised with the claim of preventing infections (e.g. vitabiotics.com). In the premature infant multiple vitamin drops are prescribed worldwide also with very limited evidence and based only on consensus of experts. (34)

Using a nutritional intervention in a primary health clinic setting may offer new insight into the effect of multiple micronutrient supplementation on future clinic presentations and disease patterns. It would also introduce a new strategic approach of targeting our struggle against malnutrition and its effect on global disease burden. A clinic-based intervention might have some important advantages over plans for community-wide coverage. These include: better targeting through an element of automatic self-selection of recipients; easier storage and distribution of supplements; lower cost arising from greater targeting and use of existing services to distribute; and potential encouragement of clinic attendance.

**SECTION 3: AIMS AND OBJECTIVES**

**3.1 Study Aim**

The aim of the study is to test the hypothesis that physician prescribed LNS with a comprehensive set of multiple micronutrients will improve the health status of children self-presenting to a primary health care clinic.

**3.2 Primary Objectives**

To assess the impact of LNS MMN supplementation on health status as indicated by

1. The frequency of self-referred clinic return attendances within 6m from baseline specifying clinical diagnoses at each presentation to profile disease patterns

AND

1. Growth (weight, height/length, mid-upper arm circumference (MUAC)) at four points over 6m.

**3.3 Secondary Objectives**

To assess the impact of LNS MMN on:

1. Micronutrient and haematological status in a randomly selected subgroup of 300 participants
2. Appetite

**SECTION 4: METHODS AND DESIGN**

**4.1 Design**

This study is a double-blind randomised, placebo-controlled trial. The outline of the study design is given in Figure 2 and 3.

**4.2 Ethical Consideration**

The trial is sponsored by the International Nutrition Group of the Medical Research Council (MRC), United Kingdom (UK). The principle investigator (PI) is Dr. Stefan Unger.

This trial has been approved by the Scientific Coordinating Committee (SCC) of MRC Laboratories, The Gambia and by the MRC / Gambian Government Ethics Committee including amendments.

The trial will be conducted according to the principles of Good Clinical Practice (GCP), as described in the MRC Guidelines for Good Clinical Practice in Clinical Trials (1998) and according to the MRC Good Research Practice guidelines (2005) (35, 36).

This trial has been approved by the London School of Hygiene and Tropical Medicine (LSHTM) ethics committee (No. 5629).

**4.3 Intervention – Investigational Medical Product (IMP)**

Supplies of the IMP and the placebo have been procured through Valid International, Nairobi, Kenya.

The IMP is a Lipid-based Multiple Micronutrient Supplement (LNS MMN)

The supplement is a lipid-based paste with 23 micronutrients. Two different fortified spreads will be used, one for the age groups 6 to 12 months (MMCT-LNS-1) and one for the age group 1 to 5 years (MMCT-LNS-2) adjusting for the differences in Recommended Daily Allowance (RDA) / Recommended Nutrient Intakes (RNI) at different ages. Most micronutrient levels within the formulation are aimed at approximately 2x RDA/RNI, with the exception of

1. Iron and vitamin A for reasons associated with safe upper levels.
2. Zinc based one recommendation from previous supplementation trials (10, 37) and current trials (SCC 967)
3. Calcium, Magnesium, Phosphorus and Potassium for technical reasons during supplement production (‘space’ limitation within the supplement).

The composition of both MMCT-LNS-1 and MMCT-LNS-2 is specified in Table 3. Recommended Daily Allowance (RDA) and Recommended Nutrient Intakes (RNI) are given for children <5 years in Table 4.

The Placebo (MMCT-LNS-0) is the lipid-based paste without added MMN. (See Table 3 for composition)

**4.4 Setting**

**4.4.1 General**

Kiang West is an isolated, rural district of the Lower River Division, The Gambia. The area is bounded to the north and west by the River Gambia, to the south by a tributary of the river named Bintang Bolong and to the east by a partially-surfaced road. It is a region of savannah-scrubland with a population of 13000 people, predominantly ethnic Mandinka, living by subsistence farming.

Primary health care is provided by 3 primary health care clinics, one in Karantaba run by the Government and supported for the last 40 years by the clinical staff of MRC, one in Kwinella run by the Government, and one in Keneba, within the MRC Field station. Paediatric secondary and tertiary health care services are provided at the Royal Victoria Teaching Hospital (RVTH) in Banjul or MRC Laboratories, Fajara, four hours by road from Keneba. In some very rare cases, paediatric cases are referred to Soma Regional Hospital and Bwiam Hospital.

The trial will recruit from 33 rural villages in Kiang West currently under the demographic surveillance of MRC Keneba. The study intervention will be community-based.

The Kiang West Demographic Survey (KWDS) is a computerized database containing basic demographic data on all the residents of West Kiang. It is updated every 3 months and migratory movements of people into, within and out of the survey area are tracked.

A newly developed electronic patient record system (KEMReS) will capture data on a) vital signs b) anthropometry c) symptoms and signs d) investigations e) diagnosis and f) treatment at every clinic visits as well as specified data at research follow-up visits.

**4.4.2 Community Involvement**

A series of meetings took place between the PI and community leaders in each village, including the Alkalos (village heads) and Imams (Islamic clerics). The purpose of these meetings was to explain the study and to gauge general community approval before proceeding.

**4.5 Participants**

**4.5.1 General**

Infants and children from the West Kiang Region of The Gambia, male and female, age 6m-5yrs, self-referred to the MRC Keneba clinic will be recruited using the Keneba electronic patient record system.

Each child will receive a laminated West Kiang Identification Card by the clinic reception staff, whether enrolled in the study or not. This ID card contains following information:

Name, West Kiang Number (WKN), Bar code for West Kiang Number

Each child between age 6 months and 5 years will then be seen in the clinic and after clinical management a doctor will assess eligibility.

**4.5.2 Eligibility criteria**

**4.5.2.1 Inclusion criteria**

- Age 6 months – 5 years
- Resident of West Kiang, The Gambia
- Self-referral to Keneba Health Care Clinic

**4.5.2.2 Exclusion criteria**

Children

- Out with the specified age group
- From regions other than the West Kiang Region of The Gambia
- Involved in another trial
- With known haemoglobinopathies
- With severe malnutrition
- Called to the clinic rather than self referred (e.g. for KWDS purposes, clinical follow-up appointments and immunizations unless they present with an illness)

**4.5.3 Enrolment**

Once the eligibility form has been signed by the doctor and the patient is eligible, the subject will be taken by the Study Personnel (SP) to the research room for consenting (See section 4.6 and Appendix 4 and 3 for information sheet and consent form).

Once consented, the patient will be entered into the computer (all demographic details should be available, including the WKN from the electronic patient records system) and a study ID number will be assigned automatically which should be written by the SP on the back of the West Kiang Medical Card using a permanent black marker.

The computer screen will show the SP, which group the subject has been randomized to and whether the subject falls into the subgroup for investigations into micronutrient and haematological status. (See below)

Information on the supplementation and a supply of 2 weeks of supplement will be given to the carer, once the child is enrolled.

**4.5.4 Special Circumstances**

In following circumstances, supplementation will be started only AFTER successful *treatment* of:

1) Severe Malaria

2) Severe Sepsis

Specific criteria of ‘severity’ will apply for malaria and sepsis.

Severe falciparum **malaria** – Definition (38)

Patient presents with confusion, or drowsiness with extreme weakness (prostration) and/or one or more of the following:

- Cerebral Malaria (defined as unrousable coma not attributable to any other cause in a patient with falciparum malaria)
- Severe normocytic anaemia
- Hypoglycaemia
- Metabolic acidosis with respiratory distress
- Fluid and electrolyte disturbances
- Acute renal failure
- Acute pulmonary oedema
- Circulatory collapse, shock, septicaemia
- Abnormal bleeding
- Jaundice
- Haemoglobinuria
- High fever
- Hyperparasitaemia

Severe **Sepsis** – Definition using emergency signs of severe illness (39)

Any patient presenting with one or more of following emergency signs:

- Obstructed breathing
- Severe respiratory distress
- Central cyanosis
- Signs of shock (capillary refill longer than 3 seconds; weak, fast pulse)
- Coma
- Convulsion
- Signs suggesting severe dehydration in a child with diarrhoea (any two of the following: lethargy, sunken eyes, very slow return after pinching the skin)

In the following circumstances, supplementation will be started only AFTER successful improvement of the condition:

Severe Anaemia (Hb < 5g/L) - improvement to Hb > 7g/L (33)

Treatment will be in accordance with the Gambian/IMCI/WHO guidelines (33, 40, 41) with regular audits of clinical management organized by the Audit Nurse (AN) utilizing the Keneba electronic patient record system.

*Of note:* The health service at MRC Keneba does not have inpatient facilities. Any child with severe illness and in need of hospital admission will be referred to a hospital after stabilization and doctors’ assessment. Also, children in need of further investigation that cannot be provided in Keneba will be referred. On return the children will be consented for and enrolled into the study if eligible.

**4.5.5 Immunizations**

For those children that present to the clinic whose immunization status is not up to date will be offered immunization according the Gambian EPI vaccine schedule. All children will also be seen at the additional time points for administration of the EPI vaccines. For subjects outside of the core villages (Keneba, Kantong Kunda and Manduar), administration of EPI vaccines will be co-ordinated with the RHT trekking team. Vitamin A supplementations received by each of the study participants during the trial will also be entered to assess if it has any influence over the frequency of morbidity of the subjects.

**4.6 Subject Consent**

Eligible subjects with their carers will be invited to give full, informed consent to take part in this trial. Carers unwilling for the child to participate will be thanked for their time and co-operation.

A trained SP will explain the full details of the study to the carer, covering all aspects of the study as laid out in the Subject Information Sheet (Appendix 4). Illiterate carers will additionally have the full information sheet read to them; literate subjects will be allowed to read the information sheet in their own time. Any questions that arise will be answered by the SP, or referred to the PI for clarification. Carers will also be given the possibility to speak to one of the study investigators if they wish. If the carer agrees to participate, written consent will be obtained. (Appendix 3) The right thumbprint will be taken in lieu of a signature from carers who cannot write. During the consent process it will be stressed that children are free to refuse or withdraw consent to be involved in the study without compromising the standard clinical care they can expect from MRC Keneba for themselves and their families. If the escort of the child is unable to consent for the child during the clinic visit, a FW will visit the child at home and consent the appropriate guardian.

**4.7 Confidentiality**

All information obtained on subjects during the trial will be kept confidential and will be accessible only to relevant SP within the trial and to clinical staff involved in the care of the subject. Subject confidentiality, privacy and anonymity will be ensured at all times, and the standards set by the Scientific Coordinating Committee (SCC) and MRC/Gambian Government Ethics Committee will be followed. The unique study identification numbers (ID) and the WKN will be used throughout the study, on all samples and data forms generated. Linkage of the ID numbers back to the subject will not be possible without referring to a lookup table. Only key study personnel will hold a copy of this table. Analyses will be performed on an anonymous copy of the data. At all stages, staff/collaborators responsible for sample analysis will be blinded as to the subject’s identity. Together, these processes will ensure complete confidentiality of the data gathered.

**4.8 Randomization and Blinding**

Once consent is obtained the subjects will be randomized into one of 3 groups:

|  | **Week 1-6** | **Week 7-12** |
| --- | --- | --- |
| (1) Placebo | Placebo | Placebo |
| (2) 6 week supplementation | Supplement | Placebo |
| (3) 12 week supplementation | Supplement | Supplement |

Balance with respect to date of recruitment is achieved using block randomization. A block size of 12 is used: four of each of the three supplements are allocated randomly within each block. Type of supplement was randomly allocated to the study numbers in this way before the start of the study. Participants are allocated their study number using the randomization table within the study database (SDB) in the sequence in which they are recruited. Randomization is stratified by severity of illness by using a separate randomization block with a smaller block size of 3 for patients randomized after registering with illnesses.

One complication involves the intermediate group who receive the supplement for 6 weeks and then the placebo for the final 6 weeks. This could potentially reveal the content of the pots and hence break the treatment code. To reduce bias 18 letters for each 6-week supplementation period, e.g. ‘A 1-6’ and ‘A 7-12’ are used.

The supplement has been blinded and only the supplement provider (Valid) and a person who is not related to the study hold the key. The key holder within the MRC Gambia is Dr Kerry Jones.

Once consented and enrolled into the study, a study ID will be given by the computer in addition to the assignment to one of the groups above and whether the child is part of the subgroup testing for changes in haematological and micronutrient status.

**4.9 Subject Supplementation**

**4.9.1 General**

The supplement will be administered in small pots (animal code according to randomization) and given to the carer on a fortnightly basis. Infants (at the time of enrolment) receive pots with a red cap (includes MMCT-LNS-0 and MMCT-LNS-1); children receive pots with a blue cap (includes MMCT-LNS-0 and MMCT-LNS-2). The carer will be asked to administer the supplement on a daily basis by mixing 2 leveled tablespoons of the supplement/placebo (equivalent to 20g of the supplement/placebo) with the food for the child. Mothers/guardians will be encouraged to give the study child the full dose, and compliance will be assessed by both unannounced observations (minimum of two per subject), supplement usage and by a questionnaire administered on a daily basis for the first week and fortnightly thereafter. (See Appendix 6) Previous supplementation trials conducted by MRC Keneba have observed high levels of compliance (42) and we anticipate that this study will enjoy the same benefits.

The subject will also be asked to report any adverse event (AE) that might have occurred since last seeing a SP. (See section 4.15.5)

If the subject cannot be found, the supplement will be delivered to the subject as soon as possible.

**4.9.2 Criteria for interruption of supplementation**

If a subject *on* supplementation presents to the clinic with severe malaria or severe sepsis and/or severe anaemia (See section 4.5.4), supplementation should be suspended until after successful management of the condition. The illness should also be recorded as a serious adverse event (SAE). (See section 4.15.5)

**4.10 Subject Follow-up**

Please see Figure 3 for the sampling outline for this study.

**4.10.1 Follow-up during supplementation**

During the supplementation period of 12 weeks, the SP will visit the subject fortnightly, supplying the carer with supplement and completing an appetite and morbidity questionnaire detailing especially morbidity from diarrhoea and acute lower respiratory infection. (Fortnightly Compliance, Appetite and Morbidity Questionnaire in Appendix 6) At week 6 and 12 the questionnaire will be completed at MRC Keneba Field station during follow-up visits for anthropometry (See below). During the first week of supplementation, the SP will visit the subject every day to monitor closely initial improvement of appetite and resolution of symptoms. (See First Week Compliance, Appetite and Morbidity Questionnaire in Appendix 6) Information of the questionnaires collected will be entered by a trained SP into the study database at Keneba Field station. The forms will then be sent for second data entry to the data office.

At 6 weeks, 12 weeks and 24 weeks from baseline, the subject will be invited to the MRC Keneba field station for growth measurements. Transport will be provided by the MRC. At each visit all information will be collected and entered into the Keneba electronic patient record system. At the end of each visit (including the initial presentation visit), a form with all required data will be printed out, signed by the nominee and stored in the Case Report Form (CRF) for that subject. (See CRF Appendix 6)

The randomly assigned subgroup of 300 children for haematological and micronutrient status measurements will also be asked to come to the MRC Keneba field station 12 weeks after recruitment for blood tests. 5 ml of blood will be taken, the Hb will be measured and the sample will then be prepared and stored according to Keneba SOP 3006 for future analysis of Zn, Se, Vit D and retinol status. Again, all information will be collected and entered into the Keneba electronic patient record system (See Table 5) and a copy of the data of these visits will be printed out, signed by the nominee and stored in the CRF. (See CRF Appendix 6)

An overview of patient presentations and data recording is given in Table 5. Alerts for the various presentations/visits will be automatically shown during data entry into the Keneba electronic patient record system. Call lists for follow up are generated.

Any referrals to other health care facilities will also be followed up, in terms of diagnosis, treatment and outcome.

**4.10.2 Follow-up after supplementation**

**4.10.2.1 Recording frequency of self-referred clinic return attendances**

**4.10.2.1.1 Keneba**

The frequency of self-referred clinic return attendances for each participant is monitored over 6 months (with the possible extension of 1 year from baseline) using the Keneba electronic patient record system. Clinical diagnoses at each presentation are specified to profile disease patterns.

**4.10.2.1.2 Karantaba/Kwinella Health Care Centres**

In addition, data on attendance at Karantaba and Kwinella Health Care Centres, small government run primary health care centres in West Kiang, is recorded. There is limited scope at these Health Centres to obtained detailed information on vital signs, diagnoses, investigations and treatment.

Every week, a SP will visit each health centre and collect information on clinic visits of any enrolled MMCT subject using the Government Attendance Logbook and a study logbook provided by the MRC. Details include date of visit, diagnosis and treatment.

**4.10.2.1.3 Self reported clinic attendances**

During the home and Keneba visits, the carer will also be asked whether they have attended any clinic since they last saw a SP (See CRF Appendix 6). This data will be compared to the data collected from Keneba, Kwinella and Karantaba.

**4.10.2.2 Anthropometry**

Anthropometry measurements will be taken at week 24 as specified in section 4.11.

**4.10.2.3 Week 14 follow-up**

Home visits for all subjects will be conducted at week 14 to assess any possible impact of the lipid paste.

**4.11 Endpoints and Outcome Measures**

**4.11.1 Primary Outcome Measures**

1. The frequency of self-referred clinic return attendances within 6m from baseline specifying clinical diagnoses at each presentation
2. Growth at 6, 12 and 24 weeks

**4.11.2 Secondary Outcome Measures**

1. Change in appetite during the supplementation period from baseline (day 1) to 2, 4, 8, 10 and 12 weeks.
2. Change in morbidity status during the supplementation period from baseline (day 1) to 2, 4, 8, 10 and 12 weeks.
3. Change in haematological status after the initial acute phase (10 days) to 12 weeks.
4. Change in micronutrient status after the initial acute phase (10 days) to 12 weeks.

**4.11.3 Details**

**4.11.3.1 Growth**

Growth measurements include weight, height/length and mid-upper arm circumference (MUAC).

**4.11.3.2 Appetite**

Appetite is assessed by maternal report based on a method validated in Lima, Peru. (43) (See Appendix 6)

**4.11.3.3 Morbidity**

Morbidity is assessed by questionnaires (See Appendix 6) and records on clinic presentations (See section 4.10.2.1)

At week 12 visit, 5ml of blood will be taken from subjects randomised to the micronutrient and haematological status subgroup to check for:

**4.11.3.4 Haematological Status**

Haemoglobin concentration measure at Keneba Field station.

**4.11.3.5 Micronutrient status**

Plasma Zinc, Retinol, Vitamin D (25 (OH)D) and Selenium concentration will be analyzed at the Human Nutrition Research (HNR) Unit, Cambridge, UK.

**4.12 Statistical Analysis**

We found no published data on the effect of human nutritional intervention on repeat clinic visits specifying disease pattern in this age group on which to base sample size calculations for outcome measures.

In Keneba, there are over 1000 clinic visits for the age group 6 – 12 months and 2000 clinic visits for the age group 1-5 years per year. Approximately, 25% of clinic visits are repeat visits for that year, i.e. return visits made by those who visit at least once.

From our data on clinic visits of children less than 3 years of age from the three core villages Keneba, Manduar and Kantong Kunda in West Kiang, we can calculate the mean and variance for all children between the ages of 6 months and 2 years. The upper limit of the age here is because we wanted to count the number of visits during the year following the first visit. The mean in the table below refers to all the visits (minimum of 1 per child). In order to convert to the number of return visits (our main outcome), we subtract one from the mean but the variance and sd remain unchanged.

Table 1: *Mean of all the visits per child (age 6 months to 2 years) in core study villages.*

| Interval | mean | var | Sd/mean |
| --- | --- | --- | --- |
| 6mth | 5.34 | 11.23 | 0.628 |
| 1 year | 8.59 | 29.29 | 0.630 |

From this we obtain an estimate of the sd/mean ratio and note that it is fairly constant (as predicted for a very dispersed negative binomial distribution). So if we have an estimate, λ, of the mean number of visits we expect to be made by those children who visit at least once then we can calculate the expected std deviations of the number of return visits for children in the placebo and intervention arms of our study. If treatment reduces the number of return visits by a factor of φ, then, for control arm: sd=0.63λ; for treatment arm: sd=0.63(λ-φ(λ-1)). From these we can estimate the required sample size for significance level α and power β:

N = [Φ^-1^(1-α/2)+Φ^-1^(β)]^2^ . (σ_0_^2^+ σ_1_^2^) / [μ_1_-μ_0_]^2^

= [Φ^-1^(1-α/2)+Φ^-1^(β)]^2^ . 0.63^2^(λ^2^+ [λ-φ(λ-1)]^2^) / [φ(λ-1)] ^2^

where α=-0.05 and β=0.8

Φ^-1^(1-α/2)+Φ^-1^(β) = 1.96 + 0.84 = 2.8

so:

N = 2.8^2^ . 0.63^2^ . (λ^2^+ [λ-φ (λ-1)]^2^) / [φ (λ-1)] ^2^

We can now tabulate the required N, the number of children we need to recruit to detect the difference should it exist. We use the mean for clinic visits per child over a period of 12 months (our extended follow-up period) for λ, which is 8.59 according to our calculations above. Table 2 gives the required numbers to achieve a power of 80% with significance level of 5%.

The mean of the number of clinic return visits we used in the calculation (λ=8.59) is likely to be a little higher than average as we based it on our data on children less than 3 years from our local core study villages. It is very likely, that younger children and those living near to Keneba are much more likely to attend. In our study we will look at the age group 6 months to 5 years from villages all across West Kiang. Hence, we need to adjust our sample size accordingly. We concluded that a sample size of 500 per group would allow us to detect a reasonable difference in rate of clinic return visits even if the average of the number of clinic visits is lower (e.g. half: λ=4.295 as calculated in Table 2) and considering a drop out proportion of 10%. With this sample size we would expect to detect a difference of 10-20% between the groups.

Table 2: *Sample size calculations for various effect sizes and with different clinic visit rates achieving a power of 80% with significance level of 5%.*

|  | Main outcome: Rate of clinic return visits over 12 months follow-up period | | | | | |
| --- | --- | --- | --- | --- | --- | --- |
|  | Based on mean rate of λ=8.59 of all clinic visits | | | Based on mean rate of **λ=4.295** of all clinic visits | | |
| Effect size | Placebo | 6 wk LNS intervention | 12 wk LNS intervention | Placebo | 6 wk LNS intervention | 12 wk LNS intervention |
| 5% | 3394 | 3394 | 3394 | 4528 | 4528 | 4528 |
| 8% | 1475 | 1475 | 1475 | 1974 | 1974 | 1974 |
| 10% | 812 | 812 | 812 | 1089 | 1089 | 1089 |
| 15% | 345 | 345 | 345 | **466** | **466** | **466** |
| 20% | 186 | 186 | 186 | 252 | 252 | 252 |

We will employ an intention-to-treat analysis throughout. Each analysis will address two questions: (i) do the treatment groups perform better than the placebo group and (ii) do those receiving 12wk supplementation fare better than those receiving the supplement for only 6 weeks. As the primary outcome we wish to estimate φ and test whether it differs from zero. We will fit this parameter in a negative binomial model relating the number of self-referred return visits to treatment group, village, age and sex of child, season. Season will be fitted using truncated Fourier series.(44, 45)

In addition we will perform similar analyses but restricted to visits for certain conditions. We will reduce the anthropometry outcomes to z-scores (relative to WHO standards) before analysis. These will be regressed on age, sex, season and treatment group using least squares regression. Each anthropometry time point will initially be analyzed separately. The time points will then be pooled and analyzed with a random effects model to gain statistical power and investigate how sustained the effect of treatment is.

Follow-up data (appetite, morbidity) will be double entered into a computerised database and verified on the day of collection. Pre-arranged internal validity checks will be made.

A complete copy of the raw dataset will be kept with the data management team at MRC Field station, Keneba.

**4.13 Documentation**

All versions of the trial protocol and appendices, MMCT Study Manual, amendments, patient information sheet, consent form, field source documents, CRFs, invoices for trial purchases and correspondence with SCC, Ethics Committee and trial supervisory bodies will be retained by the PI as source documents. Original copies of the completed consent forms and CRFs will be held as specified below. All documents will be made available on request for independent review by the appropriate bodies.

| **Document Type** | **Minimum retention time** |
| --- | --- |
| Archives of superseded Procedure Documents, SOPs | 5 years |
| Consent forms and other volunteer information | 20 years |
| Research records and other operational records, e.g. laboratory notebooks | 10 years after publication |
| Research records relating to clinical studies | 20 years |
| Invoices, budgets and expenditure | 5 years |

Please also refer to

- Keneba SOP 1006 (Data management of projects in West Kiang)
- MRC ‘Good Research Practice’ 2005 (36)
- MRC ‘Guidelines for good clinical practice in clinical trials’ 1998 (35)
- MMCT Study Manual

**4.14. Quality Assurance**

**4.14.1 IMP**

The PI will hold documentation of the total amount of IMP procured for the trial. The expiry date for the MMCT supplement will be determined by Valid International, the producer of the IMP.

**4.14.2 Training of Trial Staff**

Initial training, further training, mini-assessments and refresher courses will be held in relevant procedures throughout the trial. Weekly meetings of senior staff (FWSV, SN, AN, Doctors) will be held in order to identify and correct problems in data collection, as they arise.

**4.14.3 Anthropometry Data Collection**

Anthropometry will be performed by FW’s working in pairs and cross checking readings. Two sets of data will be entered. If they are the same or within the accepted error range, data set 1 will be used. If the two data sets are different, measurements have to be repeated. Specific checks are set within the Keneba electronic patient record system. Anthropometry is performed according to Keneba SOP 2009. FW’s involved in anthropometry measurements get repeated mini-assessments and refresher training throughout the trial. Anthropometry equipment is checked weekly.

**4.15 Safety Considerations**

**4.15.1 General**

Deficiencies of both macro- and micronutrients are still common across much of SSA, including The Gambia affecting a variety of health and disease outcomes. MMN supplements are frequently prescribed in clinics in The Gambia. There are however no specific guidelines for the use of MMNs in the clinics as evidence for their effectiveness is missing supporting the conduction of a our trial.

There are risks of ingestion of high doses of specific micronutrients, especially iron and/or vitamin A and hence the level of iron and vitamin A is set below 2xRDA/RNI in the supplement. Most other micronutrient levels within the lipid-based formulation are based on 2x RDA/RNI (46) for the age groups and within known UL ranges. (47-49)

Also, the dose of LNS provided can be easily divided over the day, thereby minimizing any potential hazard of single large doses of iron. (29, 30) Considering the complex interaction of iron in infection (50, 51), we will omit supplementation during the treatment phase of severe malaria and sepsis to minimize any potential risk to the children. We exclude children with severe malnutrition, who will be treated according to WHO standards.

A recent study in Ghana demonstrated a beneficial effect of a similar LNS, given to infants from 6-12 months of age, on infant growth and motor development, with no adverse effects reported. It is therefore believed that the interventions in this study pose no risk to the children. (22)

Finally, a data and safety monitor (DSM) will be in place for this study.

**4.15.2 Inclusion of a placebo control group**

For ethical reasons, the use of a placebo control group requires careful consideration and justification. We do, however, believe that the use of a control group in our case is necessary and ethical for a number of reasons:

There are no guidelines for MMN supplementation in The Gambia, and by withholding supplement from the placebo group, children will not be placed in any danger. Although widely prescribed, MMN supplementation is not a proven therapy for children presenting to primary health care clinics. As an area lacking in research, and relevant to the health of a significant proportion of children in the developing world, our proposed study has explicit scientific and public health merit. The potential benefit in generating knowledge and improving child health outweighs the minimal risks involved. Secondly, informed consent will be obtained from all participating carers, who may withdraw their child from the study at any time. As is routine in similar studies in The Gambia, participating children will be visited regularly by SP for health checks during the supplementation period and in the event of illness will receive immediate free medical attention, and carers with their child will be given priority access to transport and the clinic.

**4.15.3 Drawing of blood**

The risks of drawing blood from infants include temporary discomfort, potential bruising, bleeding, and extremely rarely, infection. Venepuncture will be conducted under hygienic standards by a qualified nurse, and all attempts to minimize discomfort will be made.

For details please refer to Keneba SOP 3006.

**4.15.4 Breastfeeding**

WHO recommends exclusive breastfeeding to six months of age, a recommendation endorsed in The Gambia by the National Nutrition Agency (NaNA) and Department of State for Health and Social Welfare (DoSHSW). A clinic-based trial of nutritional supplementation before 6 months of age would not therefore be appropriate in this setting. We hence concentrate on the 6-month to 5-year age group.

**4.15.5 Adverse Event Reporting**

An AE is defined as any untoward medical occurrence in a trial subject. A SAE is defined as an AE that results in death, is life-threatening, requires inpatient hospitalization, results in significant disability or incapacity. Reported deaths will be recorded as SAEs. The PI or medically qualified deputy will report all SAEs to the DSM (See below) as soon as possible, after the research team has recorded them. AEs will be reported prior to an interim analysis of safety data at 6 months.

Reports of AEs and / or SAEs may, at the discretion of the independent trial monitor (ITM), result in the premature termination of supplementation of individual subjects in the trial. Such subjects will remain in the trial and be included in the analysis. The independent assessment of the emerging pattern of SAEs or other relevant information may warrant termination of the trial at any stage, by agreement.

In detail:

Any AE should be recorded for that subject in a separate MMCT Adverse Event Form. (Appendix 7) If the event is deemed a SAE, a MMCT Serious Adverse Event Initial Reporting Form (Appendix 8) should be completed by the PI or deputy and a copy should be send to the DSM within 5 working days. A MMCT Serious Adverse Event Follow-Up Form should be completed by the PI or deputy once the case has been closed and a decision of actions has been made. (Appendix 9)

Any patient currently receiving supplementation who presents to the clinic with an illness should be recorded as an AE or a SAE.

All AEs and SAEs should be recorded in the MMCT AE/SAE logbook and in the summary of AEs and SAEs in each CRF. (Appendix 6) If a child is transferred to the coast (MRC Fajara) for hospitalization, this should be recorded as a SAE.

Please also refer to the MMCT Study Manual.

**4.16 Subject withdrawal procedures**

If a subject withdraws from the study, it should be recorded in the MMCT Withdrawal Log Book. It should also be noted in the CRF of the subject under ‘Study Conclusion’. (Appendix 6)

**4.17 Protocol Deviation/Violation Procedures**

Definitions (The Institute for Clinical Research (ICR) guidance):

*Deviations* are any variance in the approved study protocol, criteria or procedure that does not affect the participant’s safety, rights, welfare or the integrity of the study and its resultant data.

*Violations* to the protocol are deviations that increase the risk or decrease the benefit and/or affect the participant’s rights, safety, welfare and/or integrity of the resultant data.

Specific to this study:

If a protocol deviation/violation is observed, the PI should be informed as soon as possible and following steps should be completed:

- Fill in a MMCT Protocol Deviation/Violation Report (Appendix 10)

If the protocol deviation may constitute a violation, the report should be sent to the ITM within 5 working days. A corrective action plan outlining what safeguards/steps the PI has put in place, or taken, to prevent the violation from occurring again.

If the subject is withdrawn from the trial, the MMCT Withdrawal Log Book and the CRF (Appendix 6) should be filled in appropriately.

Please also refer to the MMCT Study Manual.

**4.18 Perceived Benefits of the Study**

During the study period, there will be increased surveillance within the West Kiang region including the early detection of illness.

This study represents a major research commitment to child nutrition by the MRC International Nutrition Group and the infrastructure established for this trial, such as the newly built research clinic and the new Keneba electronic patient record system will facilitate other studies in the future and further improve patient care. This project will employ and train a large number of Gambian staff, equipping them with knowledge and transferable skills.

The study will help to establish whether short-term supplementation of MMNs through primary health care clinics is an effective and targeted way of combating MMN deficiencies in The Gambia. Availability of such data will help agencies such as the National Nutrition Agency (NaNA) implement policy aimed at optimizing nutritional status and hence decreasing disease burden of a targeted paediatric population.

**4.19 Trial Management**

The Principal Investigator (PI) will hold ultimate responsibility for the day-to-day running of the trial, according to the principles of MRC GCP. (35) The PI (or medically qualified deputy), in the capacity of a medical officer for MRC Keneba, will also maintain overall clinical responsibility for the subjects during the trial, up to the point of secondary referral, when such referral is deemed appropriate.

One senior nurse will be allocated to be the overall supervisor (SV) of the trial. Two senior FWs will be allocated to be the fieldworker supervisors (FWSV) overseeing the FWs posted in the field. One senior nurse will act as the audit nurse (AN) who is allocated to be in charge of auditing clinical management within Keneba using the Keneba electronic patient record system and comparing it to WHO standards and organizing further training when required.

**4.20 Independent Supervision of the Trial (35)**

The supplementation phase of the trial will last for 12 months from the commencement of recruitment. In keeping with the principles of GCP, the following trial oversight arrangements are in place:

Two external monitors, an Independent Trial Monitor (ITM) and a Data Safety Monitor (DSM) will review the study at intervals.

**4.20.1 The Independent Trial Monitor**

The role of the ITM is:

1. To monitor adherence to trial protocol and supervise the progress of the trial toward its objectives;
2. To review at regular intervals relevant information from other sources;
3. To consider the recommendations of the DSM;
4. To consider and provide advice to the MRC if required on any aspect of the trial, independently of the PI.

**4.20.1.1 Patient safety**

The ITM’s primary concerns are the rights, safety and well being of the individual trial subjects. These are the most important considerations in the conduct of the trial and should prevail over the interests of science and society. The ITM will consider the results of any interim analyses of trial safety data performed by the DSM and will have the authority to terminate the trial based on the information provided and the recommendation of the DSM.

**4.20.1.2 Progress of the trial**

The ITM should monitor the progress of the trial in order to maximize the chance of it being completed within the agreed timescale.

**4.20.1.3 Adherence to protocol**

The ITM should ensure that there are no major deviations from the trial protocol. If the PI wishes to make any major changes to the protocol during the course of the trial, the ITM should be consulted and approval sought from the SCC and Ethics Committee. Any protocol violations should be reported to the ITM (See Appendix 10 and section 4.17).

**4.20.1.4 Consideration of new information**

The ITM should consider new information relevant to the trial including reports from the DSM and the results from other studies. On the basis of this information, the ITM may recommend appropriate action, such as changes to protocol, additional information being made available to subjects or stopping the trial.

**4.20.1.5 Meeting schedule**

The ITM will review the full protocol prior to the recruitment phase of the trial in discussion with the PI. The ITM’s endorsement of the protocol is mandatory before the trial begins.

It is proposed that the ITM and PI meet in The Gambia / UK every 6 months while the trial is ongoing, in order to monitor and discuss progress and the interim analyses / concerns of the DSM, especially relating to patient safety. At the end of the supplementation phase of the trial, the PI will submit a report to the MRC SCC in The Gambia, based on a standardized template. This report will be endorsed by the ITM and will be used in support of any further application to continue supplementation beyond the agreed duration of the current trial.

**4.20.2 The Data Safety Monitor**

The role of the DSM is:

1. To review reported SAE’s by the PI or deputy.
2. To determine if additional interim analyses of trial data should be undertaken;
3. To consider the data from interim analyses, un-blinded if considered appropriate, plus any additional safety issues for the trial and relevant information from other sources;
4. To report to the ITM and to recommend on the continuation of the trial;
5. To consider any requests for release of interim trial data and to recommend to the ITM on the advisability of this;
6. In the event of further funding being required, to provide to the ITM and MRC appropriate information and advice on the data gathered to date that will not jeopardize the integrity of the study.

The DSM is stationed in The Gambia and is a medically qualified person with experience in clinical trials.

**4.20.2.1 Data monitoring**

The DSM will ultimately determine the manner in which it will monitor the data and what it requires from the PI in order to do so. This information is to be communicated to the PI. This will also determine if further interim analyses are required and conduct these in a manner which does not undermine the integrity of the trial.

**4.20.2.2 Meeting schedule**

The PI or DSM may call a meeting at any time.

It is proposed that the DSM undertakes an interim analysis of the safety data pertaining to the occurrence of AEs at the end of 6 months. The PI and trial statistician will provide the required data for this analysis. The DSM will make a full, confidential written report to the ITM, incorporating any other material relevant to the role of the DSM described above and advising on whether the trial should be stopped in light of the information ascertained. The ITM will decide whether to act on such advice and will inform the MRC accordingly.

Further analysis will be arranged every 6 months, or more frequently, while the trial is in progress.

**4.2.20.3 Independent Trial Monitor and Data Safety Monitor visits**

The Independent Trial Monitor and Data Safety Monitor made an initiation visit to the site before the start of the trial and made some recommendations. They will also be conducting a possible follow-up visits after 6-month interim analysis.

**4.20.2.4 Data Safety Monitoring during the MMC Trial**

- Qualitative reporting by PI during first 3 months on any noted differences in AE’s between supplements
- SAE’s to be analysed monthly by DSM
- Analysis of all AE’s and SAE’s after 6 months

6-month analysis should be presented blinded by the ING group to the Safety Monitor in the following format:

| Supplement | | Number of AE’s | Number of SAE’s | Self-referred clinic attendances | Other |
| --- | --- | --- | --- | --- | --- |
| No. | No |  |  |  |  |

Poison model (incidence per week or month) comparing the difference in incidence of AEs and SAEs and Self-referred clinical attendances with incidence rate ration, confidence interval and p-value.

Row 1 compared to Row 2

Row 1 compared to Row 3

Row 2 compared to Row3

**Protocol deviations/violations**

- Protocol violations are reported by the PI to the ITM within 5 days of observation
- Protocol violations to be assessed monthly by the ITM
- Summary of protocol deviations to be assess after 6 months during 2^nd^ site visit

#### 4.21 Financial Considerations

#### The trial is funded by the MRC and the UK Department for International Development (DFID) under the MRC/DFID Concordat agreement (MC-A760-5QX00).

# Figure 2: Study Outline

Demographic Data

DATA COLLECTION

DATA ENTRY, ANALYSIS AND PRESENTATION

Self-referred presentation to the MRC clinic

**Initial clinic visit and each self-referred return clinic visit:**

- Demographic Data
- Vital signs
- Anthropometry
- Symptoms
- Clinical Signs
- Investigations
- Diagnosis (ICD-10)
- Treatment

**Community follow-up for 12 weeks:**

- Appetite
- Morbidity
- Compliance
- Adverse Events

**Community follow-up at week 14**

Normal clinical management:

- Issue of WKN if not available/WK card
- Vital Signs/Anthropometry
- Doctor Assessment / Investigations / Treatment

Identification of eligible subjects:

- Check for inclusion/exclusion criteria
- Check for cases of delayed supplementation
- Blind allocation to one of four colour-coded groups
- UNIMMAP vs. Placebo
- Daily supplement
- Fortnightly monitoring by TBA / FW

Laboratory Data

**Day 10**

- Hb
- Zn
- Retinol
- 25 (OH)D
- Selenium

**Week 12:**

- Hb
- Zn
- Retinol
- 25 (OH)D
- Selenium

Consent:

- Information to carer
- Consent/Enrolment

Randomisation I:

- 12 wk supplementation
- 6 wk supplementation/6wk placebo
- 12 wk supplementation

Randomisation II:

- Participant randomised for further laboratory investigations? (n=300)

**Recruitment:**

- Vital signs (BP, O_2_ saturation, HR, RR)
- Height
- Weight
- MUAC
- SF

**6, 12 and 24 weeks:**

- Weight
- Height
- MUAC
- SF

**Each self-referred return clinic visit:**

- Vital signs (BP, O_2_ saturation, HR, RR)
- Height
- Weight
- MUAC
- SF

Biophysical Data

No:

- No Laboratory Investigations

Yes:

- Blood test at day 10 & week 12

Follow-up:

- Record clinic attendance for 12 months
- Home visits daily for first week
- Home visits fortnightly for remaining supplementation weeks
- Home visit at week 14
- Follow up at Keneba Fieldstation at week 6, 12 and 24

Figure 3: *Outline of the sampling frame work*

Table 3: *Micronutrient composition of the supplements for infants (MMCT-LNS-1) and for children (MMCT-LNS-2). The composition of each supplement is based on 2xRDA/RNI according to age.*^1^

| **Nutrient** | **Unit** | **MMCT-LNS-1** | **MMCT-LNS-2** | **MMCT-LNS-0** |
| --- | --- | --- | --- | --- |
|  |  | 20g | 20g | 20g |
|  |  |  |  |  |
| **Potassium** | mg | 152 | 152 | 60 |
| **Calcium** | mg | 200 | 220 | 20 |
| **Phosphorus (excluding phytate)** | mg | 82 | 82 | 35 |
| **Magnesium** | mg | 16 | 16 | 11 |
| **Iron**^2^ | mg | 12 | 12 | 0.4 |
| **Zinc**^3^ | mg | 10 | 20 | 0.2 |
| **Copper** | mg | 0.4 | 0.6 | 0.02 |
| **Selenium** | mcg | 20 | 40 | 1.3 |
| **Iodine** | mcg | 180 | 180 | 1.02 |
| **Manganese** | mg | 0.08 | 0.08 | 0.06 |
| **Vitamin A**^4^ | mcg | 400 | 400 | 3.6 |
| **Vitamin D** | mcg | 10 | 20 | 0.0 |
| **Vitamin E** | mg | 5.2 | 10 | 0.5 |
| **Vitamin K** | mcg | 20 | 26 | 1.4 |
| **Vitamin B1** | mg | 0.6 | 1.0 | 0.04 |
| **Vitamin B2** | mg | 0.8 | 1.2 | 0.04 |
| **Vitamin C** | mg | 60 | 60 | 0.9 |
| **Vitamin B6** | mg | 0.6 | 1.2 | 0.02 |
| **Vitamin B12** | mcg | 1.0 | 2.0 | 0.04 |
| **Folic acid** | mcg | 160 | 320 | 7 |
| **Niacin** | mg | 8 | 16 | 0.3 |
| **Pantothenic acid** | mg | 3.6 | 5 | 0.08 |
| **Biotin** | mcg | 12 | 20 | 0.9 |

^1^ Previous studies used 1.5xRDA for 12 months with no adverse effects. (52)

^2^ As there are concerns regarding high level of intake of iron in malaria endemic areas (30) lower levels than 2xRDA were chosen. A recent Cochrane Review on iron supplementation in malaria endemic areas, however, provides some evidence that iron may not increase the risk of clinical malaria or death when regular malaria surveillance and treatment services are provided. (53)

^3^ Zinc levels were chosen above 2xRDA/RNI in concordance with other trials, showing some positive effect on morbidity from diarrhoeal and respiratory disease with little evidence of any adverse effects even above the UL. (54)

^4^ Vitamin A levels were chosen at less than 2xRDA/RNI. Children are already receiving Vitamin A at various time points according to Gambian national guidelines and there may be an adverse effect associated with higher levels of Vitamin A supplementation. (55)

Table 4: *Dietary Reference Intakes (DRI) (Recommended Daily Allowance (RDA) & Adequate intake (AI)) (49), Reference Nutrient Intakes (RNI) WHO (56) and Reference Nutrient Intakes (RNI) UK (47) and Upper safe limits (UL) (49)of each micronutrient given according to age.*

|  |  | **RNI WHO** | **RNI UK** | **DRI (RDA/AI)** | **UL** | **RNI WHO** | **RNI UK** | **DRI (RDA/AI)** | **RNI WHO** | **RNI UK** | **DRI (RDA/AI)** | **UL** |
| --- | --- | --- | --- | --- | --- | --- | --- | --- | --- | --- | --- | --- |
|  |  | Infant |  |  |  | Children |  |  |  |  |  |  |
|  | Unit | ***7-12 months*** |  |  |  | ***1-3 years*** |  |  | ***4-6 years*** |  |  |  |
| **Calcium** | mg | 400 | 525 | 270 | ND | 500 | 350 | 500 | 600 | 450 | 800 | 2,500 |
| **Phosphorus** | mg | NS | 400 | 275 | ND | NS | 270 | 460 | NS | 350 | 500 | 3,000 |
| **Potassium** | mg | NS | 700 | 700 | ND | NS | 800 | 3000 | NS | 1100 | 3800 | ND |
| **Magnesium** | mg | 54 | 80 | 75 | ND | 60 | 85 | 80 | 76 | 120 | 130 | 65 |
| **Zinc** | mg | 4.1 | 5 | 3 | 5 | 4.1 | 5 | 3 | 4.8 | 6.5 | 5 | 7 |
| **Copper** | mg | NS | 0.3 | 0.22 | ND | NS | 0.4 | 0.34 | NS | 0.6 | 0.44 | 1 |
| **Iron** | mg | 9.3 | 7.8 | 11 | 40 | 5.8 | 6.9 | 7 | 6.3 | 6.1 | 10 | 40 |
| **Iodine** | mcg | 90 | 60 | 130 | ND | 90 | 70 | 90 | 90 | 100 | 90 | 200 |
| **Selenium** | mcg | 10 | 10 | 20 | 60 | 17 | 15 | 20 | 22 | 20 | 30 | 90 |
| **Manganese** | mg | 0.02 | NS | 0.6 | ND | 0.02 | NS | 1.2 |  | NS | 1.5 | 2 |
| **Vitamin A** | mg | 0.4 | 0.35 | 0.5 | 0.6 | 0.4 | 0.4 | 0.3 | 0.45 | 0.4 | 0.4 | 0.6 |
| **Vitamin C** | mg | 30 | 25 | 50 | ND | 30 | 30 | 15 | 30 | 30 | 25 | 400 |
| **Vitamin B1** | mg | 0.3 | 0.3 | 0.3 | ND | 0.5 | 0.5 | 0.5 | 0.6 | 0.7 | 0.6 | ND |
| **Vitamin B2** | mg | 0.4 | 0.4 | 0.4 | ND | 0.5 | 0.6 | 0.5 | 0.6 | 0.8 | 0.6 | ND |
| **Vitamin B6** | mg | 0.3 | 0.4 | 0.3 | ND | 0.5 | 0.7 | 0.5 | 0.6 | 0.9 | 0.6 | 30 |
| **Vitamin B12** | mcg | 0.7 | 0.4 | 0.5 | ND | 0.9 | 0.5 | 0.9 | 1.2 | 0.8 | 1.2 | ND |
| **Folic acid** | mcg | 80 | 50 | 80 | ND | 150 | 70 | 150 | 200 | 100 | 200 | 300 |
| **Pantothenic acid** | mg | 1.8 | 1.7 | 1.8 | ND | 2 | NS | 2 | 3 | NS | 3 | ND |
| **Niacin** | mg | 4 | 5 | 4 | ND | 6 | 8 | 6 | 8 | 11 | 8 | 10 |
| **Biotin** | mcg | 6 | NS | 6 | ND | 8 | NS | 8 | 12 | NS | 12 | ND |
| **Vitamin D** | mcg | 5 | 7 | 5 | 25 | 5 | 7 | 5 | 5 | NS | 5 | 50 |
| **Vitamin E** | mg | 2.7 | 2.7 | 5 | ND | 5 | NS | 6 | 5 | NS | 7 | 200 |
| **Vitamin K** | mcg | 10 | NS | 2.5 | ND | 15 | NS | 30 | 20 | NS | 55 | ND |

NS Not specified, ND Not determined

Table 5: *Data Entry Flow Chart during MMC Trial at Keneba Field station.*

| **Type of patient** | **Reception** | **Anthropometry** | **Triage** | **Doctor** | **Study Personnel (Fieldworker/Study Nurse) Tasks** |
| --- | --- | --- | --- | --- | --- |
| **New presentation** | Pick up notes  Issue of WK card | Yes | Yes | For all children between 6m and 5y a note appears on action page:  “This patient may be eligible for the MMC Trial. Please fill an ELIGIBILITY FORM.”  Doctor fills out eligibility form and sends patient to see the MMCT Study Personnel | - Take patient to pharmacy to receive medication - Check eligibility form - Consent - Enter information on eligibility, consent, enrolment and supplementation into the computer in the research room. - Will be given by computer: Study Number, Randomisation status, whether infant or child and whether assigned to haematological and micronutrient status subgroup - Give 2 weeks supplementation to carer - Write MMCT study number on back of WK card and put MMCT sticker on clinical notes - Take subject to reception for medical summary print out. - Collect print out of:   1. Cover for CRF   2. Day 1 summary   3. Field Follow-up forms for Week 1, Week 2, 4, 6, 8, 10, 12   4. Study Conclusion form   5. Pre-printed AE/SAE forms   6. Pre-printed Spot Check Forms - Distribute Field Follow-up form for Week 1 - Create a CRF with the print outs - Sign the completed printed CRF form for Day 1 |
| **New with severe illness** | As above but likely to be taken straight to Triage room | Yes | Yes | Severe illness  Referral page (alert message as above)  Doctor fills out eligibility form and hands form to SP | - Print out a weekly list of all eligible patients referred and send copy to SP at the coast for follow-up   On return to Keneba: (See above)   - Consenting/Enrolment - Supplementation - CRF |
| **On supplement, self referred or referred by study personnel** | Pick up notes with MMCT sticker on it | Yes | Yes | Alert message for Doctor:  ‘This patient is receiving MMCT supplementation – please record AE/SAE as appropriate’  Doctor fills out AE/SAE form | - Enter AE/SAE into MMCT AE/SAE logbook and CRF and inform PI/deputy who will send details of any SAE to the DSM |
| **Scheduled visits as part of MMCT:** |  |  |  |  |  |
| **6 weeks** | Pick up notes. Entered as ‘Research’  Alert message: “MMCT Follow-Up” | Yes | No | None | - SP will complete Compliance/ Morbidity form and enter the information into the computer - SP prints out completed week 6 CRF form and signs it and puts form into the CRF |
| **12 weeks** | Pick up notes. Entered as ‘Research’  Alert message: “MMCT Follow-up” | Yes | No | None | - SP will complete Compliance/ Morbidity form and enter the information into the computer - SP will organize blood sampling if appropriate - Laboratory Staff enter information into the clinical database - SP prints out completed week 12 CRF form and signs it and puts form into the CRF |
| **24 weeks** | Pick up notes.  Enter as ‘Research’  Alert message: “MMCT Follow-up” | Yes | No | None | - SP will complete Compliance/Morbidity form and enter the information into the computer - SP prints out completed week 24 CRF form and signs it and puts form into the CRF |
| **Post supplementation self-referred visit** | Pick up notes.  Normally enters clinic database | Yes | Yes | Yes  Doctors alert message:  “Patient has already been on MMCT supplementation” | - None - Data of clinic visits will be automatically recorded |

REFERENCES

1. Sheeran J. The challenge of hunger. Lancet. 2008 Jan 19;371(9608):180-1.

2. UN. The Millenium Development Goals Report. 2007.

3. Black RE, Allen LH, Bhutta ZA, Caulfield LE, de Onis M, Ezzati M, et al. Maternal and child undernutrition: global and regional exposures and health consequences. Lancet. 2008 Jan 19;371(9608):243-60.

4. Prentice AM, Gershwin ME, Schaible UE, Keusch GT, Victora CG, Gordon JI. New challenges in studying nutrition-disease interactions in the developing world. J Clin Invest. 2008 Apr;118(4):1322-9.

5. Man WD, Weber M, Palmer A, Schneider G, Wadda R, Jaffar S, et al. Nutritional status of children admitted to hospital with different diseases and its relationship to outcome in The Gambia, West Africa. Trop Med Int Health. 1998 Aug;3(8):678-86.

6. Scrimshaw NS, SanGiovanni JP. Synergism of nutrition, infection, and immunity: an overview. Am J Clin Nutr. 1997 Aug;66(2):464S-77S.

7. Bhutta ZA, Ahmed T, Black RE, Cousens S, Dewey K, Giugliani E, et al. What works? Interventions for maternal and child undernutrition and survival. Lancet. 2008 Feb 2;371(9610):417-40.

8. Hussey GD, Klein M. Routine high-dose vitamin A therapy for children hospitalized with measles. J Trop Pediatr. 1993 Dec;39(6):342-5.

9. Masoodpoor N. Impact of zinc supplementation on respiratory and gastrointestinal infections: A double-blind, randomized trial among urban Iranian schoolchildren. Paediatrics. 2008;121:S153-S4.

10. Baqui AH, Black RE, El Arifeen S, Yunus M, Chakraborty J, Ahmed S, et al. Effect of zinc supplementation started during diarrhoea on morbidity and mortality in Bangladeshi children: community randomised trial. BMJ. 2002 Nov 9;325(7372):1059.

11. Ruel MT, Rivera JA, Santizo MC, Lonnerdal B, Brown KH. Impact of zinc supplementation on morbidity from diarrhea and respiratory infections among rural Guatemalan children. Pediatrics. 1997 Jun;99(6):808-13.

12. Schaible UE, Kaufmann SH. Malnutrition and infection: complex mechanisms and global impacts. PLoS Med. 2007 May;4(5):e115.

13. Dewey KG, Adu-Afarwuah S. Systematic review of the efficacy and effectiveness of complementary feeding interventions in developing countries. Matern Child Nutr. 2008 Apr;4 Suppl 1:24-85.

14. Lartey A, Manu A, Brown KH, Peerson JM, Dewey KG. A randomized, community-based trial of the effects of improved, centrally processed complementary foods on growth and micronutrient status of Ghanaian infants from 6 to 12 mo of age. Am J Clin Nutr. 1999 Sep;70(3):391-404.

15. WHO. Salt as a vehicle for fortification. WHO Expert Consultation. 2008.

16. Latham MC, Ash DM, Makola D, Tatala SR, Ndossi GD, Mehansho H. Efficacy trials of a micronutrient dietary supplement in schoolchildren and pregnant women in Tanzania. Food Nutr Bull. 2003 Dec;24(4 Suppl):S120-8.

17. Shankar AH, Jahari AB, Sebayang SK, Aditiawarman, Apriatni M, Harefa B, et al. Effect of maternal multiple micronutrient supplementation on fetal loss and infant death in Indonesia: a double-blind cluster-randomised trial. Lancet. 2008 Jan 19;371(9608):215-27.

18. Ashworth A. Efficacy and effectiveness of community-based treatment of severe malnutrition. Food Nutr Bull. 2006 Sep;27(3 Suppl):S24-48.

19. Linneman Z, Matilsky D, Ndekha M, Manary MJ, Maleta K. A large-scale operational study of home-based therapy with ready-to-use therapeutic food in childhood malnutrition in Malawi. Matern Child Nutr. 2007 Jul;3(3):206-15.

20. Ciliberto MA, Sandige H, Ndekha MJ, Ashorn P, Briend A, Ciliberto HM, et al. Comparison of home-based therapy with ready-to-use therapeutic food with standard therapy in the treatment of malnourished Malawian children: a controlled, clinical effectiveness trial. Am J Clin Nutr. 2005 Apr;81(4):864-70.

21. Ndekha MJ, Manary MJ, Ashorn P, Briend A. Home-based therapy with ready-to-use therapeutic food is of benefit to malnourished, HIV-infected Malawian children. Acta Paediatr. 2005 Feb;94(2):222-5.

22. Adu-Afarwuah S, Lartey A, Brown KH, Zlotkin S, Briend A, Dewey KG. Randomized comparison of 3 types of micronutrient supplements for home fortification of complementary foods in Ghana: effects on growth and motor development. Am J Clin Nutr. 2007 Aug;86(2):412-20.

23. Kuusipalo H, Maleta K, Briend A, Manary M, Ashorn P. Growth and change in blood haemoglobin concentration among underweight Malawian infants receiving fortified spreads for 12 weeks: a preliminary trial. J Pediatr Gastroenterol Nutr. 2006 Oct;43(4):525-32.

24. Maleta K, Kuittinen J, Duggan MB, Briend A, Manary M, Wales J, et al. Supplementary feeding of underweight, stunted Malawian children with a ready-to-use food. J Pediatr Gastroenterol Nutr. 2004 Feb;38(2):152-8.

25. Heird WC, Lapillonne A. The role of essential fatty acids in development. Annu Rev Nutr. 2005;25:549-71.

26. Auestad N, Scott DT, Janowsky JS, Jacobsen C, Carroll RE, Montalto MB, et al. Visual, cognitive, and language assessments at 39 months: a follow-up study of children fed formulas containing long-chain polyunsaturated fatty acids to 1 year of age. Pediatrics. 2003 Sep;112(3 Pt 1):e177-83.

27. Hadders-Algra M, Bouwstra H, van Goor SA, Dijck-Brouwer DA, Muskiet FA. Prenatal and early postnatal fatty acid status and neurodevelopmental outcome. J Perinat Med. 2007;35 Suppl 1:S28-34.

28. Eilander A, Hundscheid DC, Osendarp SJ, Transler C, Zock PL. Effects of n-3 long chain polyunsaturated fatty acid supplementation on visual and cognitive development throughout childhood: a review of human studies. Prostaglandins Leukot Essent Fatty Acids. 2007 Apr;76(4):189-203.

29. Sazawal S, Black RE, Ramsan M, Chwaya HM, Stoltzfus RJ, Dutta A, et al. Effects of routine prophylactic supplementation with iron and folic acid on admission to hospital and mortality in preschool children in a high malaria transmission setting: community-based, randomised, placebo-controlled trial. Lancet. 2006 Jan 14;367(9505):133-43.

30. WHO/UNICEF. Iron supplementation of young children in regions where malaria trasnmission is intense and infectious disease highly prevalent. Geneva: Joint WHO/UNICEF Statement. 2007.

31. Nwolisa CE, Erinaugha EU, Ofoleta SI. Prescribing practices of doctors attending to under fives in a children's outpatient clinic in Owerri, Nigeria. J Trop Pediatr. 2006 Jun;52(3):197-200.

32. Cho HJ, Hong SJ, Park S. Knowledge and beliefs of primary care physicians, pharmacists, and parents on antibiotic use for the pediatric common cold. Soc Sci Med. 2004 Feb;58(3):623-9.

33. WHO. Hospital care for children. Guidelines for the management of common illnesses with limited resources. 2005.

34. Tsang R, Lucas A, Uauy R, Zlotkin S. Nutritional Needs of the Preterm Infant: Scientific Basis and Practical Guidelines.: Baltimore: Williams and Wilkins.; 1993.

35. MRC Guidelines for Good Clinical Practice in Clinical Trials. London: Medical Research Council; 1998.

36. MRC. Good Research Practice. MRC Ethics Series. 2005.

37. Brooks WA, Yunus M, Santosham M, Wahed MA, Nahar K, Yeasmin S, et al. Zinc for severe pneumonia in very young children: double-blind placebo-controlled trial. Lancet. 2004 May 22;363(9422):1683-8.

38. WHO. Management of severe malaria. A practical handbook. Geneva: WHO; 2000.

39. WHO. Management of the child with a serious infection or severe malnutrition. Geneva: WHO; 2000.

40. WHO. Technical updates of the guidelines on the integrated management of childhood illness (IMCI). Geneva: WHO; 2005.

41. Health DoSf. The Standard Drug Treatment Manual. In: Welfare DoSfHaS, editor. Banjul, The Gambia; 2005.

42. Yan L, Prentice A, Dibba B, Jarjou LM, Stirling DM, Fairweather-Tait S. The effect of long-term calcium supplementation on indices of iron, zinc and magnesium status in lactating Gambian women. Br J Nutr. 1996 Dec;76(6):821-31.

43. Brown KH, Peerson JM, Lopez de Romana G, de Kanashiro HC, Black RE. Validity and epidemiology of reported poor appetite among Peruvian infants from a low-income, periurban community. Am J Clin Nutr. 1995 Jan;61(1):26-32.

44. Fulford AJ, Rayco-Solon P, Prentice AM. Statistical modelling of the seasonality of preterm delivery and intrauterine growth restriction in rural Gambia. Paediatr Perinat Epidemiol. 2006 May;20(3):251-9.

45. Editorial: Iron and resistance to infection. Lancet. 1974 Aug 10;2(7876):325-6.

46. Report of a joint FAO/WHO expert consultation. Human vitamin and mineral requirements. 2002.

47. (DoH) DoH. Report on Haelth and Social Subjects 41. Dietary Reference Values for Food Energy and Nutrients for the United Kingdom. 2007 2007.

48. Safe upper levels for vitamins and minerals. Expert group on vitamins and minerals. . 2003.

49. Dietary Reference Intakes: The Complete Set. 2005.

50. Doherty CP. Host-pathogen interactions: the role of iron. J Nutr. 2007 May;137(5):1341-4.

51. McDermid JM, Prentice AM. Iron and infection: effects of host iron status and the iron-regulatory genes haptoglobin and NRAMP1 (SLC11A1) on host-pathogen interactions in tuberculosis and HIV. Clin Sci (Lond). 2006 May;110(5):503-24.

52. Rivera JA, Gonzalez-Cossio T, Flores M, Romero M, Rivera M, Tellez-Rojo MM, et al. Multiple micronutrient supplementation increases the growth of Mexican infants. Am J Clin Nutr. 2001 Nov;74(5):657-63.

53. Ojukwu JU, Okebe JU, Yahav D, Paul M. Oral iron supplementation for preventing or treating anaemia among children in malaria-endemic areas. Cochrane Database Syst Rev. 2009(3):CD006589.

54. Brooks WA, Santosham M, Naheed A, Goswami D, Wahed MA, Diener-West M, et al. Effect of weekly zinc supplements on incidence of pneumonia and diarrhoea in children younger than 2 years in an urban, low-income population in Bangladesh: randomised controlled trial. Lancet. 2005 Sep 17-23;366(9490):999-1004.

55. Darboe MK, Thurnham DI, Morgan G, Adegbola RA, Secka O, Solon JA, et al. Effectiveness of an early supplementation scheme of high-dose vitamin A versus standard WHO protocol in Gambian mothers and infants: a randomised controlled trial. Lancet. 2007 Jun 23;369(9579):2088-96.

56. WHO/FAO. Vitamin and mineral requirements in human nutrition; 2004.

APPENDICES

| **Appendix Number** | **Title** |
| --- | --- |
| 1 | MRC Scientific Coordinating Committee Approvals (including amendments)  The Gambia Government / MRC Laboratories Joint Ethics Committee Approvals |
| 2 | MRC Scientific Coordinating Committee Approval for KEMReS  The Gambia Government / MRC Laboratories Joint Ethics Committee Approval for KEMReS |
| 3 | Consent Form |
| 4 | Subject Information Sheet |
| 5 | Eligibility Check Form |
| 6 | Case Report Form (CRF) |
| 7 | Adverse Event Form |
| 8 | Serious Adverse Event Initial Reporting Form |
| 9 | Serious Adverse Event Follow-Up Form |
| 10 | Protocol Deviation/Violation Report Form |

Appendix 1: SCC/Ethical approvals for main MMCT trial including amendments.

|  | *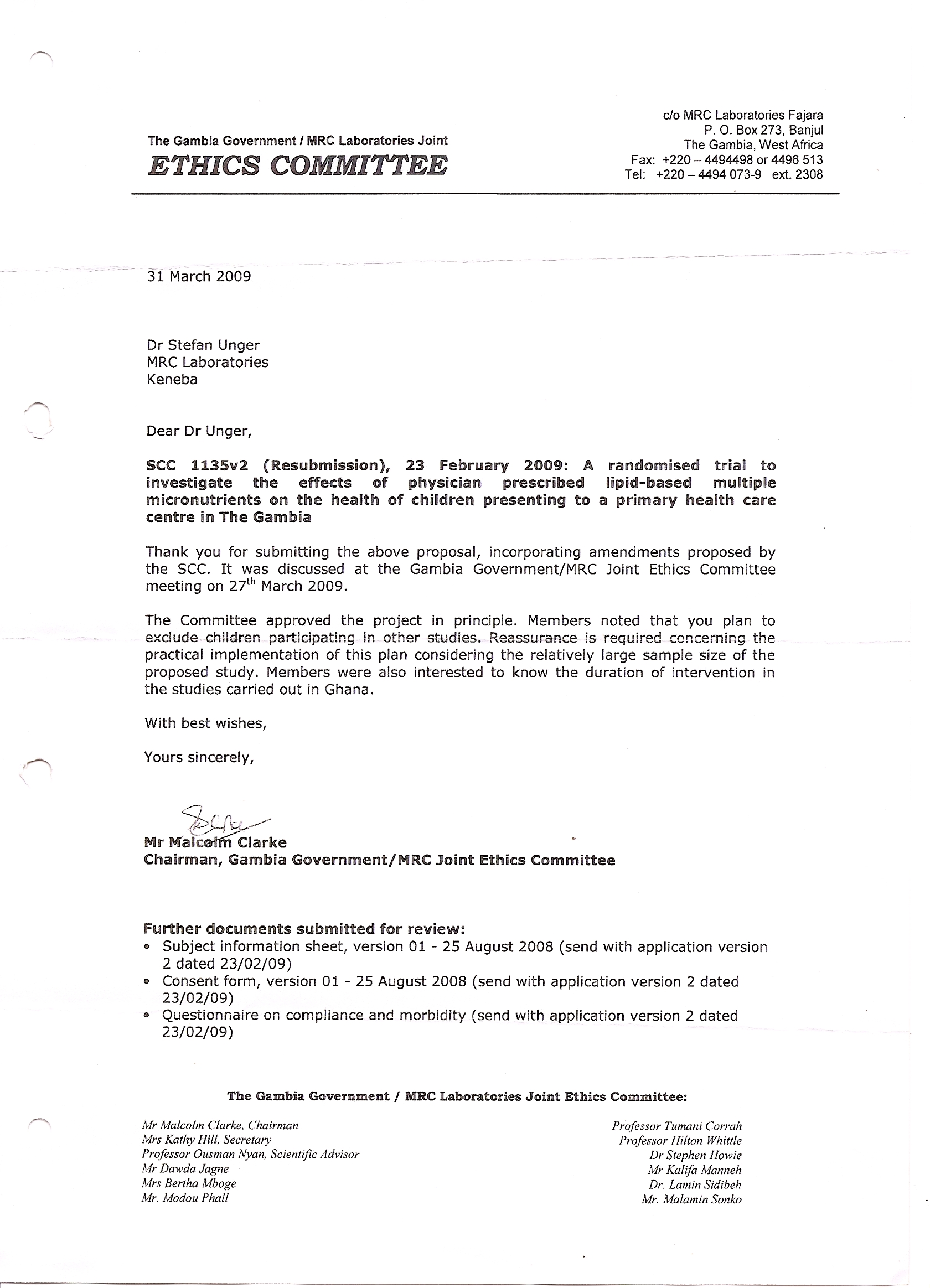* |
| --- | --- |
|  |  |
|  | *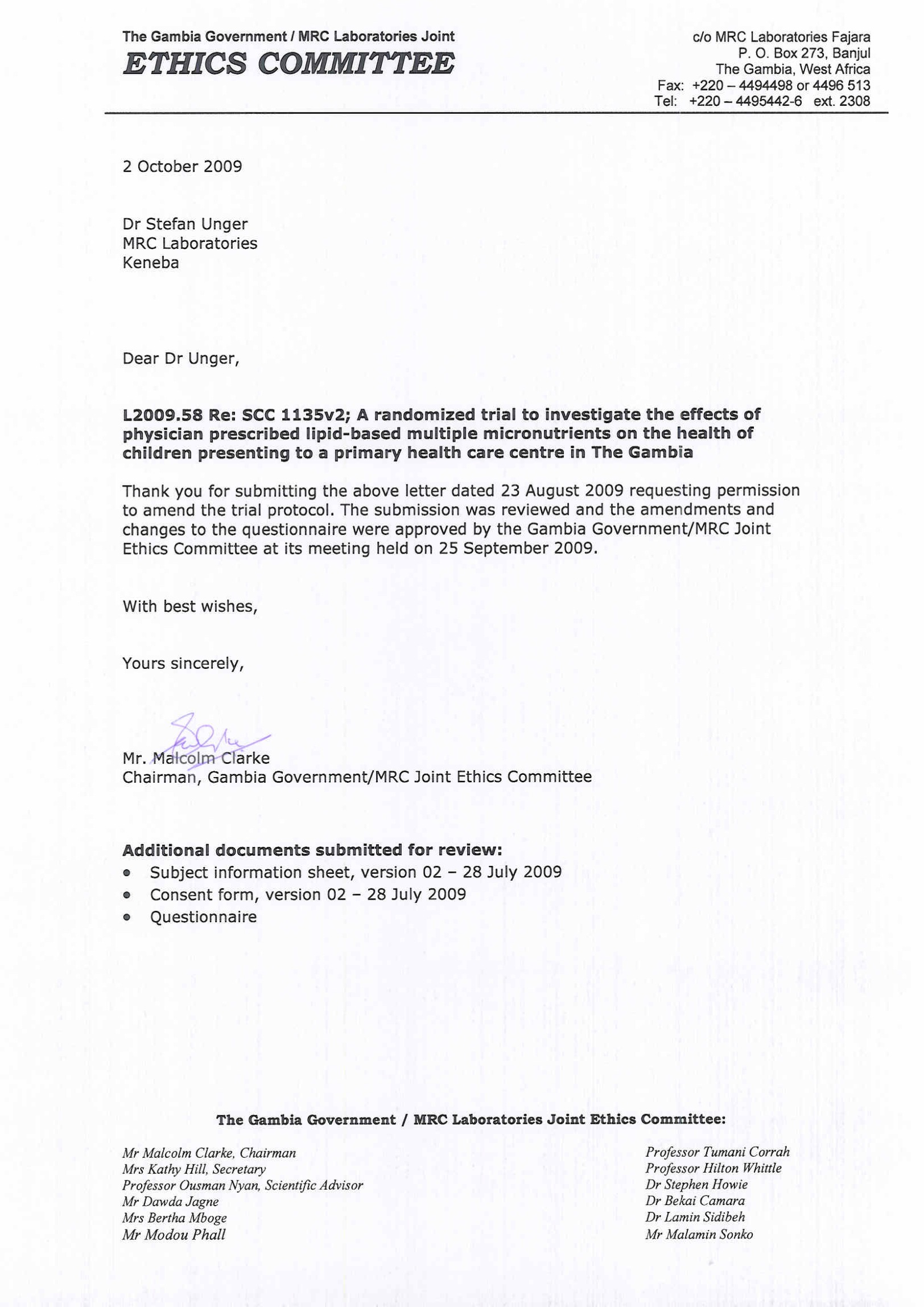* |

| *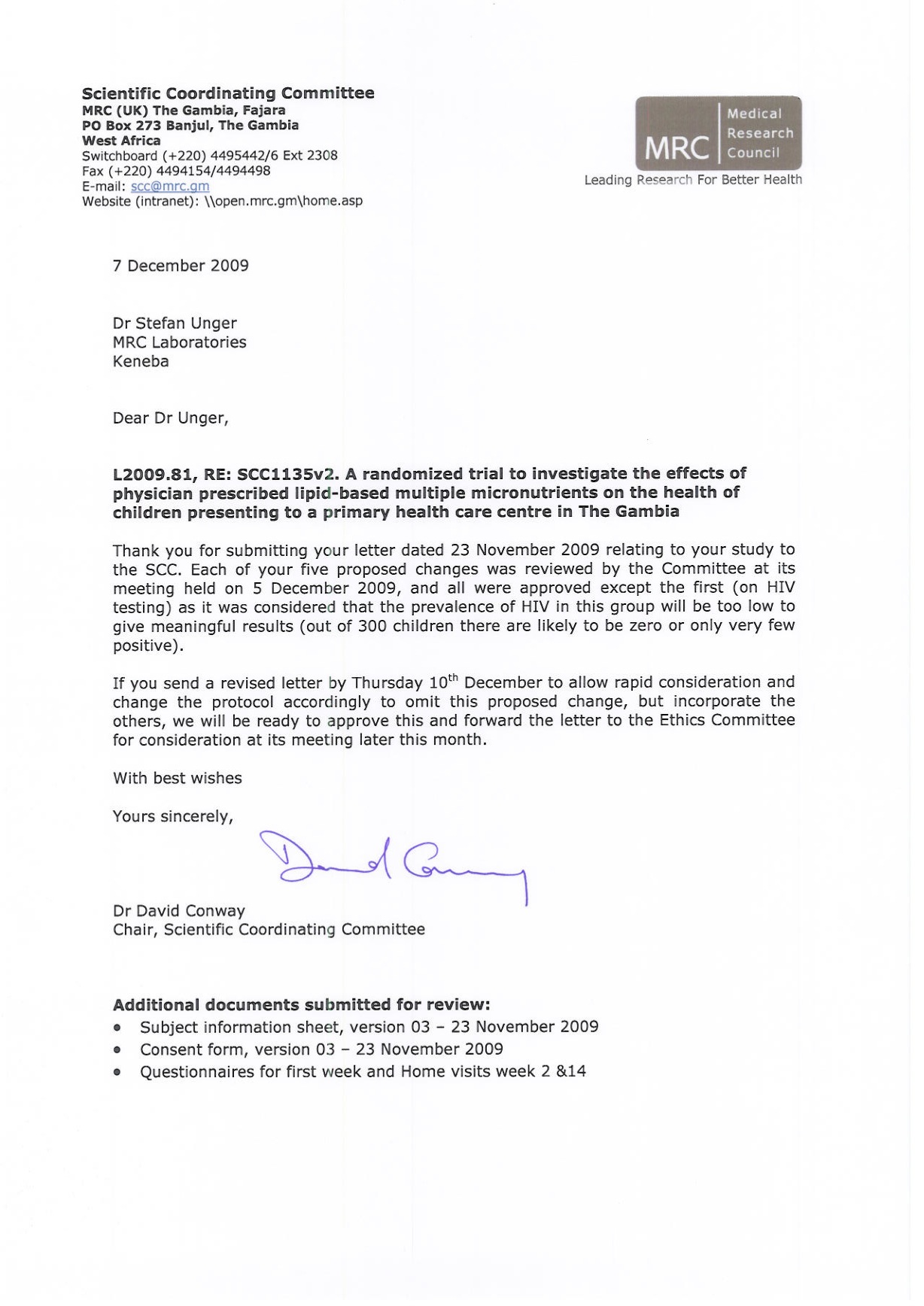* | *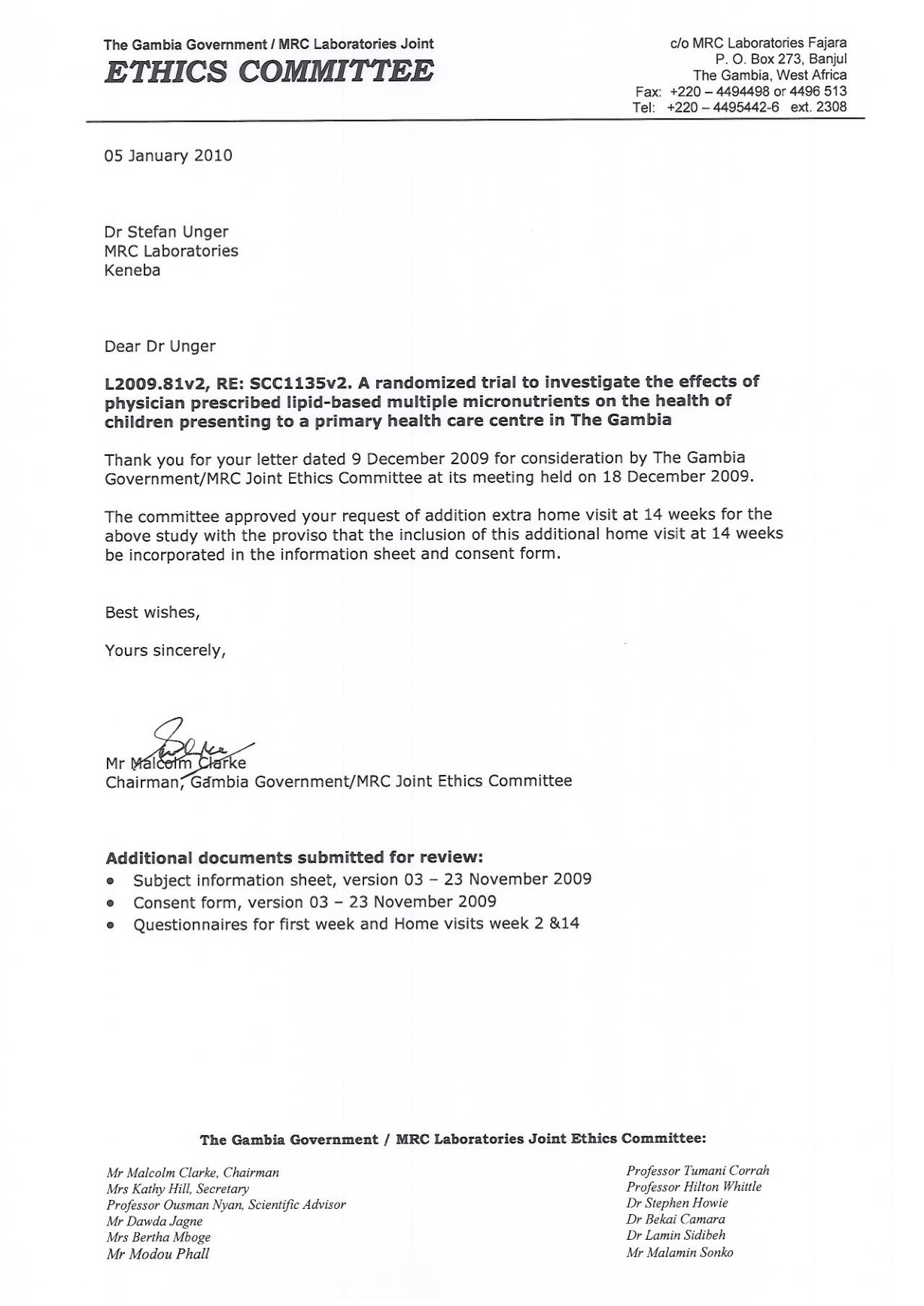* |
| --- | --- |

Appendix 2: SCC/Ethical approvals for KEMReS.

|  | 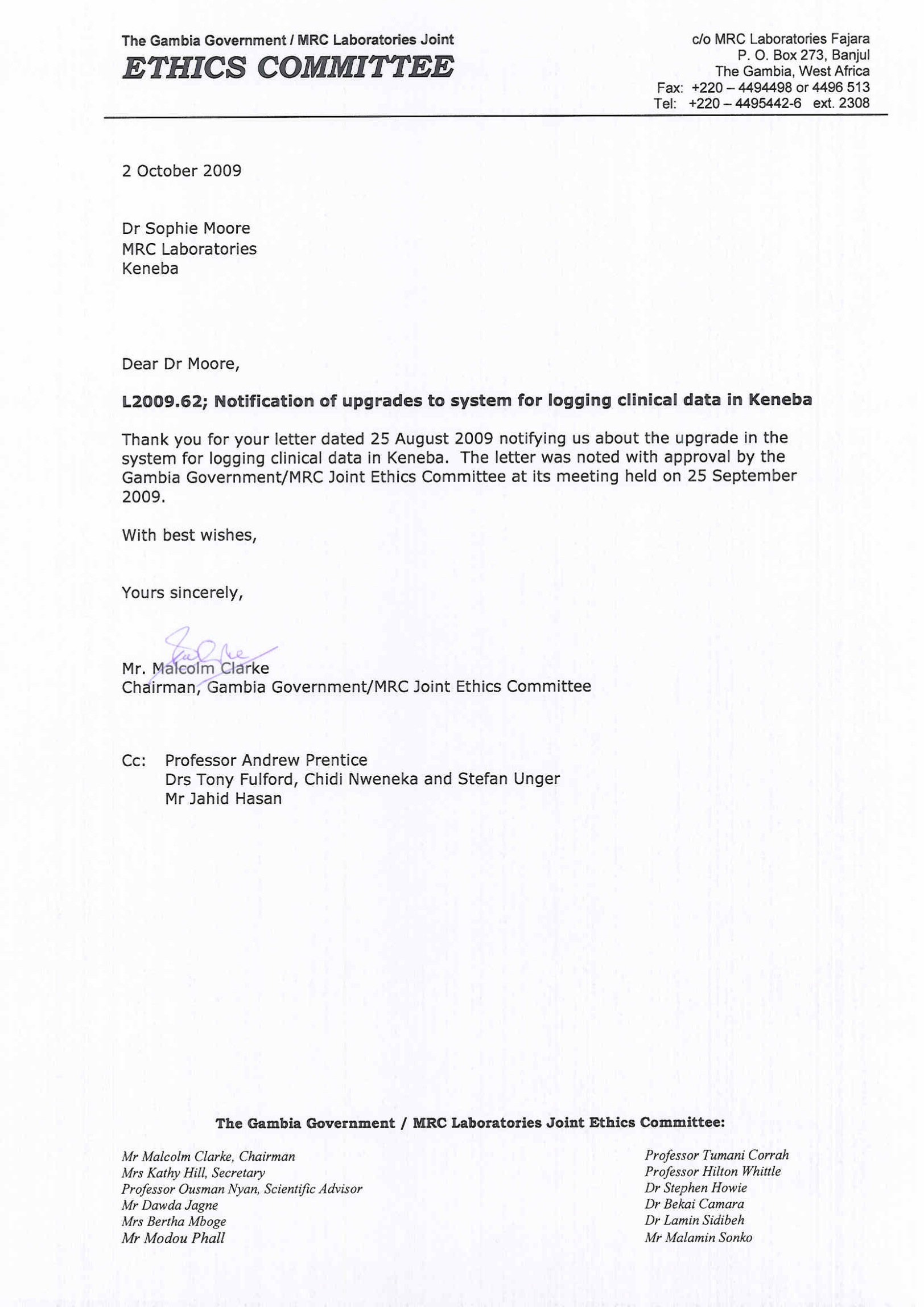 | | |
| --- | --- | --- | --- |
|  | |  |  |

Appendix 3: MMCT consent form.

Appendix 4: MMCT subject information sheet.

|  |  |
| --- | --- |

**Appendix 5: MMCT eligibility check form.**

Appendix 6: MMCT Case report form (CRF).

|  |  |
| --- | --- |
|  |  |
|  |  |
|  |  |
|  |  |
|  |  |
|  |  |
|  |  |
|  |  |
|  |  |

Appendix 7: MMCT Adverse event form.

**Appendix 8: MMCT Serious adverse event initial reporting form.**

**Appendix 9: MMCT Serious adverse event follow-up form.**

**Appendix 10: MMCT Protocol deviation/violation report form.**
