# Supplementary figures and images for: Impact of fortified versus unfortified lipid-based supplements on morbidity and nutritional status: A randomised double-blind placebo-controlled trial in ill Gambian children
Source: PLoS Med. 2017 Aug 15;14(8):e1002377. doi: 10.1371/journal.pmed.1002377 (PMC5557358; doi:10.1371/journal.pmed.1002377)

|  | *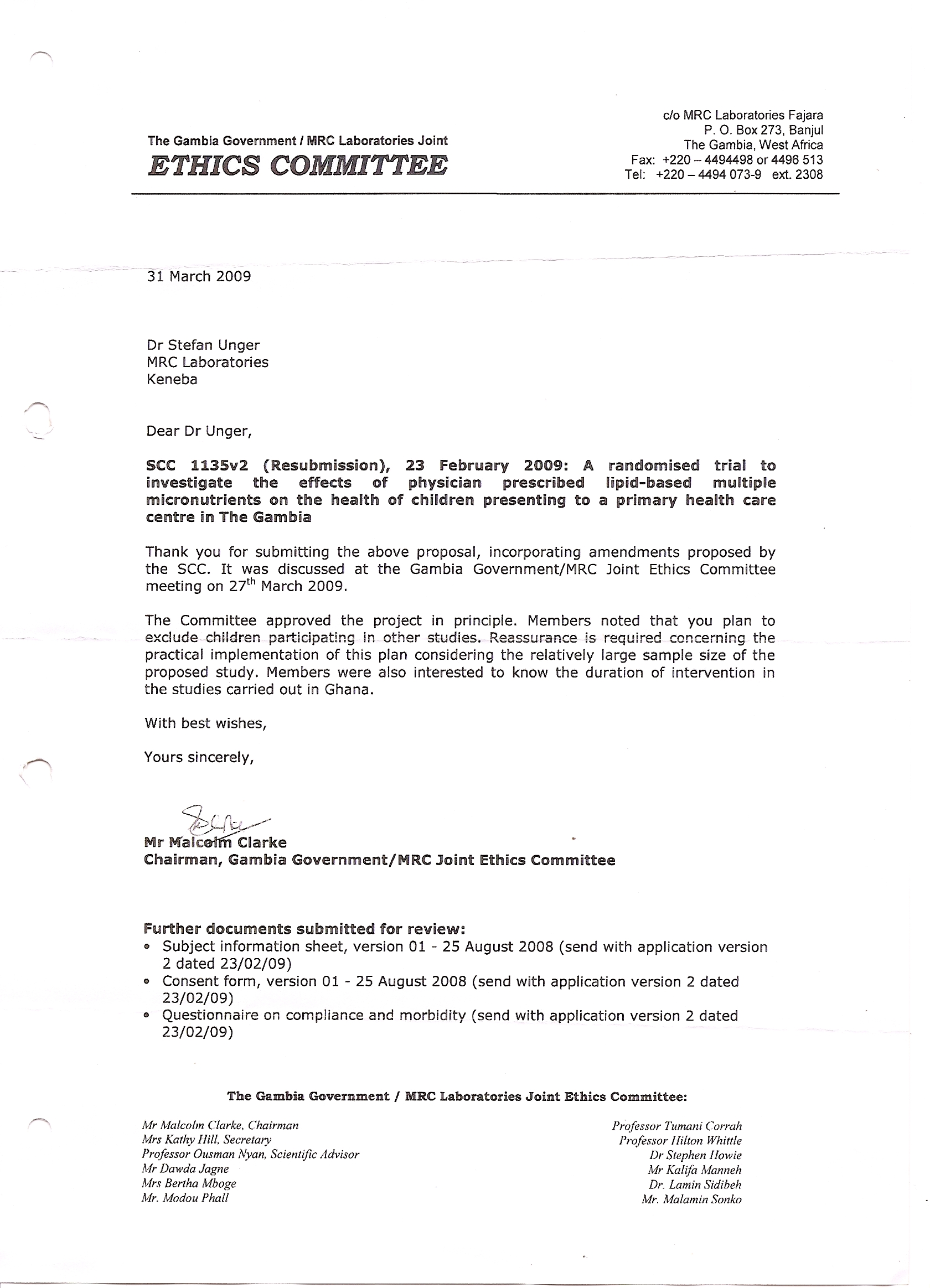* |
| --- | --- |
|  |  |
|  | *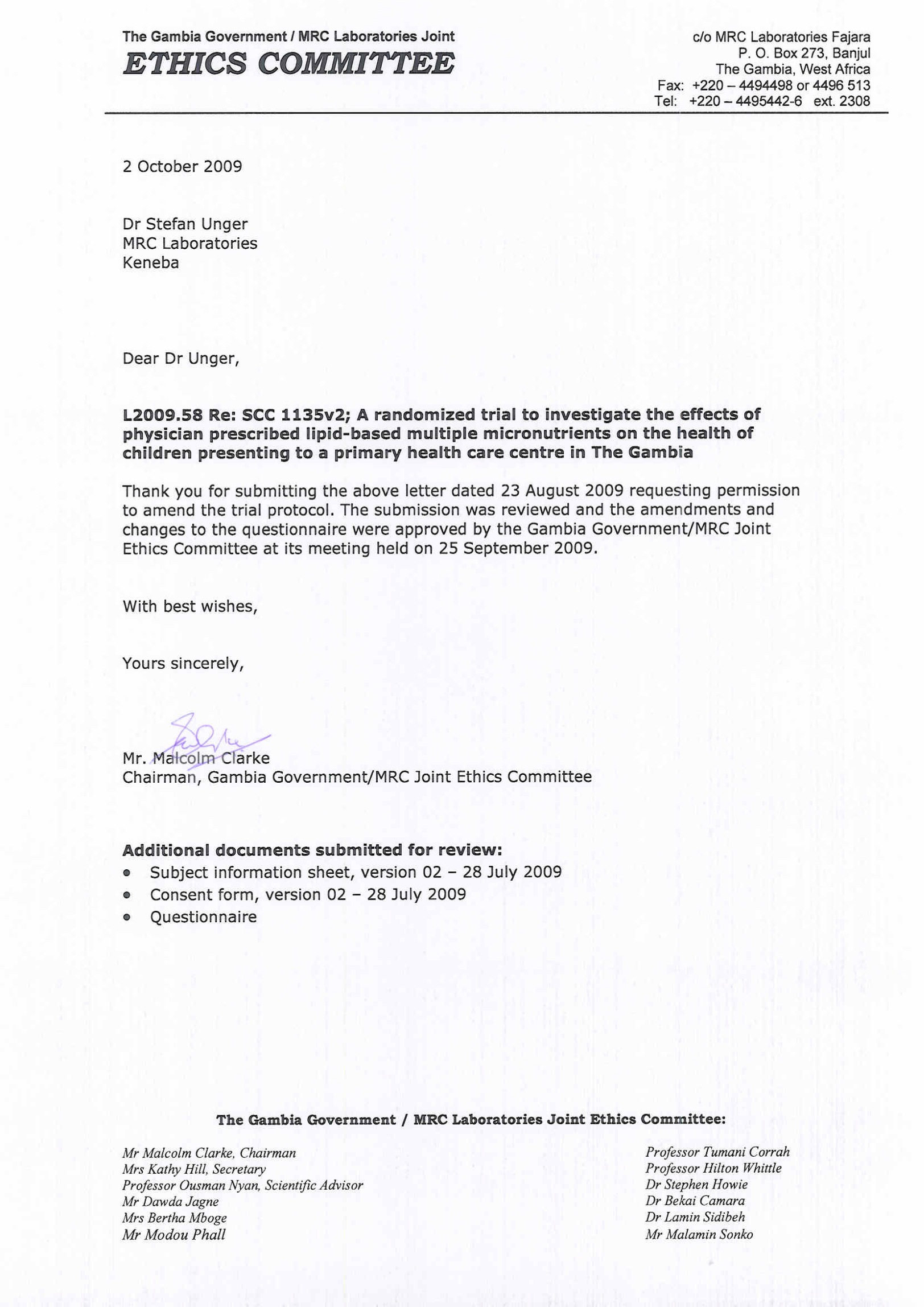* |

| *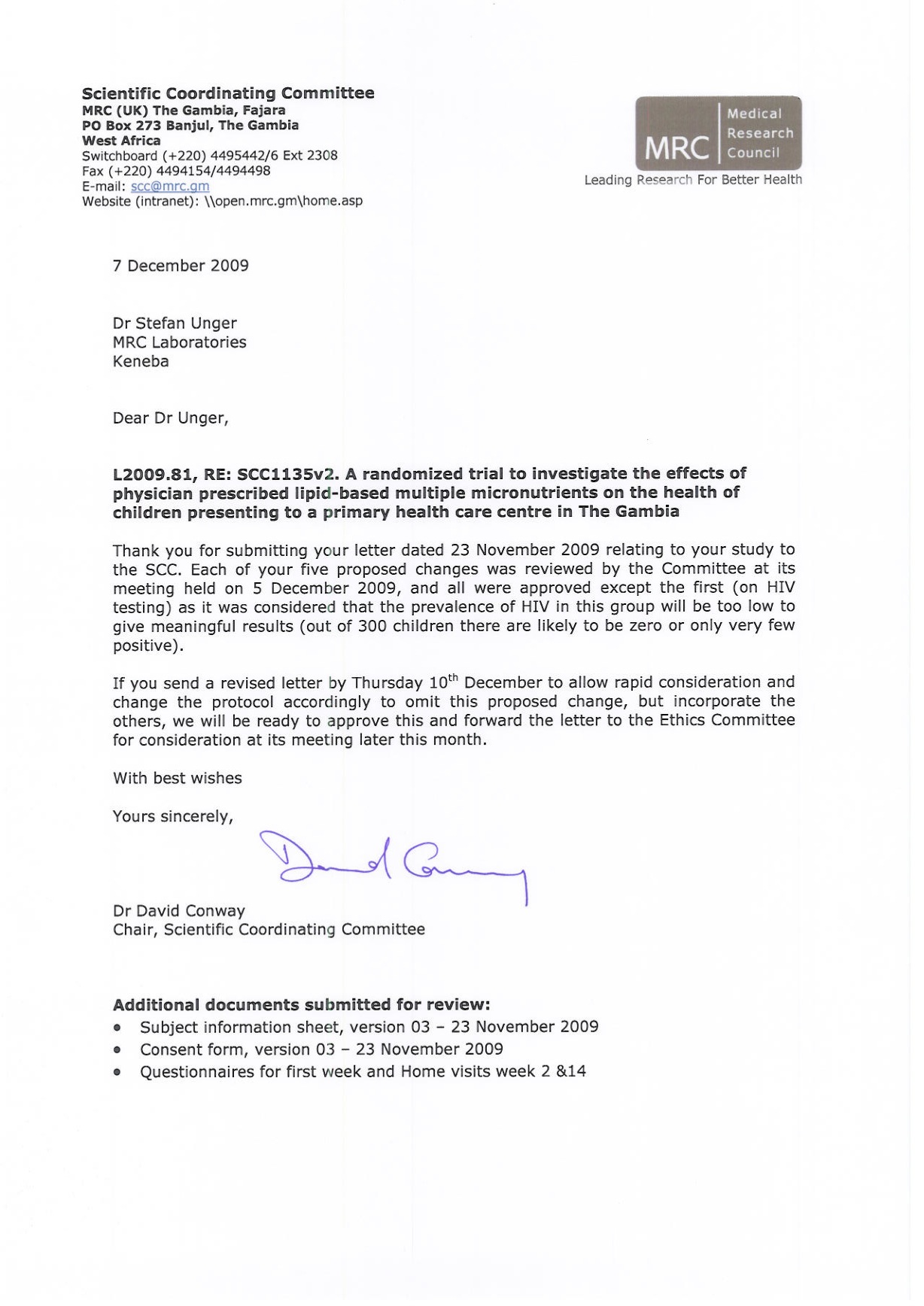* | *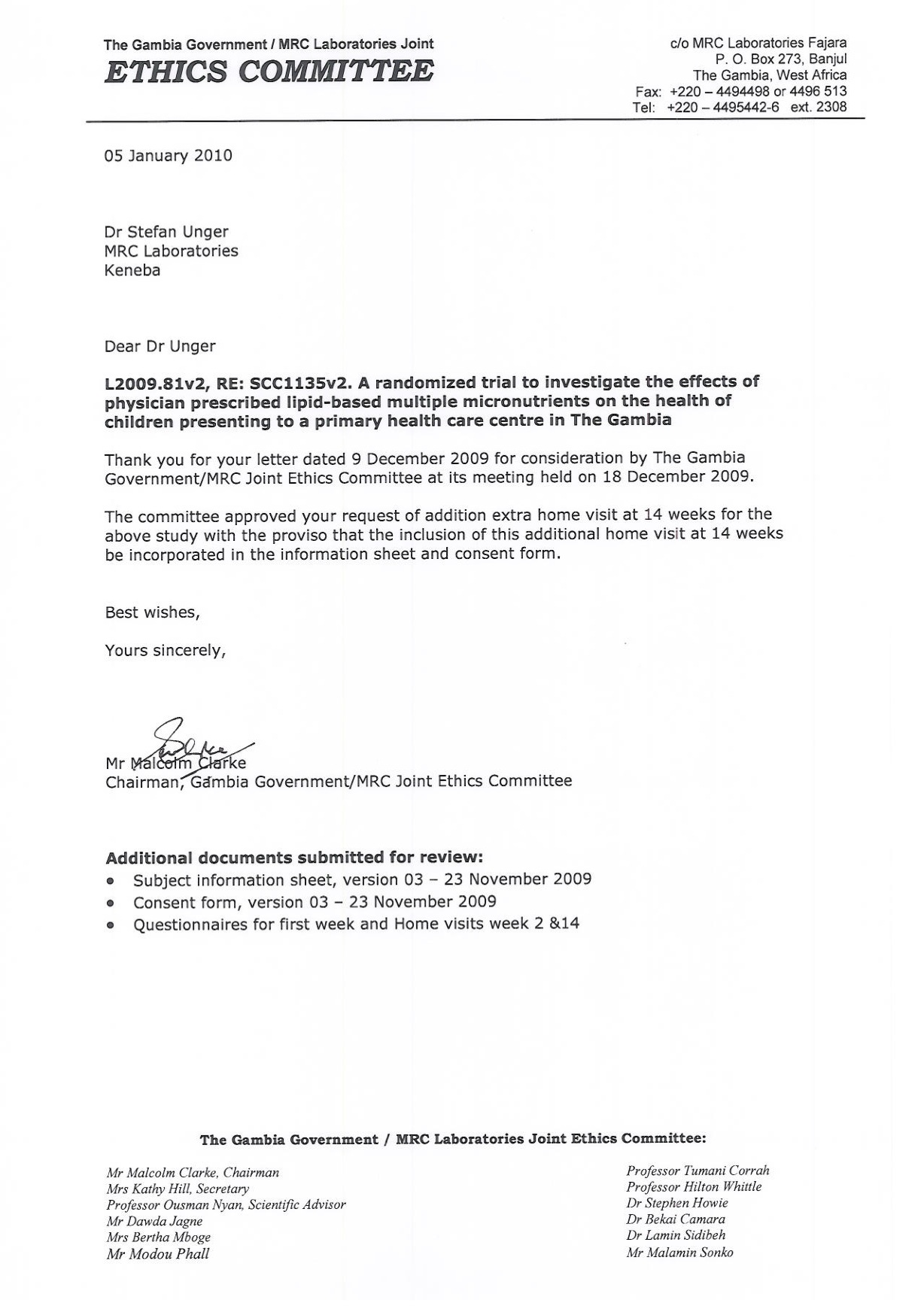* |
| --- | --- |

Supplement: S2 Text — (DOCX) [file pmed.1002377.s003.docx]
