# Supplementary material for: Impact of fortified versus unfortified lipid-based supplements on morbidity and nutritional status: A randomised double-blind placebo-controlled trial in ill Gambian children
Source: PLoS Med. 2017 Aug 15;14(8):e1002377. doi: 10.1371/journal.pmed.1002377 (PMC5557358; doi:10.1371/journal.pmed.1002377)
Supplement: S3 Text — (DOCX) [file pmed.1002377.s004.docx]

**S1 Appendix: Summary of literature review**

A Cochrane review [1] and updated systematic review [2] using pooled analysis of all included studies showed that micronutrient powders significantly reduced the prevalence of anaemia and iron-deficiency anaemia by 34% and 57% respectively but showed no overall significant benefit on linear growth and a significant increase in diarrhoea with non-significant tendency to increase acute respiratory infections.

**MMN powder**

Using the same search criteria (“micronutrient*” OR “multiple micronutrient” OR “multi-vitamin” OR “multi-mineral” OR “micronutrient powder” OR “MNP” OR “sprinkle” AND “Fortifi*” OR “food fortifi*” OR “point of use” OR “home fortification”) we searched OvidMedline, Embase and Cochrane library to include new studies published between 2013 and June 2017. We have identified 15 additional relevant studies using MMN plus zinc or iron. In line with previous findings MMN significantly reduce anaemia and iron deficiency. [3-5] However, effects on growth were variable with some studies showing small effects [6,7] and others none. [8-11] In children with intrathoracic tuberculosis MMN with or without zinc did not affect weight but did lead to a faster increase in height-for-age z-scores. [12]

The impact of MMN on morbidity was also variable. Some trials showed no benefits. [13,14] Self-reported MMN use in Kenya was associated with decreased hospitalizations for diarrhoea and fever with no effect on respiratory morbidity. [15] Two-week MNN supplementation after admission with an acute illness showed a reduction in the proportion of children with a new diarrhoea episode in Nigeria and new malaria episode in Uganda. [7,11] Of note home fortification in infants in Kenya showed an increase in reported days spent with cough and dyspnoea with MNN +iron (12.5mg) versus MNN without iron. [16]

**LNS with MMN**

We also conducted a literature review on randomised controlled trials testing the effect of lipid-based, RUSF or RUTF on growth and morbidity. We searched OvidMedline, Embase and Cochrane libraries without language limitation with the search criteria "RUSF" OR "RUTF" OR "LNS" OR "Lipid-based nutrient*” AND "child*" OR "infant*" AND "randomi*" OR "trial" from 2009 to June 2017. The search identified 19 studies (excluding the present study) of which 18 were placebo-controlled clinical trials with growth and/or morbidity as an outcome.

**LNS (fortification/small quantity LNS)**

Community LNS supplementation showed various effects on growth with some showing no effect, [17-20] some showing small or moderate increases in linear growth [21-27] and some showing moderate increase in weight. [22-24,26,28] SQ-LNS improved micronutrient status for folate and vitamin A [20] and reduced the prevalence of anaemia. [23] Only one SQ-LNS trial had morbidity as the main outcome (assessing safety) with no effect on malaria or respiratory morbidity observed. [20]

**RUSF (Ready-to-use supplementary foods/medium-quantity LNS)**

Four months supplementation with RUSF in Chad did not reduce cumulative incidence of wasting but had a small effect on gain in height-for-age (+0.03 z-scores/month, 95%CI: 0.01, 0.04; p<0.001), increased haemoglobin concentration (+3.8g/l, 95%CI 0.6, 7.0; p=0.02) and reduced self-reported diarrhoea and fever episodes. [29] Supplementation in 6 – 18 month children including 7mg iron and 7 mg zinc did not affect zinc or anaemia status but growth and morbidity were not studied. [30] RUSF for 100 days given to Haitian school-aged children increased their BMI. [31]

**RUTF (Ready-to-use therapeutic foods/large quantity LNS)**

One month supplementation of RUTF of non-malnourished children reduced decline in WHZ and incidence of wasting with no reduction in mortality. [32] Two week RUTF supplementation of children presenting with malaria was associated with faster weight gain. [33] Two week RUTF supplementation of children presenting with either malaria, diarrhoea and/or LRTI compared to no supplement showed no reduction in the incidence of malnutrition in Nigeria but a 33.3% reduction in initially non-malnourished children (definition: weight-for-height z-score >-2, MUAC >125mm) in Uganda. [7,11] Although not the primary outcome, morbidity was not affected by RUTF in the Uganda trial but a significant increase in incidence of diarrhoea was observed in Nigeria over a 24wk period.

Apart from the present trial no randomised double blind RCT was identified using small quantity LNS or MMN in children presenting unwell with morbidity as the main outcome. At the same time prescriptions of MMN is high in primary care and hospital outpatient clinics in SSA and no internationally accepted guidelines exist. [34]

**Implication of all the available evidence**

Inclusion of the findings from the present trial with the prior evidence highlights the heterogenous effect of MMN and LNS MMN supplementation on growth and morbidity with some evidence of potentially harmful effects. Blanket prescribing of MMN in primary health clinic in similar settings should be carefully considered particularly if containing iron.

**References**

1. De-Regil LM, Suchdev PS, Vist GE, Walleser S, Pena-Rosas JP. Home fortification of foods with multiple micronutrient powders for health and nutrition in children under two years of age. Cochrane Database Syst Rev 2011; 9: CD008959.
2. Soofi S, Cousens S, Iqbal SP, Akhund T, Khan J, Ahmed I et al. Effect of provision of daily zinc and iron with several micronutrients on growth and morbidity among young children in Pakistan: a cluster-randomised trial. Lancet 2013; 382(9886): 29-40.
3. Bilenko N, Fraser D, Vardy H, Belmaker I. Impact of multiple micronutrient supplementation ("sprinkles") on iron deficiency anemia in Bedouin Arab and Jewish infants. Israel Medical Association Journal: Imaj 2014; 16(7): 434-8.
4. Cardoso MA, Augusto RA, Bortolini GA, Oliverira CSM, Tietzman DC, Sequiera LAS et al. Effect of providing multiple micronutrients in powder through primary healthcare on anemia in young brazilian children: A multicentre pragmatic controlled trial. PLoS ONE 2016; 11 (3) (e0151097).
5. Osei AK, Pooja P, Spiro D, Denbdra A, Haelow N, Morais C et al. Adding multiple micronutrient powders to a homestead food production programme yields marginally significant benefit on anaemia reduction among young children in Nepal. (Special Issue: Policy, program and innovation in complementary feeding.). Maternal and Child Nutrition 2015; 11(s4): 188-202.
6. Locks L, Manji K, McDonald C, Kupka R, Kisenge R, Aboud S et al. Effect of Zinc & Multiple Micronutrient Supplements on Growth in Tanzanian Children. The FASEB Journal 2015; 29(1 Supplement).
7. van der Kam S, Roll S, Swarthout T, Edyegu-Otelu G, Matsumoto A, Kasujja FX et al. Effect of Short-Term Supplementation with Ready-to-Use Therapeutic Food or Micronutrients for Children after Illness for Prevention of Malnutrition: A Randomised Controlled Trial in Uganda. PLoS Medicine 2016; 13 (2) (e1001951).
8. Mda S, van Raaij JM, de Villiers FP, Kok FJ. Impact of multi-micronutrient supplementation on growth and morbidity of HIV-infected South African children. Nutrients 2013; 5(10): 4079-92.
9. Kupka R, Manji KP, Bosch RJ, Aboud S, Kisenge R, Okuma J et al. Multivitamin supplements have no effect on growth of Tanzanian children born to HIV-infected mothers. Journal of Nutrition 2013; 143(5): 722-7.
10. Hassanzadeh-Rostam Z, Kazemi A, Akhlaghi M. Effect of multivitamin-mineral supplements is transient in preschool children with low appetite and growth failure. ICAN: Infant, Child & Adolescent Nutrition 2014; 6(6): 345-50.
11. van der Kam S, Salse-Ubach N, Roll S, Swarthout T, Gayton-Toyoshima, S, Jiya NM et al. Effect of Short-Term Supplementation with Ready-to-Use Therapeutic Food or Micronutrients for Children after Illness for Prevention of Malnutrition: A Randomised Controlled Trial in Nigeria. PLoS Medicine 2016; 13 (2) (e1001952).
12. Lodha R, Mukherjee A, Singh V, Singh S, Friis H, Faurholt-Jepsen D et al. Effect of micronutrient supplementation on treatment outcomes in children with intrathoracic tuberculosis: a randomized controlled trial. Am J Clin Nutr 2014; 100(5): 1287-97.
13. McDonald CM, Manji KP, Kisenge R, Aboud S, Spiegelman D, Fawzi WW et al. Daily Zinc but Not Multivitamin Supplementation Reduces Diarrhea and Upper Respiratory Infections in Tanzanian Infants: A Randomized, Double-Blind, Placebo-Controlled Clinical Trial. Journal of Nutrition 2015; 145(9): 2153-60.
14. Zlotkin S, Newton S, Aimone AM, Azindow I, Amenga-Etego S, Tcum K et al. Effect of iron fortification on malaria incidence in infants and young children in Ghana: a randomized trial. Jama 2013; 310(9): 938-47.
15. Suchdev PS, Addo OY, Martorell R, Grant FKE, Ruth LJ, Patel MK et al. Effects of community-based sales of micronutrient powders on morbidity episodes in preschool children in Western Kenya. American Journal of Clinical Nutrition 2016; 103(3): 934-41.
16. Barth-Jaeggi T, Moretti D, Kvalsvig J, Holding PA, Njenga J, Mwangi A et al. In-home fortification with 2.5 mg iron as NaFeEDTA does not reduce anaemia but increases weight gain: a randomised controlled trial in Kenyan infants. (Special Issue: Policy, program and innovation in complementary feeding.). Maternal and Child Nutrition 2015; 11(s4): 151-62.
17. Ashorn P, Alho L, Ashorn U, Cheung YB, Dewey KG, Gondwe A et al. Supplementation of Maternal Diets during Pregnancy and for 6 Months Postpartum and Infant Diets Thereafter with Small-Quantity Lipid-Based Nutrient Supplements Does Not Promote Child Growth by 18 Months of Age in Rural Malawi: A Randomized Controlled Trial. J Nutr 2015; 145(6): 1345-53.
18. Maleta KM, Phuka J, Alho L, Cheung YB, Dewey KG, Ashorn U et al. Provision of 10-40 g/d Lipid-Based Nutrient Supplements from 6 to 18 Months of Age Does Not Prevent Linear Growth Faltering in Malawi. J Nutr 2015; 145(8): 1909-15.
19. Maleta K, Phuka J, Cheung YB, Thakwalakwa C, Dewey K, Manary M et al. Effect of complementary feeding with lipid-based nutrient supplements and corn-soy blend on the incidence of stunting and linear growth among 6- to 18-month-old infants and children in rural Malawi. Maternal and Child Nutrition 2015; 11: 132-43.
20. Mangani C, Ashorn P, Maleta K, Phuka J, Thakwalakwa C, Dewey K et al. Lipid-based nutrient supplements do not affect the risk of malaria or respiratory morbidity in 6-to 18-Month-Old Malawian Children in a randomized controlled trial. Journal of Nutrition 2014; 144(11): 1835-42.
21. Iannotti LL, Dulience SJL, Green J, Joseph S, Francois J, Antenor ML et al. Linear growth increased in young children in an urban slum of Haiti: A randomized controlled trial of a lipid-based nutrient supplement. American Journal of Clinical Nutrition 2014; 99(1): 198-208.
22. Thakwalakwa CM, Ashorn P, Jawati M, Phuka JC, Cheung YB, Maleta KM. An effectiveness trial showed lipid-based nutrient supplementation but not corn-soya blend offered a modest benefit in weight gain among 6- to 18-month-old underweight children in rural Malawi. Public health nutrition 2012; 15(9): 1755-62.
23. Hess SY, Abbeddou S, Jimenez EY, Some JW, Vosti SA, Quedraogo ZP et al. Small-quantity lipid-based nutrient supplements, regardless of their zinc content, increase growth and reduce the prevalence of stunting and Wasting in young burkinabe children: A cluster-randomized trial. PLoS ONE 2015; 10 (3) (0122242).
24. Adu-Afarwuah S, Lartey A, Brown KH, Zlotkin S, Briend A, Dewey KG. Randomized comparison of 3 types of micronutrient supplements for home fortification of complementary foods in Ghana: effects on growth and motor development. Am J Clin Nutr 2007; 86(2): 412-20.
25. Thakwalakwa C, Ashorn P, Phuka J, Cheung YB, Briend A, Puumalainen T et al. A lipid-based nutrient supplement but not corn-soy blend modestly increases weight gain among 6- to 18-month-old moderately underweight children in rural Malawi. J Nutr 2010; 140(11): 2008-13.
26. Adu-Afarwuah S, Lartey A, Okronipa H, Ashorn P, Peerson JM, Arimond M et al. Small-quantity, lipid-based nutrient supplements provided to women during pregnancy and 6 mo postpartum and to their infants from 6 mo of age increase the mean attained length of 18-mo-old children in semi-urban Ghana: a randomized controlled trial. Am J Clin Nutr 2016; 104:797-808.
27. Dewey KG, Mridha MK, Matias SL, Arnold CD, Cummins JR, Ali Kahn MS et al. Lipid-based nutrient supplementation in the first 1000 d improves child growth in Bangladesh: a cluster-randomized effectiveness trial. Am J Clin Nutr 2017; 105:944-957.
28. Ashorn P, Phuka J, Cheung YB, Briend A, Puumalainen T, Maleta K. A lipid-based nutrient supplement but not corn-soy blend modestly increases weight gain among 6- to 18-month-old moderately underweight children in Rural Malawi. Journal of Nutrition 2010; 140(11): 2008-13.
29. Huybregts L, Houngbe F, Salpeteur C, Brown R, Roberfroid D, Ait-Aissa M et al. The Effect of Adding Ready-to-Use Supplementary Food to a General Food Distribution on Child Nutritional Status and Morbidity: A Cluster-Randomized Controlled Trial. PLoS Med 2012; 9(9): e1001313.
30. Siega-Riz AM, Estrada Del Campo Y, Kinlaw A, Reinhart GA, Allen LH, Shahab-Ferdows S et al. Effect of supplementation with a lipid-based nutrient supplement on the micronutrient status of children aged 6-18 months living in the rural region of Intibuca, Honduras. Paediatric and perinatal epidemiology 2014; 28(3): 245-54.
31. Iannotti LL, Henretty NM, Delnatus JR, Previl W, Stehl T, Vorkoper S et al. Ready-to-use supplementary food increases fat mass and BMI in haitian school-aged children. Journal of Nutrition 2015; 145(4): 813-22.
32. Isanaka S, Nombela N, Djibo A, Poupard M, Van Beckhoven D, Gaboulaud V et al. Effect of preventive supplementation with ready-to-use therapeutic food on the nutritional status, mortality, and morbidity of children aged 6 to 60 months in Niger: A cluster randomized trial. JAMA - Journal of the American Medical Association 2009; 301(3): 277-85.
33. van der Kam S, Swarthout T, Niragira O, Froud A, Sompwe EM, Mills C et al. Ready-to-use therapeutic food for catch-up growth in children after an episode of Plasmodium falciparum malaria: an open randomised controlled trial. PloS one 2012; 7(4): e35006.
34. Risk R, Naismith H, Burnett A, Moore SE, Cham M, Unger S. Rational prescribing in paediatrics in a resource-limited setting. Archives of disease in childhood 2013; 98(7): 503-9.
