# Supplementary material for: Impact of fortified versus unfortified lipid-based supplements on morbidity and nutritional status: A randomised double-blind placebo-controlled trial in ill Gambian children
Source: PLoS Med. 2017 Aug 15;14(8):e1002377. doi: 10.1371/journal.pmed.1002377 (PMC5557358; doi:10.1371/journal.pmed.1002377)
Supplement: S4 Text — (DOCX) [file pmed.1002377.s005.docx]

**S2 Appendix:** Statistical analysis plan

(as agreed with the independent trial monitor and data safety monitor)

**Multiple Micronutrient Clinical Trial (MMCT)**

**(ISRCTN73571031)**

**MCR Keneba, The Gambia**

**Statistical analysis plan**

In order to assess the intervention effects on the two primary outcomes (A) clinic revisits and (B) growth, we will take the following steps (in sequential order):

# Description of baseline characteristics, by treatment group

First, the three intervention groups will be described in regards to the baseline characteristics. We will test the adequacy of randomization in our trial by testing for any group differences. We will control for any variables that show a significant difference at baseline in the analysis as they may lead to confounding if the variable in question is associated with the outcome.

Baseline data of each group will be described in a table (proportions and total or mean/median and SD as appropriate).

Baseline data will include:

1. Number recruited into each group
2. Age,
3. Sex,
4. Anthropometric indices (WHO-referenced z-scores) for:
   1. Weight for height
   2. Weight for age
   3. Height for age
   4. MUAC for age
   5. Skin fold thickness for age
5. Disease patterns at recruitment:
   1. Infection
      1. Respiratory infections
      2. Diarrhoeal disease
      3. Skin infections
      4. Malaria
      5. Others
   2. Nutrition related
   3. Injury
   4. Other
6. Distance to MRC Keneba health facility
7. Distance to nearest health facility
8. Location (whether village is on a main road, hence easier access to transport)
9. Family background including number of co-wives of mothers, number of paternal and maternal siblings, birth order of maternal siblings, age and education of the mother and whether child is living with the mother.

Differences between treatment groups will be analysed using three different ways according to type of data:

Continuous – oneway ANOVA analysis

Ordered categorical – Kruskal-Wallis oneway analysis

Categorical – Chi square analysis

We will also describe the seasonality of clinic visits to Keneba in general using a bar chart. We will include all 6m to 5 yr visits at MRC Keneba between December 2009 and December 2011.

In a CONSORT diagram we will include the number of the following:

- Assessed for eligibility
- Not meeting inclusion criteria
- Enrolled
- Wrongly recruited
- Prematurely terminated
- Withdrawn consent
- Loss to follow up
- Died
- Included for analysis

We will show the proportion of initial presentations fulfilling inclusion criteria and recruited of all presentations during the recruitment period of MMCT.

# Group descriptions for (primary) outcomes

Two separate primary outcomes are defined for this study to describe *Health Status*:

(A) Clinic return visits

(B) Growth

## Clinic return visits

The primary endpoint is clinic visits, defined as a presentation with one or more relevant (see section 2.1.2) diagnoses of an enrolled child to the Keneba primary health care clinic or a presentation to either of the two government run health care facilities in Kwinella and Karantaba.

### Clinic visits description

For each group, we will report the median (IQR) and mean (SD) with 95% confidence intervals over a 12-month follow up period (primary analysis) or 6-month period for the purpose of the thesis of Dr Stefan Unger).

In addition, we will report the number of visits with separate diagnoses and deaths (composite indicator) in each group.

We will also calculate the total number of visits and the percentage of these with diagnoses included in the primary analysis.

### Comments on revisit definitions

Following visits will be excluded from the analyses:

1. Routine Child Health Examinations *AND* “Well without complaint” *OR* “Well with complaint” as a diagnosis
2. Research follow up *AND* “Well without complaint” *OR* “Well with complaint” as the diagnosis
3. Presentation with an injury/poisoning including following ICD-10 codes used in KEMReS:
   1. Accidental poisoning (X49)
   2. Dog bite (W54)
   3. Fall (W19)
   4. Foreign body in eye (W44)
   5. Foreign body in ear (T16)
   6. Head injury (S09.0)
   7. Injury (T14.9)
   8. Superficial injury (T14.0)
   9. Poisoning (T50.9)
   10. Burn of shoulder and upper limp (T22.0)
   11. Burn of ankle and foot (T25)
   12. Burn of head and neck (T20)
   13. Burn of hip and lower limb (T24)
   14. Burn of trunk (T21)
   15. Burn of wrist and hand (T23)
4. Presentations with a sole diagnosis unlikely to be affected by nutritional intervention (Congenital/Genetic condition) including following ICD-10 codes used in KEMReS:
   1. Congenital heart disease (Q25)
   2. Congenital laryngomalacia (Q31.5)
   3. Umbilical hernia (K42)
   4. Dislocation of hip (S73.0)
   5. Achondroplasia (Q77.4)
5. Follow up dressing (Z48.9) visits
6. Revisits within 1 week of recruitment diagnosis: New diagnosis versus recrudescence

For the calculation of the number of recurrent events above, we will not include revisits within 7 days of recruitment (Pilot work in respiratory infections showed that most infections resolve within a week). Recurrent events can either be caused by a new illness or recrudescence. We are not able to distinguish between the two in this trial.

We will not exclude child welfare examination clinic visits where a diagnosis was made, i.e. the child was sick.

### Analysis of intervention effects

For the primary analysis we will employ an intention-to-treat based analysis. We will however exclude those individuals who were recruited mistakenly, i.e. who were not eligible so whom we did not intend to treat.

Each analysis will address two separate questions: (i) do the groups who receive micronutrient supplements perform better than the group that received placebo, i.e. we will contrast the 6-week and 12-week groups with the placebo and (ii) do those receiving 12wk supplementation fare better than those receiving the supplement for only 6 weeks. The first of these will be addressed by representing treatment as a single indicator variable differentiating those randomised to receive any supplement from placebo. The second will introduce a further indicator variable taking the value 1 if randomised to the 12-week intervention and zero for those allocated to 6 weeks of intervention.

As the primary outcome we wish to estimate the factor by which treatment reduces the number of return visits as detailed above and test whether it differs from one. We will fit this parameter in a negative binomial model (with a log link function, i.e. a multiplicative model) relating the number of clinic visits to treatment group and known predictors of clinic visits:

1. Distance to nearest health clinic
2. Distance to MCR Keneba clinic
3. Age
4. WHZ at recruitment
5. Season
6. Sex
7. Location

We want to emphasize that our motivation for controlling for these variables is to reduce noise rather than remove confounding, which should not be an issue in a randomised study. We are aware that controlling for covariates can change the nature of the outcome examined but do not feel that the inclusion of the above covariate changes the interpretation of the proportional differences we are measuring here.

Distance to clinic and age will be fitted using orthogonal polynomials of these variables up to degree 3. Season will be fitted using truncated Fourier series.^4^

Because a reduction in overall clinic visits is of primary public health importance, we will also show the percentage of clinic visits analysed of the overall clinic attendances of the subjects and calculate the possible overall public health impact. We are, of course, aware that these children would have been seen more than the normal population, who was not enrolled in a trial.

## Growth

Internal Z-scores including our primary anthropometric indicator of interest HAZ (based on anthropometry data collected in the Keneba clinic between Nov 2009 and Feb 2012) will be calculated for each anthropometric measurement. Separate analyses of z-scores at each time point will use multiple regression controlling for baseline measurement (i.e. we are looking at growth rather than overall size). The purpose of these regressions is to plot the effect size versus time since recruitment in order to visualise any patterns in the timing of the treatment effect.

We will fit the following known predictors in the regression analysis:

a) Season

b) WHZ at recruitment

c) Sex

d) Age (when appropriate)

We will also pool the data from all time points and analyse them with a mixed model (capturing individual differences as random effect for both intercept and slope). Since we expect growth differences to increase with time, we will fit treatment effects (two as before) as interaction terms with time in the study but with no main effect of treatment. In this case the treatment effect term differentiating the 6- and 12-week regimes will take the value zero until 6-weeks after recruitment since these two groups receive identical treatment up to that point.

### Growth data cleaning

All growth data was entered directly into KEMReS with double entry and will be extracted and exported into Excel Spreadsheet. At the follow-up time points the measurements closes to the actual follow-up date will be chosen. Outlying measurements will be detected by

1. Scatter plots

AND

1. Calculating changes in Z-scores (WHZ, WAZ, HAZ, MAZ, SFTAZ) between measurement points of >3 standard deviations. We will remove the implausible measurements from the analysis and report on the numbers so removed.

# Subgroup analysis

We will distinguish between “a priori” and “ad hoc” effect modifiers and concentrate on “a priori” effect modifiers. We will demonstrate not just that treatment is significant in a particular subgroup but also that the effect differs significantly between subgroups, i.e. that the treatment subgroup interaction is significant. We believe that analysis of the interaction is essential since the proposal that there is a main effect within a particular subgroup is a change of hypothesis and needs to be justified.

## Assessment of effect modification

We consider the following baseline variables to be “a priori” modifiers of the effects of supplementation with multiple micronutrients for both primary outcomes: clinic visits and growth:

| Potential effect modifier | Indicator of | Putative mechanism |
| --- | --- | --- |
| Age | Indicator of acquired immunity against infectious disease | The ability of MMN to enhance immunity it most likely dependent on the level of acquired immunity prior to supplementation. |
| Stunting (HAZ) | Indicator of zinc status and other micronutrients | Absorption and turn over of supplemental zinc are increased in deficiencies of zinc and other micronutrients |
| Wasting (WHZ) | Indicator of malnutrition | Malnutrition increases risk of infections |

For continuous effect modifiers we first calculate their orthogonal polynomials up to degree 3. These will then be included in the regression model as main effects but only the interaction term between the first of these (the linear term) and treatment contrasts will be fitted. As before, the contrasts between any supplement and placebo and between 6 and 12 weeks supplementation will be considered separately. These interactions will be considered for both revisit and growth outcomes; under the null hypothesis the effects of treatment will be assumed to have an additive effect on z-scores and multiplicative on the renumber of return visits to the clinic.

A complication arises with HAZ as outcome when the data are pooled over all time points. For this analysis the treatment effect is already an interaction and is not a binary variable. In this case the interaction term will be the product of the treatment term and the linear polynomial of the effect modifier.

# Secondary analysis of morbidity

## Effect of supplementation on certain disease outcomes

Group analysis of different infectious disease outcomes after recruitment will be undertaken. It has been suggested in the past that multiple micronutrient supplementation may influence the incidence of respiratory and diarrhoeal disease. It has also been suggested that respiratory and diarrhoeal symptoms are reduced following supplementation.

In this secondary analysis we will look at the frequency of respiratory diagnoses given and respiratory symptoms reported during revisits. Separately, we will look at the frequency of diarrhoeal illness as well as malaria and skin infections.

These analyses will use negative binomial regression as above, changing only which visits are counted.

### Case definitions

#### Respiratory disease

Revisits will be included if either a respiratory diagnosis was given or there was a respiratory symptom recorded.

Following diagnosis of respiratory infection (ICD-10 codes) will be included:

Common cold (J00)

Pneumonia (J18)

Bronchiolitis (J21)

Chronic bronchitis (J42)

Asthma (J45)

Status asthmaticus (J46)

Chronic sinusitis (J32)

Chronic suppurative otitits media (H66.3)

Croup (J05.0)

Epiglottitis (J05.1)

Nonsuppurative otitis media (H65)

Peritonsilar absecess (J36)

Suppurative otitis media (H66.0)

Tonsillitis (J03.9)

Tuberculosis (A15)

Or free texts of

‘Acute respiratory illness’

‘ARI’

‘Acute respiratory infection’

‘Resolving pneumonia’

Clinic visits with the following reported respiratory symptoms will be included despite no given respiratory diagnosis. (A child may present with respiratory symptoms but may have “Unknown diagnosis” (R69) as their diagnosis.)

1. Cough
2. Perceived rapid breathing
3. Wheezing

#### Diarrhoeal disease

Revisits will be included if either a diarrhoeal diagnosis was given or there was a diarrhoeal symptom recorded.

Following diagnosis of diarrhoeal infection (ICD-10 codes) will be included: Viral gastroenteritis (A08)

Bacterial intestinal infection (A04.9 and A04)

Cholera (A00)

Giardia (A07.1)

Intestinal helminthiasis (B82.0)

Salmonella enteritis (A02.0)

Shigellosis (A03)

Or free texts of

‘diarrhoea’

‘diarrhoeal disease’

Furthermore, all cases with reported symptom of diarrhoea (≥3 loose stools per day) but no given diagnosis (see list above) exists. (A child may present with diarrhoea but may have “Unknown diagnosis” (R69) as their diagnosis.)

If there is a positive blood film the visit will only be included in malaria dataset. If the visit meets the definition of respiratory disease the visit is only included in respiratory disease dataset.

#### Malaria

We will run two analyses:

1. Include any visit with a positive slide for malaria OR Rapid Malaria Test for plasmodium falciparum AND history of fever or fever on presentation. We will not include any given diagnosis of malaria without a positive malaria slide OR Rapid MalariaTest.
2. Include any visit with a diagnosis of malaria

Diagnostic codes included:

Plasmodium falciparum malaria (B50)

#### Skin infections

We will include any visit that has any of the following diagnoses:

Cellulitis (L03)

Cold sore (B001)

Cutaneous abscess, furuncle and carbuncle (L02.9)

Impetigo (L01)

Local infection of skin & subcutaneous tissue (L08.9)

Post injury infected wound (T79.3)

Ring worm (B35.9)

Unspecified Rash (R21)

## Effect of supplementation on severity of representation

### Severity score and definitions

For the calculation of severity at representation we have developed a severity score that is based on the PAWS score (Paediatric Advanced Warning Score).

Details of severity scoring using KEMReS:

#### Definition of severity

Illness severity will be calculated using a number of categories:

1. **History and examination findings**

The WHO definitions of severe malaria, severe pneumonia and bronchiolitis, severe sepsis and severe diarrhoeal illness were adapted using equivalent criteria documented in the database to create a severity score, for each of the different diseases, for use in this study.^5^ All data entry fields were reviewed for relevant criteria.

1. ‘**Acutely ill-looking’**

As part of the clinical assessment, the clinicians document if the child was ‘acutely ill-looking’. Although subjective, this provides a useful overview of how the clinician felt the patient’s condition was.

1. **Parenteral treatment**

If the child with malaria needed IV/IM quinine or with diarrhoea needed IV fluids or with LRTI and sepsis needed IV antibiotics this suggests severe disease, as it is the recommended treatment for severe illness.^6^ For the analysis of severity of all disease groups, any IV/IM treatment will be included.

1. **Observation or referral**

This refers to whether the child was observed in the clinic observation bay or referred to a hospital or feeding centre. The reason these categories were not separated is that a patient will be entered in the system as for observation but it may not be updated if they are subsequently referred elsewhere.

1. **Early warning score**

A score based on observations at triage was used, which has been adapted to the setting (described further below).

For each of these criteria that are fulfilled one point will be awarded giving an overall severity score (see table 3). Each criteria provides additional information about the condition of the child, thus increasing the sensitivity of a severity score, though each has its own limitations. Having one positive classification would increase the sensitivity but would not be specific so a cut-off score of 2 or above was chosen to indicate severe illness.

Table 3. Severity score criteria^[[1]](#footnote-1)^

| **Diagnosis** | **History and examination findings** | **Acutely ill-looking** | **Parenteral treatment** | **Observation/ Referral** | **Early warning score** |
| --- | --- | --- | --- | --- | --- |
| **Malaria** | CNS disturbance- lethargy or convulsions or behaviour change or weak, not standing or apathetic or decreased coma score or jaundice or signs of anaemia (pallor or haemoglobin <5g/dl or haematocrit <15) or hypoglycaemia (blood glucose <2.5) | Acutely ill-looking | Quinine given | Observation/  Referral | 3+ |
| **LRTI** | Cyanosis or convulsions or lethargy or decreased coma score or behavioural change head nodding or signs of dehydration or chest wall indrawing or nasal flaring or grunting | Acutely ill-looking | IV antibiotics given | Observation/ referral | 3+ |
| **Diarrhoeal disease** | Weak not standing or lethargy or decreased coma score or behavioural change or not drinking/breastfeeding or signs of dehydration | Acutely ill-looking | IV fluids given | Observation/ referral | 3+ |
| **All diagnoses** | Any of the above | Acutely ill-looking | IV fluid  IV/IM antibiotics  IV/IM quinine | Observation / referral | 3+ |

For each column if the criteria are met, 1 point is given, the total out of 5 is the overall severity score.

#### Definition of the Early Warning Score

Triage observations offer a valuable insight into the severity of illness.^7^ The use of an Early Warning Score, although not in practice at the MRC clinic, is a good way of classifying illness severity.^8^ This study will use the PAWS score (Paediatric Advanced Warning Score), developed in 2008 by Egdell et al.^9^ This scoring system has been validated in an A&E setting in the UK, with a sensitivity of 70% and specificity of 90% for children needing intensive care admission.^9^ This system was chosen as the observations needed to calculate the score were available from the database or easily modified- for example changing the AVPU score to the coma score used in the clinic. The PAWS score was developed using the Advanced Paediatric Life Support guidelines.^10^ Points are given for abnormal observations and a score of 3 or greater indicates severe illness.

Table 2. How PAWS score is calculated^1^

|  | **Observation** | **Age group (months)** | **0 points** | **1 point** | **2 points** | **3 points** |
| --- | --- | --- | --- | --- | --- | --- |
| 1 | **Respiratory rate** (breaths per min) | 0-11 | 21-49 | 50-59 | <21  60-69 | 70+ |
|  |  | 12-23 | 16-44 | 45-54 | <16  55-64 | 65+ |
|  |  | 24-59 | 16-39 | 40-49 | <16  50-59 | 60+ |
|  |  | 60+ | 11-34 | 35-44 | <11  45-54 | 55+ |
| 2 | **Heart rate** (beats per min) | 0-11 | 91-179 | 71-90  180-199 | 51-70  200-219 | <51  220+ |
|  |  | 12-23 | 81-169 | 61-80  170-189 | 41-60  190-209 | <41  210+ |
|  |  | 24-59 | 76-159 | 56-75  160-179 | 36-55  180-199 | <36  200+ |
|  |  | 60+ | 61-139 | 41-60  140-159 | 21-40  160-179 | <21  180+ |
| 3 | **Work of breathing** |  | Normal |  | One of chest indrawing or accessory muscle use | Both chest indrawing and accessory muscle use |
| 4 | **Oxygen saturation levels** (%) |  | 93+ | 90-92 | 85-89 | <85 |
| 5 | **Body temperature** (°c) |  | 36-37.9 | 38-38.9 | 35-35.9  39+ | <35 |
| 6 | **Capillary refill time** (sec) |  | 0-3 |  | 4-6 | >6 |
| 7 | **Coma score** |  | 15/15 | 9-14/15 | 4-8/15 | 3/15 |

##### ^1^ For each of the 7 observations, points are given out of a maximum of 3. The total PAWS score is the combined points. If this is 3 or above this gives one point to the overall severity score.

The 6-point score will be used (0-5). Any score of two or above is considered as a severe illness presentation.

### Effect of supplementation on representation with severe illness

We will analyze first all severe illness revisits as for revisits in general (7.1.3).

As the primary outcome we wish to estimate the factor by which treatment reduces the number of return visits with severe illness and test whether it differs from one. As for all revisits we will fit this parameter in a negative binomial model (with a log link function, i.e. a multiplicative model) relating the number of clinic visits to treatment group and known powerful predictors of clinic visits:

1. Distance to nearest health clinic
2. Distance to MCR Keneba clinic
3. Age
4. WHZ at recruitment
5. Season
6. Sex
7. Location

### Effect of supplementation on representation with severe illness and certain disease outcomes

We will then undertake a secondary analysis for each of the following disease categories:

1. Malaria
2. Lower respiratory tract infections (LRTI)
3. Diarrhoeal disease

Malaria and Diarrhoeal disease are defined as in 9.1.1. Only addition is that for diarrhoeal disease if the visit meets definition of LRTI the visit will only be included in LRTI dataset.

LRTIs are defined in 9.2.3.1.

This analysis will use negative binomial regression as for all revisits with severe illness (9.2.1.1), changing only which visits are counted. Again, we will control for known powerful predictors of clinic visits:

1. Distance to nearest health clinic
2. Distance to MCR Keneba clinic
3. Age
4. WHZ at recruitment
5. Season
6. Sex
7. Location

#### Definition of LRTI

Diagnosis of ‘pneumonia (J18)’ or ‘Bronchilotis (J21) or “Acute respiratory infection (free text)’, ‘ARI’ or ‘Resolving pneumonia (free text)’.

Or

Cough and/or shortness of breath and/or chest pain and/or breathless AND raised RR and/or crackles and/or crepitations and/or decreased air entry and/or wheeze and/or signs of severe disease i.e. chest indrawing or nasal flaring or grunting or signs of dehydration or head nodding or lethargy or decreased coma score.

If there is positive blood film the visit will be included in the malaria dataset only. If there is a co-diagnoses of diarrhoeal disease, it will be included in LRTI dataset only.

## Effect of supplementation on symptom resolution of primary illness

We will analyze the effect of supplement on symptom resolution of the presenting illness at recruitment over 1 week.

Following reported symptoms were recorded daily over the first 7 days for each participant:

1. Diarrhoea (≥3 loose stools per day)
2. Vomiting (not associated with feeding/coughing)
3. Cough
4. Rapid breathing
5. Fever

### Graphic presentation of symptom resolution

First, we will present changes in symptoms with time and supplement. In a stacked bar chart each time point will be represented by one bar and each bar would be divided into sections representing the proportion of children with 0, 1, 2, 3, 4, 5 symptoms. Separate graphs will be drawn for each treatment group.

### Analysis of symptom resolution

We will calculate a symptom score (i.e. the total number of symptoms recorded: diarrhoea, vomiting, cough, rapid breathing and fever) for each child for each of the first seven days of the study.

We will also separate out respiratory and gastrointestinal (GI) symptoms by using a score for each on a three-point scale (0= no respiratory symptoms, 1 = respiratory symptoms, no fever, 2 = respiratory symptoms and fever and similarly for GI symptoms). We will perform three analyses: (1) respiratory symptoms, (2) GI symptoms and (3) all symptoms score.

These will then be analyzed using ordered logistic regression in which we allow for correlation between measurements on the same individual by employing Huber-White robust standard errors. As with the analysis of growth (2.2), we will fit the effects of treatment as an interaction with time since recruitment (with no main treatment effect). We will only compare the placebo group with the rest since the 6- and 12-wk groups received identical treatment in this phase of the study.

We will fit the following covariates as a main effect:

1. Age
2. WHZ at recruitment
3. Sex
4. Season

## Effect of supplementation on reported morbidity during supplementation

During the supplementation period of 12 weeks, following reported morbidity entities were recorded fortnightly:

1. Diarrhoea (≥3 loose stools per day)
2. Vomiting (not associated with feeding/coughing)
3. Cough
4. Rapid breathing
5. Fever

These data would be analysed as a score calculated in the same way as the symptom resolution data. In this case, however, we will consider the treatment effects as simple main (intercept) effects and will not model time since recruitment. In this case the 6- and 12-week groups do differ but only after 6-weeks: therefore, similarly to the analysis of growth, a binary variable, taking the value one if the individual is in the 6-week group and the time point is beyond 6 weeks since recruitment and zero otherwise, will model this difference.

We will fit the following covariates as a main effect:

1. Age
2. WHZ at recruitment
3. Sex
4. Season

# Secondary analysis of micronutrient status

We will analyze the effect of supplementation on micronutrient status at 12 weeks, i.e. at the end of the supplementation period.

Following blood levels of analytes were measured:

1. Plasma 25(OH)D
2. Plasma retinol
3. Plasma zinc
4. Plasma selenium
5. Haemoglobin

Effect of supplementation on each micronutrient measured will be analyzed by multiple regression. Those micronutrients whose distributions are skewed (with long right-hand tail) will be log-transformed as appropriate – this will certainly be required for vitamin D, zinc, selenium and retinol. Effect sizes in these cases will be presented as percent, rather than absolute differences in concentration. We will control for the following co-variates:

1. Age
2. WHZ at recruitment
3. Sex
4. Season
5. CRP
6. AGP

# Secondary analysis of the effect of supplementation on reported appetite

## Appetite during first week after recruitment

We will analyze the effect of supplementation on reported appetite over the first 7 days after initial presentation. We will compare intervention (6-week and 12-week supplementation) versus placebo only.

During each visit over the first 7 days the mothers were specifically questioned regarding their child’s appetite. Allowable responses were: ‘Same as usual’, ‘Less than usual’, or ‘Greater than usual’.

Initially, we will show graphically for each group the proportion of anorexia (‘Less than usual’), normal and improved appetite on each day by group.

We will then use a score based on the sum of responses (-1, 0 and 1 for worse, normal and better respectively) over the first seven days and analyse the score by least squares regression. We may have to change the error structure assumption if it is not normal. Initial analysis of the responses without taking any account of treatment group indicate that the normal assumption should be adequate, however.

We will control for the following co-variates:

1. Age
2. WHZ at recruitment
3. Sex
4. Season

## Appetite during supplementation period

We will analyze the effect of supplementation on appetite reported fortnightly during the 12-week supplementation period (compare the 3 intervention groups).

We will compare the mean of the appetite score over the 12-week period between the groups. This will be analysed similarly to 6.1 except that now will control for reported morbidity: each symptom will be fitted as a binary variable. Morbidity was reported fortnightly at the same time as appetite. Following morbidity measures will be included:

1. Unwell last 71 hours
2. Diarrhoea (≥3 loose stools per day)
3. Vomiting (not associated with feeding/coughing)
4. Cough
5. Rapid breathing
6. Fever

We will control for the following co-variates:

1. Age
2. WHZ at recruitment
3. Sex
4. Season

1. [↑](#footnote-ref-1)
