# Supplementary material for: Impact of fortified versus unfortified lipid-based supplements on morbidity and nutritional status: A randomised double-blind placebo-controlled trial in ill Gambian children
Source: PLoS Med. 2017 Aug 15;14(8):e1002377. doi: 10.1371/journal.pmed.1002377 (PMC5557358; doi:10.1371/journal.pmed.1002377)
Supplement: S5 Text — (DOCX) [file pmed.1002377.s006.docx]

**S3 Appendix**: **Standard Operating Procedures for Anthropometric Measurements**
