# Supplementary material for: Impact of fortified versus unfortified lipid-based supplements on morbidity and nutritional status: A randomised double-blind placebo-controlled trial in ill Gambian children
Source: PLoS Med. 2017 Aug 15;14(8):e1002377. doi: 10.1371/journal.pmed.1002377 (PMC5557358; doi:10.1371/journal.pmed.1002377)
Supplement: S1 Table — (DOCX) [file pmed.1002377.s007.docx]

**S1 Table:** Composition of small-quantity lipid-based multiple micronutrient supplement

|  |  |  |  |  |  |  |
| --- | --- | --- | --- | --- | --- | --- |
| **Nutrient** | **Chemical compound used** | **Unit** | **LNS-MMN-1** | **LNS-MMN-2** | **LNS-MMN-0** |  |
|  |  |  | 20g | 20g | 20g |  |
| **Potassium** | Potassium chloride | mg | 152 | 152 | 60 |  |
| **Calcium** | Calcium carbonate | mg | 200 | 220 | 20 |  |
| **Phosphorus (excluding phytate)** | Tri-Calcium Phosphate | mg | 82 | 82 | 35 |  |
| **Magnesium** | Magnesium carbonate | mg | 16 | 16 | 11 |  |
| **Iron** | Iron fumarate | mg | 12 | 12 | 0.4 |  |
| **Zinc** | Zinc oxide | mg | 10 | 20 | 0.2 |  |
| **Copper** | Copper (II) gluconate | mg | 0.4 | 0.6 | 0.02 |  |
| **Selenium** | Sodium selenite anhydrous | mcg | 20 | 40 | 1.3 |  |
| **Iodine** | Potassium iodide | mcg | 180 | 180 | 1.02 |  |
| **Manganese** | Manganese carbonate | mg | 0.08 | 0.08 | 0.06 |  |
| **Vitamin A** | Vitamin A palmitate | mcg | 400 | 400 | 3.6 |  |
| **Vitamin D** | Vitamin D3 | mcg | 10 | 20 | 0.0 |  |
| **Vitamin E** | Vitamin E | mg | 5.2 | 10 | 0.5 |  |
| **Vitamin K** | Vitamin K1 | mcg | 20 | 26 | 1·4 |  |
| **Vitamin B1** | Thiamine mononitrate | mg | 0.6 | 1.0 | 0.04 |  |
| **Vitamin B2** | Riboflavin universal / fermentative | mg | 0.8 | 1.2 | 0.04 |  |
| **Vitamin C** | Ascorbic acid | mg | 60 | 60 | 0.9 |  |
| **Vitamin B6** | Pyridoxine hydrochloride | mg | 0.6 | 1.2 | 0.02 |  |
| **Vitamin B12** | Vitamin B12 | mcg | 1.0 | 2.0 | 0.04 |  |
| **Folic acid** | Folic acid food grade | mcg | 160 | 320 | 7 |  |
| **Niacin** | Niacinamide | mg | 8 | 16 | 0.3 |  |
| **Pantothenic acid** | Calcium D-Pantothenate | mg | 3.6 | 5 | 0.08 |  |
| **Biotin** | IS-846 Biotin | mcg | 12 | 20 | 0.9 |  |
|  |  |  |  |  |  |  |
