# Supplementary material for: Impact of fortified versus unfortified lipid-based supplements on morbidity and nutritional status: A randomised double-blind placebo-controlled trial in ill Gambian children
Source: PLoS Med. 2017 Aug 15;14(8):e1002377. doi: 10.1371/journal.pmed.1002377 (PMC5557358; doi:10.1371/journal.pmed.1002377)
Supplement: S2 Table — (DOCX) [file pmed.1002377.s008.docx]

**S2 Table:** Severe adverse events (SAE)* by treatment group

|  |  | **MMN-0** | **MMN-6** | **MMN-12** | Causal relationship with IMP** |
| --- | --- | --- | --- | --- | --- |
|  | **Total SAE** | n=20 | n=21 | n=20 |  |
|  |  |  |  |  |  |
| **Diagnosis** | **ICD-10** |  |  |  |  |
| Pneumonia | J18 | 2 | 8 | 6 | Unlikely |
| Bronchiolitis | J21 | 0 | 1 | 2 | Unlikely |
| Gastroenteritis | A08 | 1 | 5 | 3 | Unlikely |
| Bacterial intestinal infection | A04.9 | 1 | 0 | 1 | Unlikely |
| Marasmus Kwashiorkor | E42 | 1 | 1 |  | Unlikely |
| Kwashiorkor | E40 | 1 | 0 | 1 | Unlikely |
| Marasmus | E41 | 3 | 0 | 0 | Unlikely |
| Malaria | B50 | 1  (Death with severe malaria and anaemia) | 1 | 1 | Unlikely |
| Septicaemia | A41.9 | 4 | 2 | 4 | Unlikely |
| Death unknown cause |  | 0 | 1 | 0 | Unlikely |
| Unknown diagnosis | R69 | 0 | 1 | 1 | Unlikely |
| Acute infective hepatitis | B19 | 1 | 0 | 0 | Unlikely |
| Meningitis | A87 | 1 | 0 | 0 | Unlikely |
| Septic arthritis | M13.9 | 1 | 0 | 0 | Unlikely |
| Orbital Cellulitis | H05.0 | 1 | 0 | 0 | Unlikely |
| Suppurative otitis media | H66.0 | 1 | 1 | 0 | Unlikely |
| Conjunctivitis | H10.9 | 0 | 0 | 1 | Unlikely |
| Burn | T23 | 1 | 0 | 0 | Unlikely |

^*^Serious adverse events were classified as any illness presentation during the 12-week supplementation period, which required admission to the observation room or transfer to another hospital or death. ^**^As assessed by the trial safety monitor.
